# Dose-response of acupuncture on ovulation rates in polycystic ovary syndrome: a meta-analysis and exploratory dose-response analysis

**DOI:** 10.3389/fendo.2025.1610338

**Published:** 2025-08-28

**Authors:** Jiale Wei, Zheng Shen, ChunYan Zhao, ChunLin Xie, HongFa Bai, JiaHui Yin, Jian Wang

**Affiliations:** ^1^ Shandong University of Traditional Chinese Medicine, Jinan, China; ^2^ The Affiliated Hospital of Shandong University of Traditional Chinese Medicine, Jinan, China

**Keywords:** acupuncture, polycystic ovary syndrome, ovulation, meta-analysis, dose-response analysis

## Abstract

**Objective:**

This study aimed to evaluate the efficacy of acupuncture in improving ovulation rates in women with polycystic ovary syndrome (PCOS) and to identify optimal dosage parameters, including the number of acupoints, treatment frequency, and session duration, using integrated pairwise meta-analysis, network meta-analysis (NMA), and model-based dose-response modeling.

**Methods:**

Nine databases were searched up to January 2025, yielding 43 randomized controlled trials (RCTs) involving 4,827 participants that compared acupuncture with sham acupuncture, pharmacotherapy, or conventional therapy control group. Pairwise meta-analysis, NMA, and model-based dose-response modeling were performed.

**Results:**

Acupuncture alone significantly increased ovulation rates compared with sham acupuncture (RR = 1.15, 95% CI: 1.04-1.27) and pharmacotherapy (RR = 1.11, 95% CI: 1.04-1.20). Additionally, acupuncture combined with herbal medicine outperformed pharmacotherapy (RR = 1.27, 95% CI: 1.12-1.43). NMA ranked acupuncture combined with herbal medicine as the most effective intervention (SUCRA = 97.8%). Dose-response modeling identified the following optimal protocols: for acupuncture alone, 30 minutes per session, 29 acupoints, three sessions per week for 24 weeks; and for combined therapy, 19 minutes per session, 26 acupoints, four sessions per week for 24 weeks.

**Conclusion:**

Acupuncture is an effective non-pharmacological intervention for PCOS-related ovulatory dysfunction, with its efficacy dependent on precise dosing parameters. These findings highlight the need for standardized protocols in future trials to validate dose-response thresholds and to optimize personalized treatment strategies.

**Systematic review registration:**

www.crd.york.ac.uk, identifier PROSPERO (CRD420250651353).

## 1 Introduction

Polycystic ovary syndrome (PCOS), a complex endocrine disorder affecting 4-21% of reproductive-aged women globally, is diagnosed using the Rotterdam criteria, which require at least two of the following: hyperandrogenism (clinical or biochemical), irregular menstrual cycles, and polycystic ovarian morphology (1, 2). The disorder’s multifactorial etiology encompasses genetic polymorphisms (e.g., CYP gene family variants), environmental exposures to endocrine-disrupting chemicals [EDCs, including bisphenol A (BPA), dichlorodiphenyltrichloroethane (DDT), and mercury], high-calorie dietary intake, physical inactivity, and gut microbiota dysbiosis (3, 4). The triad of insulin resistance (IR), gonadotropin dysregulation, and chronic low-grade inflammation mechanistically drives PCOS pathogenesis. IR, affecting approximately 70% of patients, leads to compensatory hyperinsulinemia, which stimulates ovarian androgen secretion while suppressing hepatic sex hormone-binding globulin (SHBG) synthesis. Concurrently, aberrant lipid metabolism exacerbates IR, establishing a self-perpetuating cycle of metabolic dysfunction (5, 6). Gonadotropin dysregulation manifests as increased pulsatile secretion of gonadotropin-releasing hormone (GnRH), elevating the luteinizing hormone (LH)/follicle-stimulating hormone (FSH) ratio. This hormonal imbalance promotes excessive androgen production by ovarian theca cells, contributing to hyperandrogenemia (7). Finally, dysregulated immune cells and elevated inflammatory cytokines in serum and ovarian tissues induce systemic low-grade chronic inflammation (SLCI), which synergistically interacts with obesity, hyperandrogenemia, and IR to amplify metabolic and reproductive dysfunction (8). Collectively, these mechanisms perpetuate the heterogeneous clinical manifestations of PCOS.

Clinically, these pathophysiological disturbances manifest as obesity (present in 53-74% of cases) and hyperandrogenemia (in approximately 60% of cases). Furthermore, PCOS increases the risk of several comorbid conditions, including a 2.87-fold higher risk of type 2 diabetes (95% CI: 1.37-6.01), a 1.72-fold higher risk of hypertension (95% CI: 1.43-2.07), an approximately 10-fold higher risk of obstructive sleep apnea (95% CI: 3.90-23.26), and a 1.68-fold higher risk of cardiometabolic diseases (95% CI: 1.26-2.23) (9–11). Oral contraceptives are the most commonly prescribed therapy for PCOS. Clomiphene citrate, a non-steroidal selective estrogen receptor modulator (SERM), and letrozole, an aromatase inhibitor, are widely used to induce ovulation. Additionally, metformin is employed to ameliorate IR in patients with PCOS (12). However, due to adverse effects and poor adherence associated with oral pharmacotherapies (13–15), the exploration of alternative therapeutic approaches has become imperative.

Due to the limited efficacy and risks of conventional pharmacological treatments, non-pharmacological approaches like acupuncture have attracted growing interest for their potential to improve reproductive and metabolic dysfunction in PCOS via multimodal mechanisms (16). Rooted in traditional Chinese medicine (TCM) theory, acupuncture aims to restore balance to the “Kidney-Tiangui-Chongren” axis, a conceptual framework corresponding to dysregulation of the hypothalamic-pituitary-ovarian (HPO) axis in biomedical terms (17). Recent studies show that electroacupuncture downregulates ovarian *Alas2* expression, modulates autophagy, and regulates the kisspeptin-GnRH/LH axis, thereby alleviating clinical symptoms of PCOS (18–20). However, some studies report nonsignificant therapeutic effects of acupuncture, likely due to methodological limitations in trial design, such as acupoint selection, treatment duration, control group allocation, and suboptimal statistical analyses (21).

While current evidence supports the therapeutic potential of acupuncture in PCOS, its dose-response relationships remain underexplored. Existing systematic reviews have predominantly focused on pairwise comparisons between acupuncture and single comparator interventions, without comprehensively assessing the efficacy hierarchy of acupuncture, pharmacological therapies, and their combined regimens in improving ovulation rates. To address these limitations, this study employs a dual analytical framework combining conventional pairwise meta-analysis with network meta-analysis and model-based dose-response modeling. This approach aims to hierarchically rank therapeutic efficacy and elucidate nonlinear relationships between key acupuncture dosage parameters (acupoint quantity, treatment frequency, and duration) and ovulation outcomes. The findings are expected to optimize acupuncture protocols by identifying thresholds for maximal benefit, thereby guiding clinical practice and informing randomized controlled (RCT) design for PCOS management.

## 2 Materials and methods

In accordance with the Preferred Reporting Items for Systematic Reviews and Meta-Analyses (PRISMA) guidelines, this study was designed to follow established protocols for systematic reviews and meta-analyses. The methodology complied with the PRISMA Statement for standard pairwise meta-analysis, while components related to the NMA were guided by the corresponding PRISMA extension. Both frameworks were implemented rigorously to ensure methodological consistency and strict adherence to contemporary academic standards. The study protocol was prospectively registered with PROSPERO (CRD420250651353).

### 2.1 Eligibility criteria and outcomes

This systematic review included studies comparing acupuncture with sham acupuncture, conventional Western medications (e.g., metformin, letrozole, clomiphene), or conventional therapy control groups in patients with PCOS. Conventional therapy control groups were defined as those receiving the same adjunct therapies alone, serving as comparators for interventions combining acupuncture with these therapies. Adjunct treatments included Western medications (e.g., metformin, letrozole, clomiphene) and conventional Chinese herbal medicine. Additionally, comparisons between acupuncture combined with herbal medicine and conventional Western drugs were conducted to explore the synergistic effects consistent with the TCM principle of integrating acupuncture and herbal therapy. Studies were included if they met the following criteria: (1) RCTs published in English or Chinese; (2) participants diagnosed with PCOS according to the 2003 Rotterdam consensus criteria (22); (3) intervention groups receiving acupuncture alone or combined with herbal medicine; (4) control groups receiving sham acupuncture or conventional pharmacological treatments; and (5) Reporting ovulation rate as a primary or secondary outcome measure.

The exclusion criteria were as follows: (1) studies involving participants with concurrent endocrine or reproductive disorders, including hyperprolactinemia, thyroid dysfunction, premature ovarian failure, androgen-secreting adrenal or ovarian tumors, hypothalamic or pituitary amenorrhea, or other conditions associated with hyperandrogenism, menstrual irregularities, or ovulatory dysfunction; (2) non-randomized studies, including reviews, animal or *in vitro* studies, conference abstracts, meta-analyses, retrospective analyses, case reports, or non-primary research; (3) studies with fewer than 20 participants; (4) duplicate publications; and (5) studies with incomplete abstract information or inaccessible raw data.

The primary outcome measure was the ovulation rate, calculated as follows: Ovulation Rate (%) = (Number of Ovulatory Cycles ÷ Total Observed Cycles) × 100%. Secondary outcomes included levels of sex hormones (FSH, LH, and testosterone) and body mass index (BMI).

### 2.2 Data sources and searches

Five English databases (i.e., PubMed, Web of Science, Embase, Cochrane Library, and Ovid Medline) and four Chinese databases (CNKI, WanFang, VIP, and CBM) from their inception to 17 January 2025 were searched. Relevant terminology related to acupuncture and PCOS was first identified through the MeSH database of the National Center for Biotechnology Information (NCBI) to establish standardized descriptors and entry terms. When multiple MeSH terms appeared, selections were made based on conceptual relevance to the study objectives. The search strategy was then refined across databases to enhance retrieval precision, using truncation to capture lexical variations and removing redundant terms to streamline the algorithm. This process aimed to improve methodological rigor and reproducibility (see **Supplementary File 1**).

### 2.3 Selection of studies and data extraction

Two reviewers (W.J.L. and Z.C.Y.) independently screened all abstracts and full-text articles, with discrepancies resolved through iterative consensus discussions. Data extraction was conducted using prespecified standardized forms to systematically capture authorship, publication year, trial design, participant characteristics (e.g., age, diagnostic criteria), intervention protocols, follow-up duration, and primary and secondary outcome measures. Persistent discrepancies during data extraction were adjudicated by a third reviewer (W.J.) to ensure methodological rigor.

### 2.4 Risk of bias and certainty of evidence

Two reviewers (W.J.L. and Z.C.Y.) independently assessed the risk of bias in the included trials. The updated version of the ROB 2.0 assessment tool recommended by the Cochrane Collaboration was used, which covers five domains: (1) randomization process, (2) deviations from intended interventions, (3) missing outcome data, (4) measurement of outcomes, and (5) selection of reported results (23) (see **Supplementary File 2**). The quality of evidence for the pairwise meta-analyses was assessed using the Grading of Recommendations, Assessment, Development, and Evaluations (GRADE) framework (24). Downgrading factors considered included risk of bias, inconsistency, indirectness, imprecision, and publication bias (25) (see **Supplementary File 11**). The Confidence in NMA (CINeMA) framework was applied to evaluate the certainty of evidence derived from the NMA (26). This framework assessed six methodological domains: (1) within-study bias, (2) reporting bias (including publication bias and selective outcome reporting), (3) applicability of evidence, (4) precision of estimates, (5) between-study heterogeneity, and (6) network inconsistency. CINeMA employed a three-tier classification system (low risk, some concerns, or high risk) to assess methodological rigor within each domain. These domain-specific evaluations were subsequently aggregated to derive a four-tier confidence categorization (high, moderate, low, or very low) for all comparative treatment effects (27).

### 2.5 Assessment of acupuncture dose

Dosage quantification was determined using four predefined criteria (28), with thresholds established based on the median distribution of parameters observed in the included studies, ensuring a balanced stratification for analysis: (1) number of acupoints per session: >13 acupoints (high dose) vs. ≤13 acupoints (low dose); (2) Deqi response: presence (high dose) vs. absence (low dose) of the characteristic needling sensation; (3) treatment frequency: >4 sessions/week (high dose) vs. ≤4 sessions/week (low dose); and (4) intervention duration: >12 weeks (high dose) vs. ≤12 weeks (low dose).A scoring system assigned +1 point for each high-dose criterion and -1 point for each low-dose criterion. Cumulative scores categorized interventions into three groups: (1) high dose: aggregate scores of +2 to +4; (2) moderate dose: scores of -1 to +1; and (3) low dose: aggregate scores of -4 to -2. Two independent reviewers evaluated each criterion, with unresolved discrepancies adjudicated by a third reviewer. To mitigate bias, reviewers were blinded to each other’s assessments throughout the process.

### 2.6 Data analysis

#### 2.6.1 Pairwise meta-analysis

Meta-analyses for dichotomous outcomes (ovulation rate) were conducted using the “meta” package in R (version 4.4.2). Heterogeneity was assessed using the I^2^ statistic, with *p* < 0.05 or I^2^ > 50% indicating statistically significant heterogeneity. A random-effects model was selected when I^2^ < 50%; otherwise, a fixed-effects model was applied to ensure robustness. Sensitivity analysis was performed using the ‘metainf’ function with a leave-one-out approach to evaluate the stability of the results. Additionally, all pairwise comparisons required at least two studies for inclusion. Covariates including age, number of acupoints, acupuncture frequency, acupuncture type, retaining time, and acupuncture dose were incorporated into univariate meta-regression and subgroup analyses to explore their influence on outcomes. For analyses involving ≥10 studies, publication bias was assessed using Egger’s test and funnel plots. Symmetrical funnel plots with *p* > 0.05 were interpreted as indicating no significant publication bias (see **Supplementary File 3**).

#### 2.6.2 NMA

NMA was conducted using the “netmeta” package in R (version 4.4.2). Heterogeneity was evaluated using the I^2^ statistic, with a random-effects model selected if *p* < 0.05 or I^2^ > 50%; otherwise, a fixed-effects model was applied. Given the complexity of evidence structures in NMA, inconsistency between direct and indirect evidence was assessed using the node-splitting method in addition to heterogeneity testing. This method evaluates the agreement between direct and indirect evidence at specific split nodes, with *p*-values > 0.05 indicating no significant inconsistency. Node-split analyses were performed to quantify inconsistency for each comparison. Interventions were ranked based on the surface under the cumulative ranking curve (SUCRA) to evaluate their efficacy in improving ovulation rates in patients with PCOS. Additionally, NMA meta-regression using the “gemtc” package examined the influence of covariates, including age, number of acupoints, acupuncture frequency, acupuncture type, treatment duration, acupuncture dose, and needle retention time, on outcomes and heterogeneity.

#### 2.6.3 Exploratory acupuncture dose-response analysis

Dose-response analysis was conducted using the “MBNMAdose” package in R (version 4.4.2). This Bayesian dose-response modeling framework, termed Model-Based NMA for Dose-Response Relationships (MBNMA), integrates multiple dose levels into a unified structure by simulating diverse dose-response functions. By modeling dose-response relationships, this approach connects otherwise disconnected evidence networks and enhances the precision of treatment effect estimates (29).

To validate the consistency assumption of the model, a NMA model was fitted that does not assume consistency, exclusively modeling the direct relative effects between each treatment arm and the reference treatment within individual studies. The results of this NMA model were compared with those derived from the Unrelated Mean Effects (UME) model. A discrepancy between direct and indirect evidence within the network would indicate that the consistency assumption should be reconsidered (30).

The Deviance Information Criterion (DIC) values of spline functions, log-linear functions, and Emax functions, each modeled under both random-effects and fixed-effects frameworks, were compared to identify the optimal functional form balancing goodness-of-fit and model complexity. Covariates, including the number of acupoints, acupuncture frequency, and dose group classification, were incorporated into the analysis.

## 3 Results

### 3.1 Studies selection and characteristics of include trails

**Figure 1:**
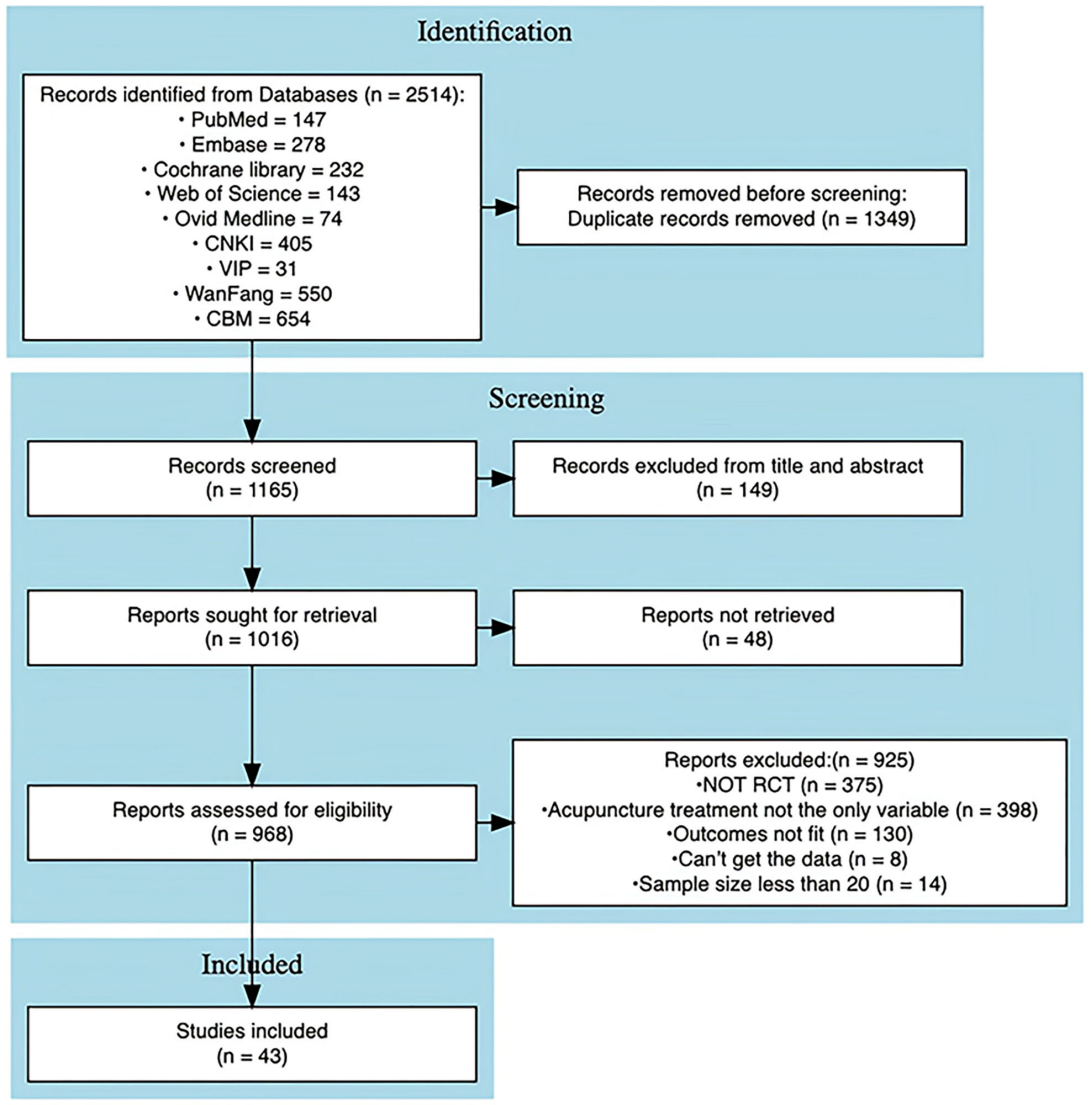
Flowchart for the selection of randomized trials.

A total of 2,514 studies were identified through database searches. After removing 1,349 duplicate records, 149 studies were excluded following title and abstract screening. Of the remaining 1,016 studies, 925 were excluded after independent full-text review, and 48 studies (31–73) were unavailable for retrieval. Ultimately, 43 studies were included in the final analysis. Reasons for exclusion at each screening stage are detailed in **Figure 1**. The included studies were published between 2005 and 2024, with 40 studies (approximately 93.0%) published within the last decade (2015-2024), indicating the currency of the evidence. The mean age of participants across the included studies was 27.82 years. All studies reported ovulation rate as an outcome measure, with 7 studies reporting BMI, 25 reporting FSH, 30 reporting LH, and 14 reporting the LH/FSH ratio. Among the 35 studies that included conventional pharmacological treatments as a comparator group, the specific Western medications varied. Clomiphene citrate was the most frequently used medication, appearing in 19 studies (54.3%), either alone or in combination with other drugs. Letrozole was included in 7 studies (20.0%), Diane-35 (ethinyl estradiol/cyproterone acetate) in 8 studies (22.9%), and metformin in 6 studies (17.1%). Detailed characteristics of the included trials are provided in **Supplementary File 4**.

### 3.2 Risk of bias and certainty of evidence

**Supplementary File 5** summarizes the risk of bias assessment for the included trials. Three studies (7%) were rated as “low risk” across all domains. Twelve studies (28%) raised “some concerns” in the randomization process due to insufficiently described allocation concealment, while 28 studies (65%) exhibited deficiencies in the blinding of participants and outcome assessors. One study was categorized as “some concerns” due to attrition rates between 10% and 20%. Four studies (9%) that were registered in clinical trial registries with publicly accessible protocols were classified as “low risk.” The CINeMA evaluation indicated high certainty of evidence for only a minority of comparisons (e.g., acupuncture plus medicine vs. sham acupuncture). Most comparisons were rated as very low certainty due to within-study bias, heterogeneity, or imprecision, highlighting the limited reliability of the existing evidence base. In the pairwise meta-analyses, the GRADE framework was employed to appraise the quality of evidence. The findings indicate that most evidence supporting ovulation rate outcomes was of high quality. However, moderate-quality evidence was noted in comparisons of acupuncture combined with Chinese herbal medicine versus conventional pharmaceuticals, and acupuncture versus routine treatment controls. These downgrades were primarily due to wide confidence intervals in the former and potential publication bias in the latter (**Supplementary File 12**). Conversely, when applying the CINeMA framework to assess evidence certainty in network meta-analyses, most comparisons were downgraded to very low certainty. This discrepancy reflects the fundamental differences between GRADE and CINeMA: GRADE focuses on the internal validity of direct comparisons, while CINeMA evaluates the certainty across the entire evidence network, incorporating both direct and indirect evidence. High heterogeneity, potential inconsistencies, and uncertainties in indirect comparison pathways were the primary factors contributing to the overall low certainty of network evidence, even when individual direct comparisons were of relatively high quality.

#### 3.2.1 Acupuncture dose

The number of acupoints used across studies ranged from 2 to 29, with the majority (72%, 31 trials) employing 11–17 acupoints. All studies explicitly confirmed the presence of deqi. Acupuncture frequency varied from once weekly to once daily, with most trials (70%, 30 trials) administering either three or seven sessions per week. The intervention duration spanned 4 to 24 weeks, with the majority (72%, 31 trials) utilizing a 12-week protocol. Based on predefined criteria, the acupuncture dosages in the included studies were categorized as high dose (21%, 9 trials), moderate dose (58%, 25 trials), and low dose (21%, 9 trials).

### 3.3 Pairwise meta-analysis

**Figure 2:**
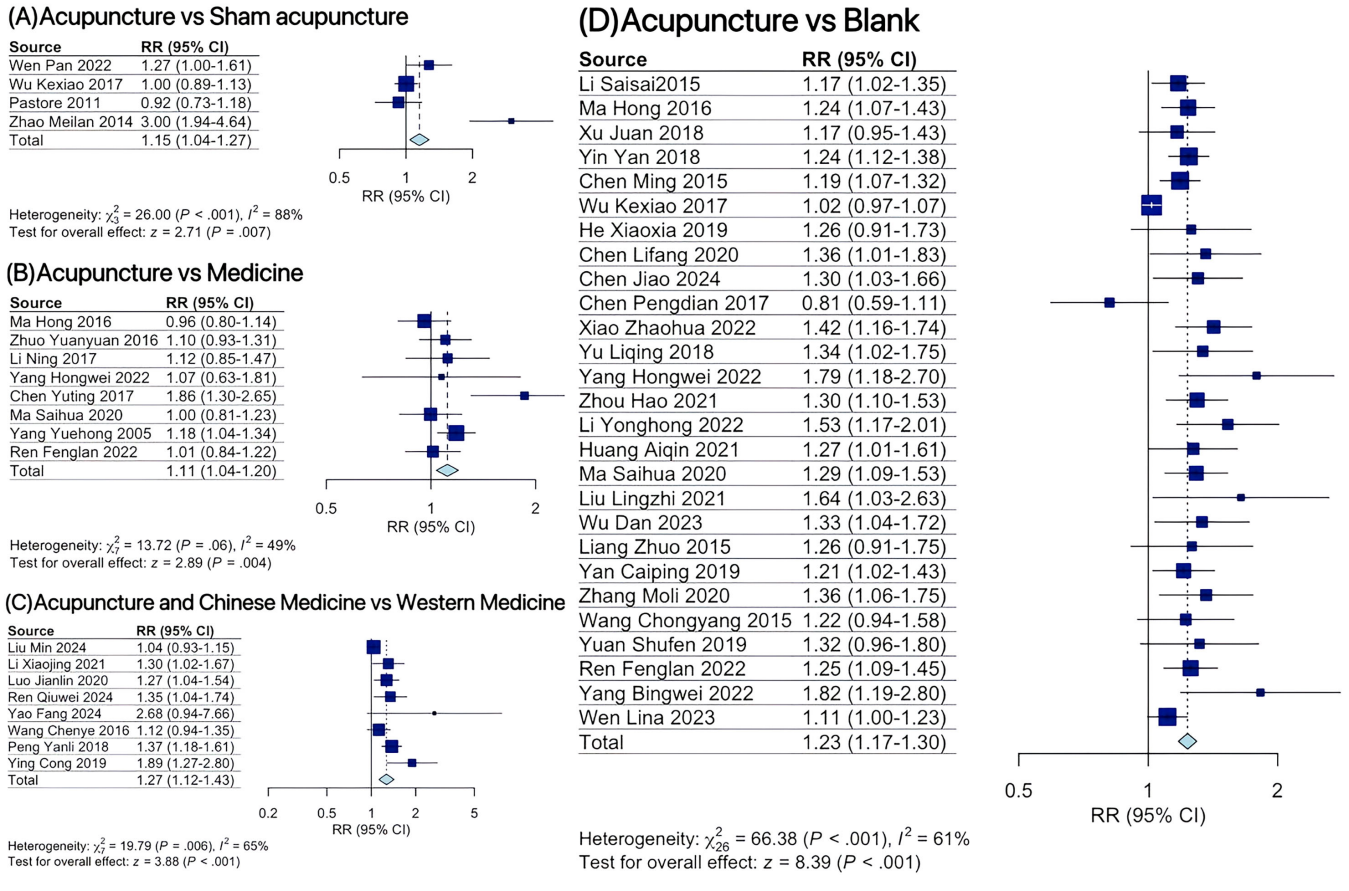
Forest plot for ovulation rate at the end of treatment. **(A)** Acupuncture versus sham acupuncture; **(B)** Acupuncture versus Medicine; **(C)** Acupuncture and Chinese Medicine versus Western Medicine; **(D)** Acupuncture versus blank.

The conventional pairwise meta-analysis comparing acupuncture and control demonstrated the following findings for ovulation rate: acupuncture was associated with a 1.15 times higher ovulation rate than sham acupuncture (RR = 1.15, 95% CI: 1.04-1.27); acupuncture showed a 1.11 times higher ovulation rate compared to medicine (RR = 1.11, 95% CI: 1.04-1.20); acupuncture combined with Chinese herbal medicine yielded a 1.27 times higher ovulation rate than Western medicine alone (RR = 1.27, 95% CI: 1.12-1.43); and acupuncture exhibited a 1.23 times higher ovulation rate compared to conventional therapy control groups (RR = 1.23, 95% CI: 1.17-1.30) (**Figure 2**). Acupuncture combined with Chinese medicine resulted in significantly greater reductions in LH levels relative to conventional Western medicine (SMD = 1.49, 95% CI: 0.73-2.25). Compared to conventional therapy control groups, acupuncture interventions demonstrated statistically significant improvements in the following outcomes: LH levels (SMD = 1.17, 95% CI: 0.71-1.62), LH/FSH ratio (SMD = 0.91, 95% CI: 0.08-1.74), T levels (SMD = 0.71, 95% CI: 0.31-1.10), and BMI (MD = 2.81, 95% CI: 1.65-3.97) (**Supplementary File 6**).

Sensitivity analysis demonstrated that the direction and statistical significance of the pooled effect estimates remained consistent upon exclusion of any single study, indicating robust findings (**Supplementary File 7**).

In most regression and subgroup analyses, covariates including mean patient age, number of acupoints, acupuncture frequency, acupuncture type, and needle retention time did not demonstrate statistically significant effects on outcomes (**Table 1**, **Supplementary File 8**). However, when compared to medicine, the high-acupoint group (RR = 1.26, 95% CI: 1.02-1.57) showed significantly greater improvement in ovulation rates among patients with PCOS than the low-acupoint group (RR = 1.02, 95% CI: 0.93-1.12; interaction *p* < 0.01). Similarly, compared to conventional therapy control groups, the high-acupoint group (RR = 1.29, 95% CI: 1.21-1.39) exhibited a more pronounced improvement in ovulation rates than the low-acupoint group (RR = 1.20, 95% CI: 1.13-1.27; interaction *p* = 0.03). These findings identify acupuncture dosage parameters, specifically the number of acupoints and acupuncture frequency, as principal contributors to heterogeneity. In comparisons of acupuncture combined with herbal medicine versus Western pharmacotherapy, high-acupoint protocols demonstrated substantial heterogeneity (I^2^ = 78%), whereas low-acupoint protocols exhibited negligible heterogeneity (I^2^ = 0%). For acupuncture versus pharmacotherapy alone, low-acupoint, high-dose, and high-frequency regimens showed minimal heterogeneity (I^2^ = 0%), while high-acupoint, low-dose, and low-frequency regimens displayed significant heterogeneity (I^2^ = 52%, 74%, and 65%, respectively). In comparisons of acupuncture versus conventional therapy control groups, moderate-dose regimens demonstrated intermediate heterogeneity (I^2^ = 68%), whereas high- and low-dose regimens were homogeneous (I^2^ = 0% for both).

**Table 1:** Subgroup analysis for ovulation ratio.

Subgroup		Number	RR(95%CI)	Interaction p value	I^2^ (p value)
Acupuncture and Chinese Medicine versus Western Medicine					
Acupuncture dose	High dose	1	1.27(1.04,1.54)	0.61	NA
	Moderate dose	4	1.19(1.03,1.38)		71(0.02)
	Low dose	3	1.61(1.13,2.28)		46(0.16)
Acupoints	High (≥13)	3	1.22(1.08,1.37)	0.95	78(0.001)
	Low (<13)	5	1.35(1.08,1.69)		0(0.46)
Frequency	High (≥4)	5	1.21(1.01,1.44)	0.78	80(0.07)
	Low (<4)	3	1.35(1.12,1.62)		49(0.1)
Retaining time	20min	3	1.59(1.09,2.31)	0.41	57(0.10)
	30min	5	1.21(1.06,1.37)		65(0.02)
Acupuncture versus Medicine					
Acupuncture dose	High dose	3	1.17(1.94,1.3)	0.27	0(0.89)
	Moderate dose	1	1.01(0.84,1.22)		NA
	Low dose	4	1.14(0.89,1.48)		74(0.01)
Acupoints	High (≥13)	4	1.26(1.02,1.57)	<0.01	52(0.1)
	Low (<13)	4	1.02(0.93,1.12)		0(0.73)
Frequency	High (≥4)	3	1.12(1,1.25)	0.42	0(0.39)
	Low (<4)	5	1.13(0.91,1.39)		65(0.02)
Age	≥28	5	1.14(0.94,1.39)	0.91	60(0.04)
	<28	3	1.09(0.94,1.26)		46(0.16)
Acupuncture versus Blank					
Acupuncture dose	High dose	5	1.22(1.14,1.3)	0.23	0(0.41)
	Moderate dose	17	1.24(1.15,1.34)		68(<0.001)
	Low dose	5	1.26(1.16,1.36)		0(0.92)
Acupoints	High (≥13)	10	1.29(1.21,1.39)	0.03	8(0.37)
	Low (<13)	17	1.2(1.13,1.27)		62(<0.001)
Frequency	High (≥4)	10	1.22(1.15,1.29)	0.94	18(0.27)
	Low (<4)	17	1.25(1.17,1.33)		69(<0.001)
Type	Manual acupuncture	20	1.23(1.18,1.29)	0.13	23(0.17)
	Electroacupuncture	7	1.22(1.08,1.37)		73(0.001)
Age	≥28	13	1.27(1.2,1.34)	0.83	13(0.31)
	<28	14	1.21(1.14,1.3)		66(<0.001)

### 3.4 NMA

The network diagram (**Supplementary File 13**) incorporated 43 studies evaluating 10 interventions. Node sizes are proportional to the number of included studies, while edge thickness corresponds to the number of trials providing direct comparisons between interventions.

**Supplementary File 15** demonstrates that, compared to sham acupuncture, both acupuncture combined with medicine (RR = 1.45, 95% CI: 1.22-1.72) and acupuncture alone (RR = 1.21, 95% CI: 1.03-1.42) significantly improved ovulation rates in patients with PCOS. In contrast, interventions involving Chinese herbal medicine alone or pharmacotherapy (e.g., letrozole) did not reach statistical significance. SUCRA values (**Supplementary File 9**) indicated that acupuncture combined with medicine had the highest cumulative ranking probability (97.8%), followed by sham acupuncture combined with medicine (80.6%), suggesting that acupuncture combined with herbal medicine may represent the optimal intervention for improving ovulation rates.

**Figure 3:**
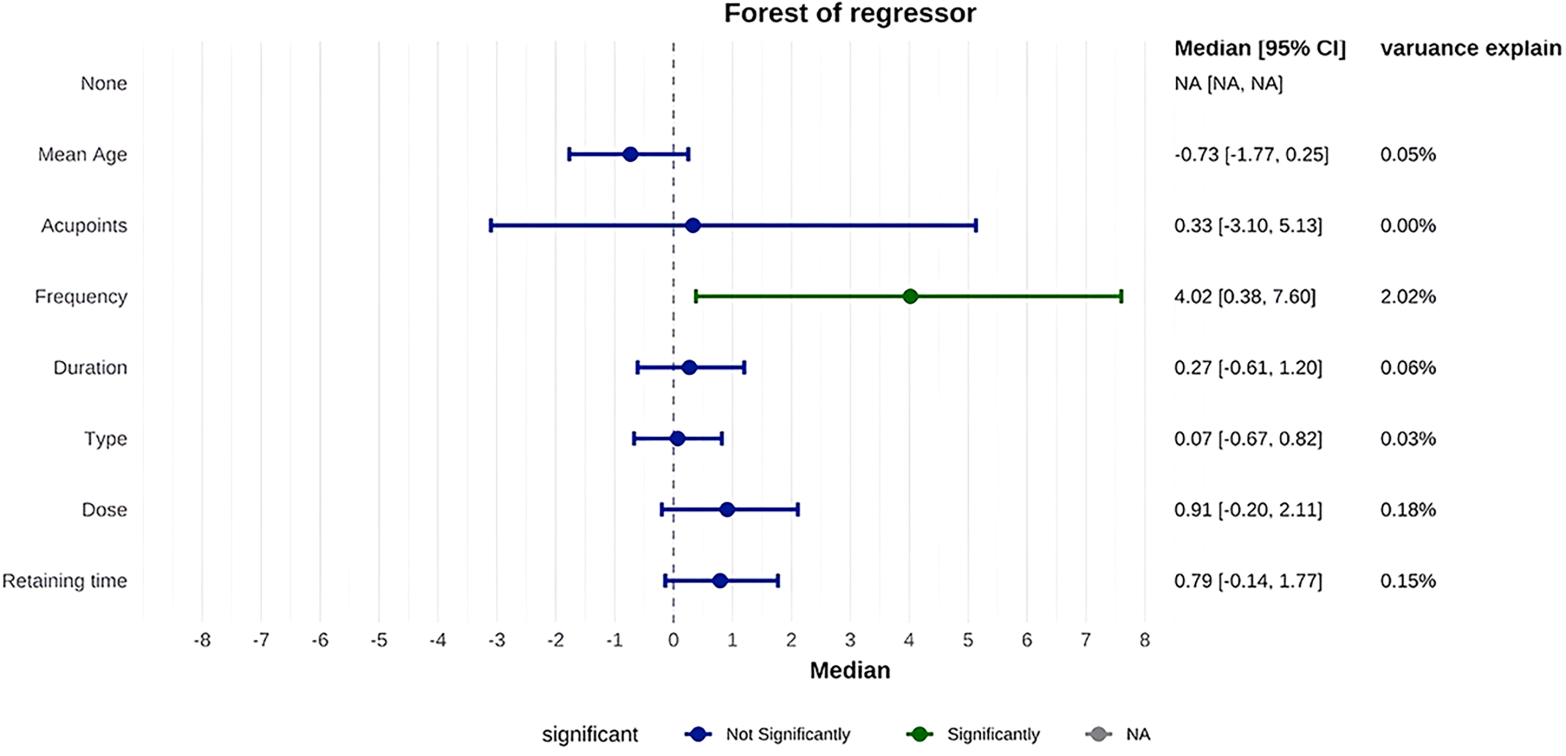
Forest plot of network meta-regression.

Network meta-regression identified acupuncture frequency and dosage as the primary sources of heterogeneity. Acupuncture frequency (β = 3.77, 95% CI: 1.52-6.21) and dosage (β = 1.41, 95% CI: 0.68-2.20) exhibited significant positive associations with ovulation rates, while mean age, number of acupoints, needle retention time, and total treatment duration showed no statistically significant effects (**Figure 3**).

### 3.5 Exploratory acupuncture dose-response

**Figure 4:**
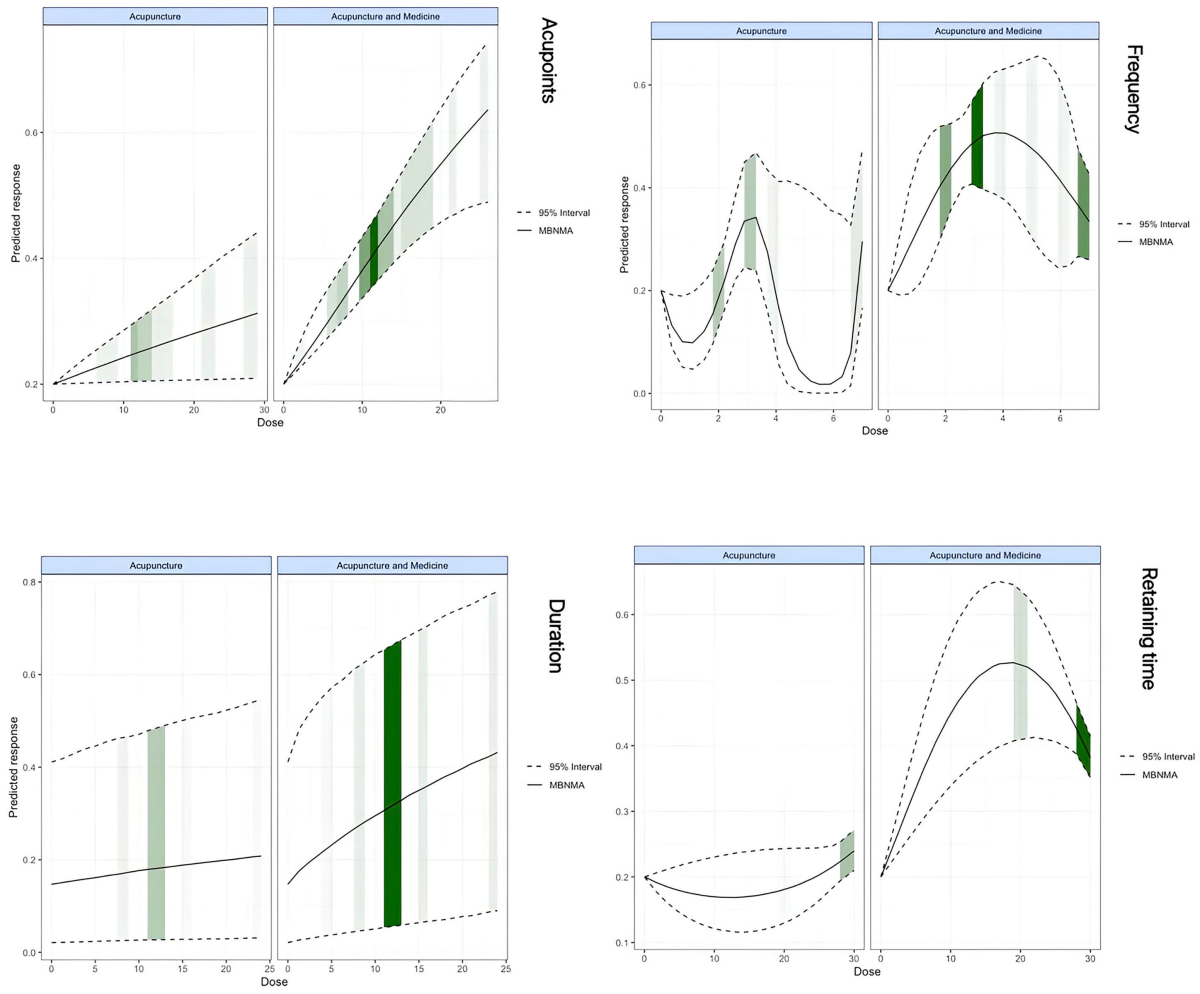
Dose-response relationships of acupuncture and acupuncture-medicine with acupoints, frequency, duration.

Our analysis identified distinct dose-response relationships for acupuncture monotherapy and acupuncture combined with medicine. Both modalities exhibited positive associations between acupoint quantity and treatment duration with ovulation rates. Notably, acupuncture frequency followed a W-shaped dose-response curve in monotherapy, whereas combination therapy demonstrated an inverted U-shaped relationship between treatment duration and efficacy. Regarding needle retention time, acupuncture exhibited a U-shaped dose-response curve, while combination therapy demonstrated an inverted U-shaped pattern. Maximal efficacy for acupoint quantity occurred at 29 acupoints in acupuncture (RR = 0.32, 95% CI: 0.21-0.45; SD = 0.06) and at 26 acupoints in combination therapy (RR = 0.64, 95% CI: 0.51-0.74; SD = 0.06). Similarly, both therapeutic regimens showed peak efficacy at 24 weeks (acupuncture: RR = 0.23, 95% CI: 0.04-0.55; SD = 0.14; combination therapy: RR = 0.42, 95% CI: 0.09-0.78; SD = 0.18). Optimal acupuncture frequency diverged between approaches, peaking at 3.3 sessions/week for acupuncture (RR = 0.33, 95% CI: 0.27-0.40; SD = 0.03) and at 4.1 sessions/week for combination therapy (RR = 0.51, 95% CI: 0.43-0.59; SD = 0.04). The optimal needle retention time was 30 minutes for acupuncture (RR = 0.24, 95% CI: 0.21-0.27; SD = 0.02) and 19 minutes for combination therapy (RR = 0.53, 95% CI: 0.41-0.65; SD = 0.06) (**Figure 4**, **Supplementary File 10**).

## 4 Discussion

### 4.1 Principal findings

To our knowledge, this is the most recent and comprehensive systematic review and meta-analysis evaluating the efficacy of acupuncture for treating PCOS while considering acupuncture type, dose, number of acupoints, frequency, and needle retention time. Our analysis included 43 RCTs involving 4,827 participants. The findings demonstrate that both acupuncture alone and acupuncture combined with pharmacotherapy significantly improve ovulation rates compared to pharmacotherapy alone or sham acupuncture, with both conventional meta-analysis and NMA confirming the superior efficacy of acupuncture-based interventions.

Specifically, acupuncture demonstrates superior efficacy in improving ovulation rates compared to sham acupuncture or pharmacotherapy alone in patients with PCOS, although it does not exhibit statistically significant effects on sex hormone levels or BMI. In contrast, compared to conventional therapy control groups, acupuncture not only enhances ovulation rates but also modulates sex hormone levels and BMI. Acupuncture combined with herbal medicine demonstrates superior efficacy to Western pharmacotherapy in improving ovulation rates and LH levels, with no significant effects on FSH levels or the LH/FSH ratio. Network meta-analysis further reveals that combining acupuncture with pharmacotherapy enhances ovulation outcomes compared to pharmacotherapy alone. Mechanistically, acupuncture may ameliorate ovulatory dysfunction by downregulating *LncMEG3*, inhibiting the PI3K/AKT/mTOR pathway, and reducing granulosa cell autophagy (74). Furthermore, acupuncture alleviates follicular arrest in PCOS rats by decreasing the overexpression of AMH and normalizing the imbalance between FSH and AMH in granulosa cells (75).

Subgroup analyses revealed that high-acupoint protocols (≥13 acupoints) demonstrated greater efficacy in improving ovulation rates compared to low-acupoint protocols (<13 acupoints) when compared against pharmacotherapy alone or conventional therapy control groups. Acupuncture dosage parameters, particularly acupoint number and stimulation frequency, emerged as primary sources of heterogeneity in pairwise meta-analyses. Network meta-regression confirmed these as key contributors, indicating a clear dose-response relationship between dosage variability and therapeutic outcomes. Exploratory analyses identified optimal efficacy with 30-minute needle retention and 29 acupoints per session, administered three times weekly for 24 weeks. In contrast, acupuncture combined with pharmacotherapy was most effective with 19-minute retention, 26 acupoints per session, and four sessions per week over the same duration. Acupuncture time-dose parameters, particularly frequency and needle retention duration, exhibit a threshold effect, highlighting the critical role of stimulus duration in modulating biochemical signaling. This effect promotes adequate neurochemical release and receptor activation, potentially influencing gene expression and cellular autophagy (20, 76).

### 4.2 Comparisons with previous studies

Over the past five years, two pairwise meta-analyses (77, 78) and one NMA (79) have examined the effects of acupuncture on PCOS. Wu Tianyu et al. (77) found that acupuncture combined with metformin significantly reduced LH levels, LH/FSH ratios, and T levels compared to metformin alone. Acupuncture combined with Chinese herbal medicine was more effective than metformin in reducing LH levels, consistent with findings comparing acupuncture to conventional therapy controls and acupuncture-herbal combinations to Western pharmacotherapy. However, the absence of sham acupuncture, herbal medicine alone, clomiphene, letrozole, and other comparators in this meta-analysis limits its utility for guiding decisions between acupuncture and alternative therapies. Unlike prior studies that focused mainly on acupoint frequency, this analysis highlights acupuncture dose, including acupoint number, stimulation frequency, and treatment duration, offering novel evidence to inform protocol standardization. Chen Xin et al. (78) reported that acupuncture combined with metformin improves ovulation rates more effectively than metformin alone, consistent with our results. Yang Lijie et al. (79) conducted an NMA identifying acupuncture combined with clomiphene as the most effective intervention for improving ovulation rates, which also corroborates our conclusions. Additionally, our exploratory dose-response analyses identified three sessions per week as the optimal acupuncture frequency for enhancing ovulation rates, aligning with Yang Lijie et al.’s findings supporting every-other-day administration.

### 4.3 Strengths and limitations

This study represents the most comprehensive evaluation to date of acupuncture’s therapeutic potential in PCOS, combining traditional pairwise meta-analysis, NMA, and exploratory dose-response modeling. Its methodological rigor and novel analytical framework offer several advantages. First, the integration of pairwise meta-analysis and NMA enables hierarchical ranking of therapeutic interventions by synthesizing direct and indirect evidence, enhancing result reliability while addressing gaps in prior reviews limited to pairwise comparisons. Second, MBNMA elucidates nonlinear relationships between key acupuncture dosage parameters (number of acupoints, frequency, duration) and ovulation outcomes, providing actionable thresholds for optimizing clinical protocols. Third, rigorous subgroup and meta-regression analyses identified acupuncture dosage as a critical source of heterogeneity, resolving inconsistencies in prior studies by demonstrating superior ovulation rates with high-acupoint protocols compared to low-acupoint regimens. Finally, the application of the CINeMA framework enhanced transparency in assessing evidence certainty.

However, this study has several limitations. First, although the exploratory dose-response modeling is innovative, its reliance on Bayesian assumptions may oversimplify the complex biological interactions between acupuncture parameters and physiological responses. Second, substantial heterogeneity across trials reduced the certainty of evidence to low or very low for many comparisons. Third, the acupuncture dosage scoring tool, a non-weighted composite of acupoint quantity, deqi response, treatment frequency, and duration, is based on Chinese clinical standards, which may limit its applicability to studies conducted in other regions. Fourth, comparisons involving acupuncture combined with herbal medicine versus Western medicine complicate the attribution of efficacy solely to acupuncture. While these results support the benefits of integrated Traditional Chinese Medicine approaches, they reflect synergistic or additive effects rather than acupuncture’s isolated impact, potentially overestimating its standalone efficacy. Lastly, the limited number of included trials precluded funnel plot analysis, restricting the ability to assess publication bias comprehensively.

### 4.4 Perspectives

Current research has rarely focused on optimizing acupuncture protocols and parameter selection, including acupuncture type and dosage. Although our subgroup analyses revealed no differences between electroacupuncture and manual acupuncture based on interaction *p*-values, acupuncture dose and its key parameters demonstrated significant impacts on treatment outcomes. Therefore, future RCTs should place greater emphasis on standardizing acupuncture dosage. Additionally, retention time is a critical yet long-overlooked parameter. While most current RCTs default to a 30-minute retention period, our subgroup analyses demonstrated higher effect sizes for ovulation rate improvement in the 20-minute retention group. Future studies should design head-to-head RCTs (e.g., 20 vs. 30 vs. 45 minutes) incorporating functional near-infrared spectroscopy (fNIRS) to dynamically monitor ovarian hemodynamic changes and define optimal retention windows. Concurrently, the interaction between retention time and other parameters warrants exploration. For example, it remains unclear whether high acupoint counts combined with shorter retention times yield synergistic effects or whether specific acupoints (e.g., Zigong [CV4], Sanyinjiao [SP6]) require differential retention durations for maximal efficacy. Furthermore, clinical RCTs should prioritize rigorous blinding implementation. Although the procedural nature of acupuncture poses challenges for practitioner blinding, blinding of participants and outcome assessors should be consistently enforced to minimize bias and enhance methodological rigor.

## 5 Conclusion

Acupuncture serves as an effective non-pharmacological intervention for addressing PCOS-related ovulatory dysfunction. Furthermore, this study identifies acupuncture dosage and parameter selection as critical determinants of therapeutic efficacy and predicts their optimal thresholds. Future research should prioritize high-quality, dose-finding RCTs to validate these dose-response thresholds and refine personalized therapeutic algorithms.

## Data Availability

The original contributions presented in the study are included in the article/**Supplementary Material**. Further inquiries can be directed to the corresponding author.
