# Supplementary material for: Dose-response of acupuncture on ovulation rates in polycystic ovary syndrome: a meta-analysis and exploratory dose-response analysis
Source: Front Endocrinol (Lausanne). 2025 Aug 28;16:1610338. doi: 10.3389/fendo.2025.1610338 (PMC12422914; doi:10.3389/fendo.2025.1610338)
Supplement: Supplementary file 1 [file DataSheet1.docx]

CONTENTS:

[1 Supplemental file 1. Search strategies: 2](#_Toc194442448)

[2 Supplemental file 2. Definitions used to classify trials according to components of methodological quality: 8](#_Toc194442449)

[3 Supplemental file 3. Funnel plot 10](#_Toc194442450)

[4 Supplemental file 4. Characteristics of the included studies (n = 43) 15](#_Toc194442451)

[6 Supplemental file 6. Forest plot of Pairwise Meta-Analysis: 27](#_Toc194442452)

[7 Supplemental file 7. Forest plot of sensitivity analysis： 32](#_Toc194442453)

[8 Supplemental file 8. Subgroup analysis of Pairwise Meta-Analysis 37](#_Toc194442454)

[9 Supplemental file 9. Histogram plot of the network meta-analyses 47](#_Toc194442455)

[10 Supplemental file 10. Exploratory acupuncture dose-response 50](#_Toc194442456)

[11 Supplemental file 11. GRADE Framework 58](#_Toc194442457)

[12 Supplemental file 12. Summary of findings 59](#_Toc194442458)

[13 Supplemental file 13. Network diagram of the network meta-analysis 65](#_Toc194442459)

[14 Supplemental file 14. Forest plot of node splitting analysis 67](#_Toc194442460)

[15 Supplemental file 15. Forest plot of network meta-analysis compared with sham acupuncture 72](#_Toc194442461)

1. **Supplemental file 1. Search strategies:**

**PubMed:**

| Search number | Query | Results |
| --- | --- | --- |
| 1 | "Polycystic Ovary Syndrome"[Mesh] | 19,557 |
| 2 | (((((((((((((Ovary Syndrome, Polycystic[Title/Abstract]) OR (Syndrome, Polycystic Ovary[Title/Abstract])) OR (Polycystic Ovarian Syndrome[Title/Abstract])) OR (Ovarian Syndrome, Polycystic[Title/Abstract])) OR (Polycystic Ovary Syndrome 1[Title/Abstract])) OR (Sclerocystic Ovarian Degeneration[Title/Abstract])) OR (Ovarian Degeneration, Sclerocystic[Title/Abstract])) OR (Sclerocystic Ovary Syndrome[Title/Abstract])) OR (Stein-Leventhal Syndrome[Title/Abstract])) OR (Stein Leventhal Syndrome[Title/Abstract])) OR (Syndrome, Stein-Leventhal[Title/Abstract])) OR (Sclerocystic Ovaries[Title/Abstract])) OR (Ovary, Sclerocystic[Title/Abstract])) OR (Sclerocystic Ovary[Title/Abstract]) | 5,208 |
| 3 | #1 OR #2 | 21,387 |
| 4 | "Acupuncture"[Mesh] | 2,103 |
| 5 | Pharmacopuncture[Title/Abstract] | 296 |
| 6 | #3 OR #4 | 2,376 |
| 7 | "Acupuncture Therapy"[Mesh] | 31,241 |
| 8 | ((((((((Acupuncture Treatment[Title/Abstract]) OR (Acupuncture Treatments[Title/Abstract])) OR (Therapy, Acupuncture[Title/Abstract])) OR (Pharmacoacupuncture Treatment[Title/Abstract])) OR (Treatment, Pharmacoacupuncture[Title/Abstract])) OR (Pharmacoacupuncture Therapy[Title/Abstract])) OR (Therapy, Pharmacoacupuncture[Title/Abstract])) OR (Acupotomy[Title/Abstract])) OR (Acupotomies[Title/Abstract]) | 4,421 |
| 9 | #7 OR #8 | 32,415 |
| 10 | Electroacupuncture[mesh] | 5,451 |
| 11 | "Acupuncture Points"[Mesh] | 8,563 |
| 12 | ((((Acupuncture Point[Title/Abstract]) OR (Point, Acupuncture[Title/Abstract])) OR (Points, Acupuncture[Title/Abstract])) OR (Acupoints[Title/Abstract])) OR (Acupoint[Title/Abstract]) | 8,537 |
| 13 | #10 OR #11 OR #12 | 12,607 |
| 14 | #6 OR #9 OR #14 | 35,038 |
| 15 | (clinical[tiab]) AND (trial[tiab]) | 414,706 |
| 16 | (((((clinical trials as topic[mesh]) OR (clinical trial[pt])) OR (random*[tiab])) OR (random allocation[mesh])) OR (therapeutic use[sh])) OR ((clinical[tiab]) AND (trial[tiab])) | 6,693,904 |
| 17 | #2 AND #14 AND #16 | 147 |

**Web of Science:**

| # | Search Query | Results |
| --- | --- | --- |
| 1 | TS=(Acupuncture OR Electroacupuncture OR Pharmacopuncture) and Preprint Citation Index (Exclude – Database) | 54692 |
| 2 | TS=(Acupuncture Therapy OR Acupuncture Treatment OR Acupuncture  Treatments OR Treatment, Acupuncture OR Therapy, Acupuncture OR  Pharmacoacupuncture Treatment OR Treatment, Pharmacoacupuncture OR  Pharmacoacupuncture Therapy OR Therapy, Pharmacoacupuncture) and Preprint Citation Index (Exclude – Database) | 44283 |
| 3 | TS=(Acupuncture Points OR Acupuncture Point OR Point, Acupuncture OR  Points, Acupuncture OR Acupoints OR Acupoint) and Preprint Citation Index (Exclude – Database) | 20141 |
| 4 | #3 OR #2 OR #1 | 55636 |
| 5 | TS=(Polycystic Ovary Syndrome OR Ovary Syndrome, Polycystic OR Syndrome, Polycystic Ovary OR Polycystic Ovarian Syndrome OR Ovarian Syndrome, Polycystic OR Polycystic Ovary Syndrome 1 OR Sclerocystic Ovarian Degeneration OR Ovarian Degeneration, Sclerocystic OR Sclerocystic Ovary Syndrome OR Stein-Leventhal Syndrome OR Stein Leventhal Syndrome OR Syndrome, Stein-Leventhal OR Sclerocystic Ovaries OR Ovary, Sclerocystic OR Sclerocystic Ovary) and Preprint Citation Index (Exclude – Database) | 37193 |
| 6 | #5 AND #4 | 401 |
| 7 | TS=(Randomized Controlled Trials as Topic OR Clinical Trials, Randomized OR Trials, Randomized Clinical OR Controlled Clinical Trials, Randomized) and Preprint Citation Index (Exclude – Database) | 745012 |
| 8 | TS=(Randomized Controlled Trial) and Preprint Citation Index (Exclude – Database) | 796898 |
| 9 | #8 OR #7 | 962774 |
| 10 | #9 AND #6 | 143 |

**Embase:**

| No. | Query | Results |
| --- | --- | --- |
| #19 | #3 AND #12 AND #18 | 278 |
| #18 | #13 OR #14 OR #15 OR #16 OR #17 | 3762022 |
| #17 | 'trial':ti | 451712 |
| #16 | 'randomized controlled trial'/exp OR 'randomized controlled trial' OR 'randomized controlled trial':ab,ti | 1165641 |
| #15 | 'controlled clinical trial'/exp OR 'controlled clinical trial' OR 'controlled clinical trial':ab,ti | 1064024 |
| #14 | 'clinical trial'/exp OR 'clinical trial' OR 'clinical trial':ti | 2427741 |
| #13 | random*:ab | 2096609 |
| #12 | #4 OR #5 OR #6 OR #7 OR #8 OR #9 OR #10 OR #11 | 81852 |
| #11 | deqi:ab,ti | 313 |
| #10 | meridian$:ab,ti | 8701 |
| #9 | acupoint$:ab,ti | 9476 |
| #8 | electroacupuncture:ab,ti | 9179 |
| #7 | acupuncture:ab,ti | 41542 |
| #6 | 'electroacupuncture'/exp OR 'electroacupuncture' OR 'meridians'/exp OR 'meridians' | 15950 |
| #5 | 'acupuncture therapy'/exp OR 'acupuncture therapy' OR 'acupuncture treatment' OR 'acupuncture treatments' OR 'treatment, acupuncture' OR 'therapy, acupuncture' | 62699 |
| #4 | 'acupuncture'/exp OR 'acupuncture' OR 'acupuncture points'/exp OR 'acupuncture points' OR 'points, acupuncture' | 75751 |
| #3 | #1 OR #2 | 43656 |
| #2 | 'cystic ovary':ti,ab OR 'micro polycystic ovary':ti,ab OR 'multiple follicle cyst':ti,ab OR 'ovary polycystic syndrome':ti,ab OR 'ovary, micro polycystic':ti,ab OR 'ovary, polycystic':ti,ab OR 'polycystic ovarian disease':ti,ab OR 'polycystic ovary':ti,ab OR 'polycystic ovary disease':ti,ab OR 'polycystic ovary syndrome':ti,ab OR 'stein cohen leventhal syndrome':ti,ab OR 'stein leventhal disease':ti,ab OR 'stein leventhal syndrome':ti,ab OR 'syndrome stein leventhal':ti,ab OR 'ovary polycystic disease':ti,ab | 28004 |
| #1 | 'ovary polycystic disease'/exp | 41794 |

**Cochrane Library:**

| ID | Search | Hits |
| --- | --- | --- |
| #1 | Polycystic Ovary Syndrome | 4859 |
| #2 | (Polycystic Ovary Syndrome):ab,ti,kw OR(Ovary Syndrome, Polycystic):ab,ti,kw OR(Syndrome, Polycystic Ovary):ab,ti,kw OR(Polycystic Ovarian Syndrome):ab,ti,kw OR(Ovarian Syndrome, Polycystic):ab,ti,kw OR(Polycystic Ovary Syndrome 1):ab,ti,kw OR(Sclerocystic Ovarian Degeneration):ab,ti,kw OR(Ovarian Degeneration, Sclerocystic):ab,ti,kw OR(Sclerocystic Ovary Syndrome):ab,ti,kw OR(Stein-Leventhal Syndrome):ab,ti,kw OR(Stein Leventhal Syndrome):ab,ti,kw OR(Syndrome, Stein-Leventhal):ab,ti,kw OR(Sclerocystic Ovaries):ab,ti,kw OR(Ovary, Sclerocystic):ab,ti,kw | 5220 |
| #3 | #1 OR #2 | 5335 |
| #4 | MESH DESCRIPTOR Acupuncture EXPLODE ALL TREES | 199 |
| #5 | MESH DESCRIPTOR Acupuncture Therapy EXPLODE ALL TREES | 197 |
| #6 | MESH DESCRIPTOR Electroacupuncture EXPLODE ALL TREES | 46 |
| #7 | MESH DESCRIPTOR Acupuncture Points EXPLODE ALL TREES | 169 |
| #8 | (Acupuncture OR Acupoint* OR Meridian*):ab,ti,kw | 24242 |
| #9 | (Electroacupuncture OR Electro-acupuncture):ab,ti,kw | 3933 |
| #10 | (Acupunctur* OR Needling OR Acup* point*):ab,ti,kw | 24358 |
| #11 | #4 OR #5 OR #6 OR #7 OR #8 OR #9 OR #10 | 27994 |
| #12 | #3 AND #11 | 240 |

**Ovid Medline:**

| 1 | Polycystic Ovary Syndrome/ | 19703 |
| --- | --- | --- |
| 2 | (Ovary Syndrome, Polycystic or Syndrome, Polycystic Ovary or Polycystic Ovarian Syndrome or Ovarian Syndrome, Polycystic or Polycystic Ovary Syndrome 1 or Sclerocystic Ovarian Degeneration or Ovarian Degeneration, Sclerocystic or Sclerocystic Ovary Syndrome or Stein-Leventhal Syndrome or Stein Leventhal Syndrome or Syndrome, Stein-Leventhal or Sclerocystic Ovaries or Ovary, Sclerocystic or Sclerocystic Ovary).ab,ti,kw. | 5153 |
| 3 | 1 or 2 | 21513 |
| 4 | (Acupuncture or Acupuncture Therapy or Acupuncture, Ear or Acupuncture Points).mp. [mp=title, book title, abstract, original title, name of substance word, subject heading word, floating sub-heading word, keyword heading word, organism supplementary concept word, protocol supplementary concept word, rare disease supplementary concept word, unique identifier, synonyms, population supplementary concept word, anatomy supplementary concept word] | 37434 |
| 5 | (PharmacopunctureAcupuncture Treatment or Acupuncture Treatments or Treatment, Acupuncture or Therapy, Acupuncture or Pharmacoacupuncture Treatment or Treatment, Pharmacoacupuncture or Pharmacoacupuncture Therapy or Therapy, Pharmacoacupuncture or Acupotomy or AcupotomiesAcupunctures, Ear or Ear Acupunctures or Acupuncture, Auricular or Acupunctures, Auricular or Auricular Acupunctures or Auricular Acupuncture or Ear Acupuncture Acupuncture Point or Point, Acupuncture or Points, Acupuncture or Acupoints or Acupoint).ab,ti,kw. | 9540 |
| 6 | 4 or 5 | 38796 |
| 7 | (trial or random or randomized controlled trialOR controlled clinical or clinical trial).ab,ti,kw. | 1236653 |
| 8 | 3 and 6 and 7 | 74 |

**VIP:**

| #1 | (((((题名或关键词=针刺 OR 题名或关键词=毫针) OR 题名或关键词=电针) OR 题名或关键词=手针) OR 题名或关键词=安慰针) OR 题名或关键词=针灸) | 171822 |
| --- | --- | --- |
| #2 | ((((((((题名或关键词=多囊卵巢综合征 OR 题名或关键词=polycystic ovarian syndrome) OR 题名或关键词=polycystic ovary syndrome) OR 题名或关键词=women with polycystic ovary syndrome) OR 题名或关键词=多囊性卵巢综合症) OR 题名或关键词=多囊性卵巢综合征) OR 题名或关键词=多囊卵巢综合证) OR 题名或关键词=多囊卵巢综合症) OR 题名或关键词=多囊性卵巢) | 21631 |
| #3 | ((((((((题名或关键词=随机对照试验 OR 题名或关键词=randomized clinical trials) OR 题名或关键词=randomized controlled clinical trial) OR 题名或关键词=randomized controlled trial) OR 题名或关键词=randomized controlled trials) OR 题名或关键词=randomized experiment) OR 题名或关键词=rct) OR 题名或关键词=随机对照实验) OR 题名或关键词=随机对照研究) | 26402 |
| #4 | #1 AND #2 AND #3 | 31 |

**CNKI:**

| #1 | 篇关摘=（针刺 + 针灸 + 电针 + 毫针 + 安慰针） | NA |
| --- | --- | --- |
| #2 | 篇关摘=（多囊卵巢综合征 + 多囊卵巢 + polycystic ovarian syndrome + polycystic ovary syndrome + 多囊卵巢综合症 + 多囊卵巢综合证） | NA |
| #3 | 篇关摘=（随机对照试验 + '随机对照试验(rct)' + 随机对照试验研究 + '随机对照试验(rcts)' + 随机对照试验法 + 随机对照试验rcts + RCT + 随机 + 试验） | NA |
| #4 | #1 AND #2 AND #3 | 405 |

**WanFang:**

| #1 | 主题=（针刺 OR 针灸 OR 电针 OR 毫针 OR 安慰针） | NA |
| --- | --- | --- |
| #2 | 主题=（多囊卵巢综合症 OR 多囊卵巢综合征 OR 多囊卵巢综合证 OR 多囊卵巢 OR polycystic ovarian syndrome） | NA |
| #3 | 主题=（随机对照试验 OR 随机 OR 试验 OR RCT） | NA |
| #4 | #1 AND #2 AND #3 AND #4 | 550 |

**CBM：**

| 序号 | 检索表达式 | 命中文献数 |
| --- | --- | --- |
| 1) | "针刺"[常用字段:智能] OR( "针灸"[常用字段:智能] OR "电针"[常用字段:智能] OR "毫针"[常用字段:智能] OR "手针"[常用字段:智能] OR "安慰针"[常用字段:智能]) | 279921 |
| 2) | "多囊卵巢综合征"[常用字段:智能] OR( "多囊卵巢综合症"[常用字段:智能] OR "多囊卵巢综合证"[常用字段:智能] OR "多囊卵巢"[常用字段:智能]) | 21034 |
| 3) | "随机对照试验"[常用字段:智能] OR( "随机"[常用字段:智能] OR "试验"[常用字段:智能] OR "RCT"[常用字段:智能]) | 2501111 |
| 4) | (#3) AND (#2) AND (#1) | 654 |

1. **Supplemental file 2.** **Definitions used to classify trials according to components of methodological quality:**
   1. **Randomization Process:**

Trials were classified as having a "low risk" for random sequence generation if appropriate randomization methods were applied, including the use of random number tables, computer-generated randomization, coin tossing, dice rolling, or lottery-based allocation. Trials utilizing any of the following methods were assigned a "high risk": sequences generated by parity of birth dates, clinician judgment-based allocation, participant preference-driven allocation, intervention availability-based assignment, or any method deemed inadequate per Cochrane guidelines. Trials with insufficiently documented randomization procedures were categorized as "some concerns"

- 1. **Deviations From Interventions**

Given the inherent limitations of blinding practitioners in acupuncture trials, studies were categorized as "low risk" if participant blinding was ensured, protocol deviations were unlikely to occur, and interventions were administered as planned without systematic crossover or contamination. Conversely, trials were classified as "high risk" when no blinding was implemented, and outcomes were potentially influenced by the absence of blinding. In cases where blinding was not feasible, but outcome assessments relied on objective measures, the risk of bias was rated as "some concerns".

- 1. **Missing Outcome Data**

Trials were classified as "low risk" of bias if they met all of the following criteria: A low proportion of missing data (typically <10%);Missingness unrelated to the intervention or outcomes (e.g., random loss to follow-up);Application of validated methods to address missing data (e.g., intention-to-treat [ITT] analysis, multiple imputation).The "some concerns" classification was assigned when any of the following applied: Moderate missing data (10%-20%) with unclear mechanisms underlying missingness; Inadequate handling of missing data (e.g., complete case analysis);Insufficient evidence to determine whether missingness was related to the intervention. Trials were deemed "high risk" if they exhibited: High missing data (>20%); Missingness directly attributable to the intervention or outcomes (e.g., attrition due to adverse effects); Failure to address missing data or selective exclusion of participants.

- 1. **Measurement Of Outcome**

Trials were classified as "low risk" of bias if outcomes were objectively measured with both participants and outcome assessors blinded to group allocation. A "some concerns" rating was assigned to trials utilizing subjective outcome measures where blinding procedures were inadequately implemented, or methodological details of assessment were insufficiently described. Trials were deemed "high risk" if subjective outcomes were evaluated without blinding, with explicit awareness of group allocation among assessors or participants.

- 1. **Selection of Reported Result**

Trials were classified as "low risk" of bias if a publicly accessible study protocol (e.g., pre-registered on clinical trial registries) was available and all predefined outcomes were comprehensively reported. "Some concerns" rating was assigned when the study protocol could not be retrieved, or secondary outcomes/subgroup analyses were inadequately documented. Trials were deemed "high risk" if predefined primary outcomes were omitted, selectively reported (e.g., overemphasis on positive results with concealment of negative findings), or subjected to outcome switching.

- 1. **Overall**

Trials were assigned one of three methodological rigor classifications based on domain-specific bias evaluations: ‘Low risk': All methodological domains were rated as low risk of bias. ‘Some concerns': At least one domain exhibited potential limitations not severe enough to warrant high-risk designation. ‘High risk': One or more domains demonstrated substantial bias likely to compromise validity.

1. **Supplemental file 3. Funnel plot**

**FIGURE3. 1.** Funnel plot for assessment of publication bias on ovulation rate in acupuncture versus blank groups at the end of treatment.


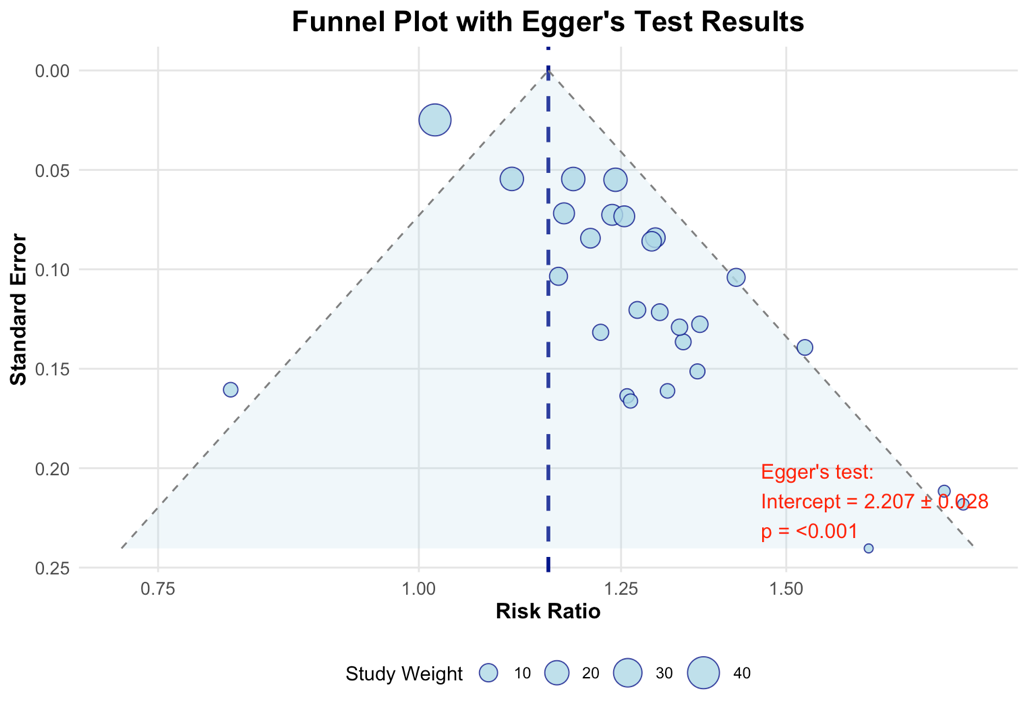


FIGURE3. 2 Funnel plot for assessment of publication bias on FSH in acupuncture versus blank groups at the end of treatment.


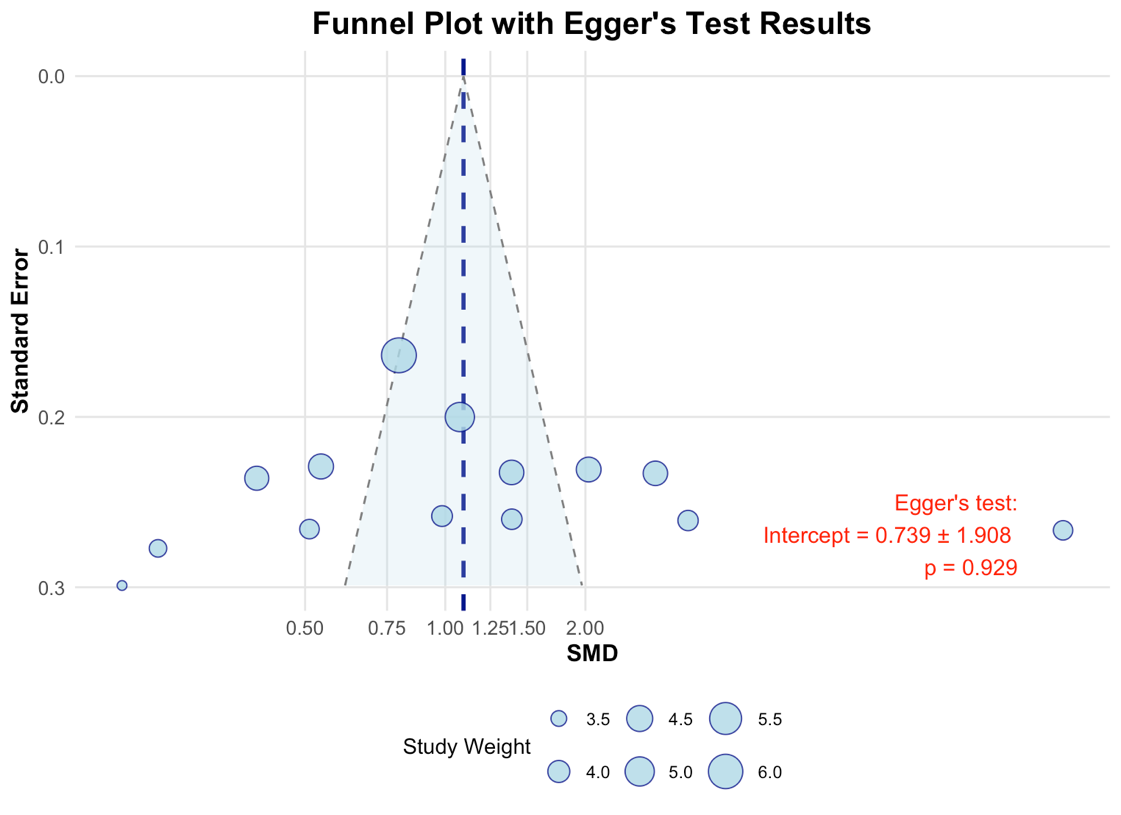


FIGURE3. 3 Funnel plot for assessment of publication bias on LH in acupuncture versus blank groups at the end of treatment.


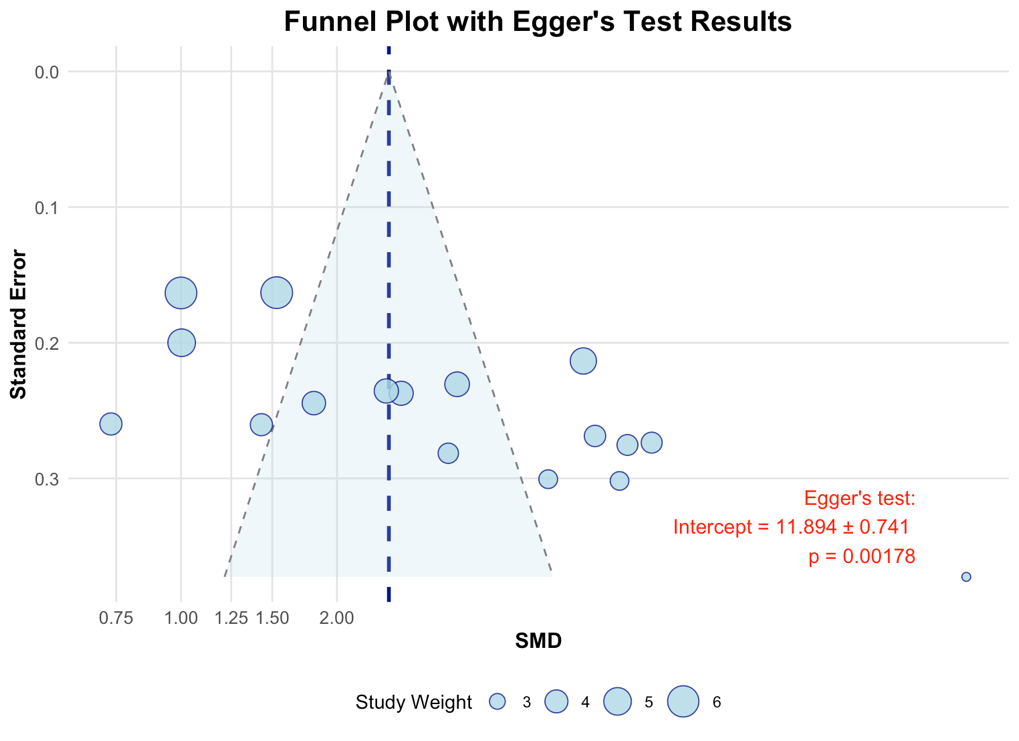


**FIGURE3. 4 Funnel plot for assessment of publication bias on T in acupuncture versus blank groups at the end of treatment.**


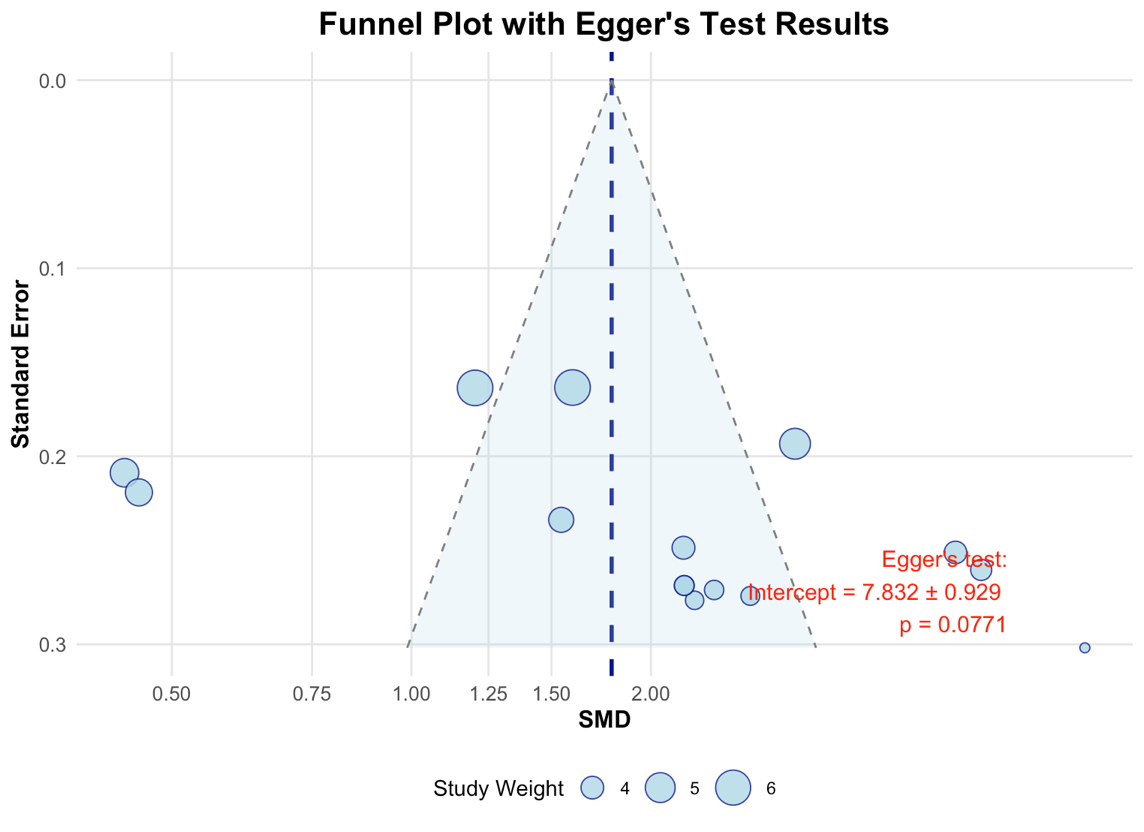


**FIGURE3. 5 Funnel plot for assessment of publication bias on FSH in NMA**


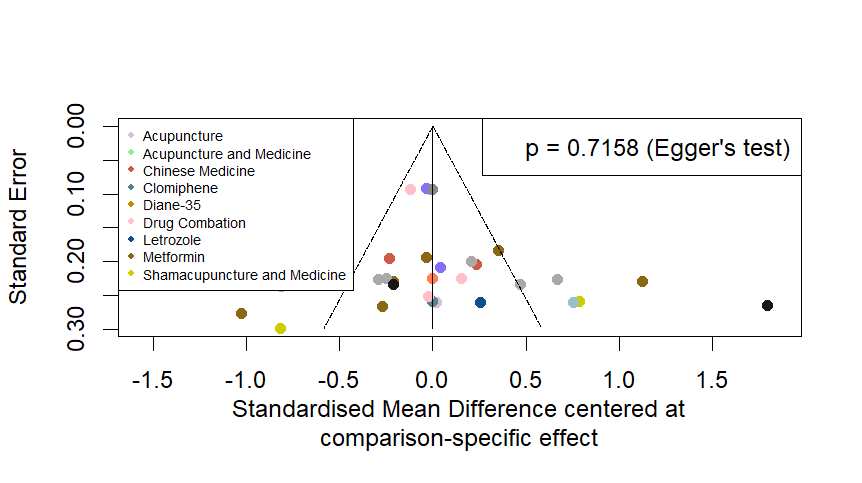


**FIGURE3. 6 Funnel plot for assessment of publication bias on BMI in NMA.**


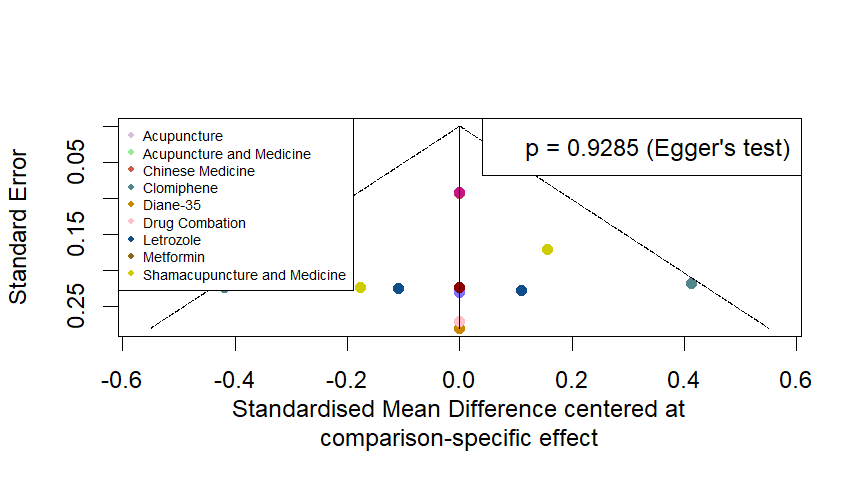


**FIGURE3. 7. Funnel plot for assessment of publication bias on LH in NMA**


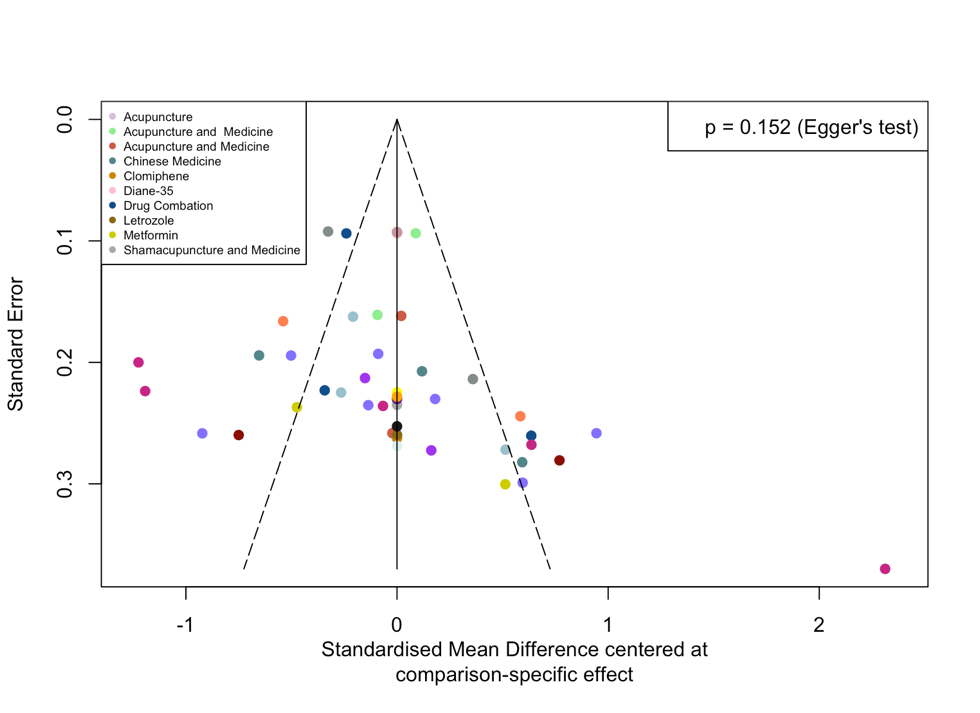


**FIGURE3. 8 Funnel plot for assessment of publication bias on LH：FSH in NMA**


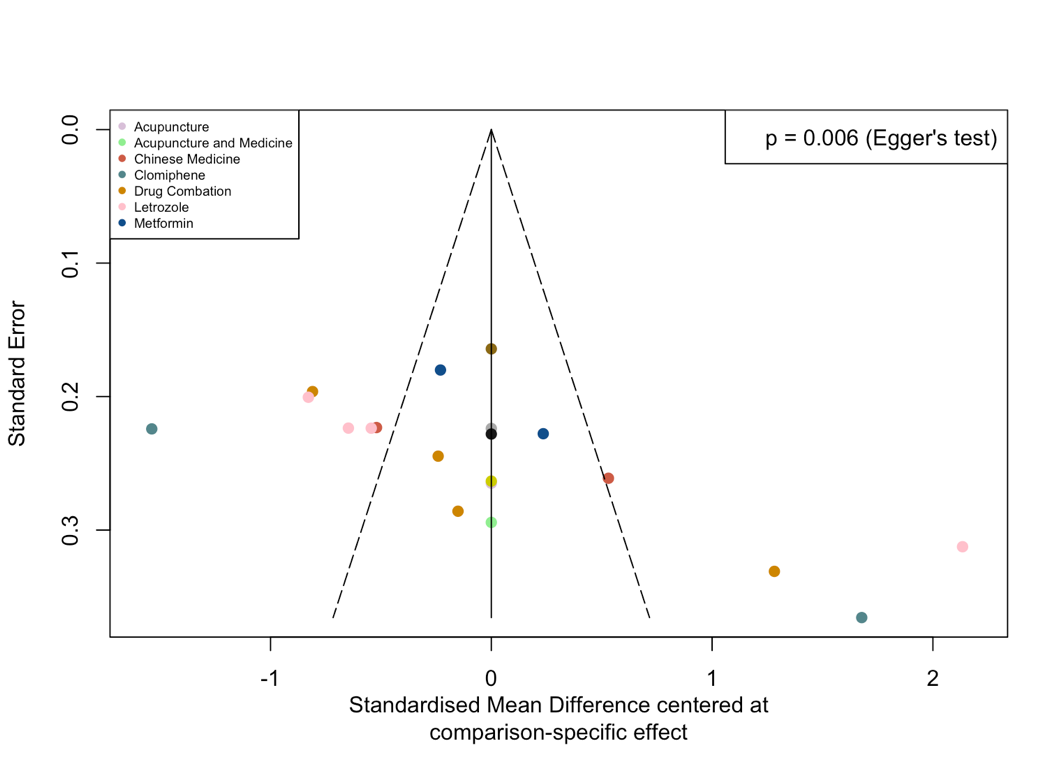


**FIGURE3. 9 Funnel plot for assessment of publication bias on T in NMA**


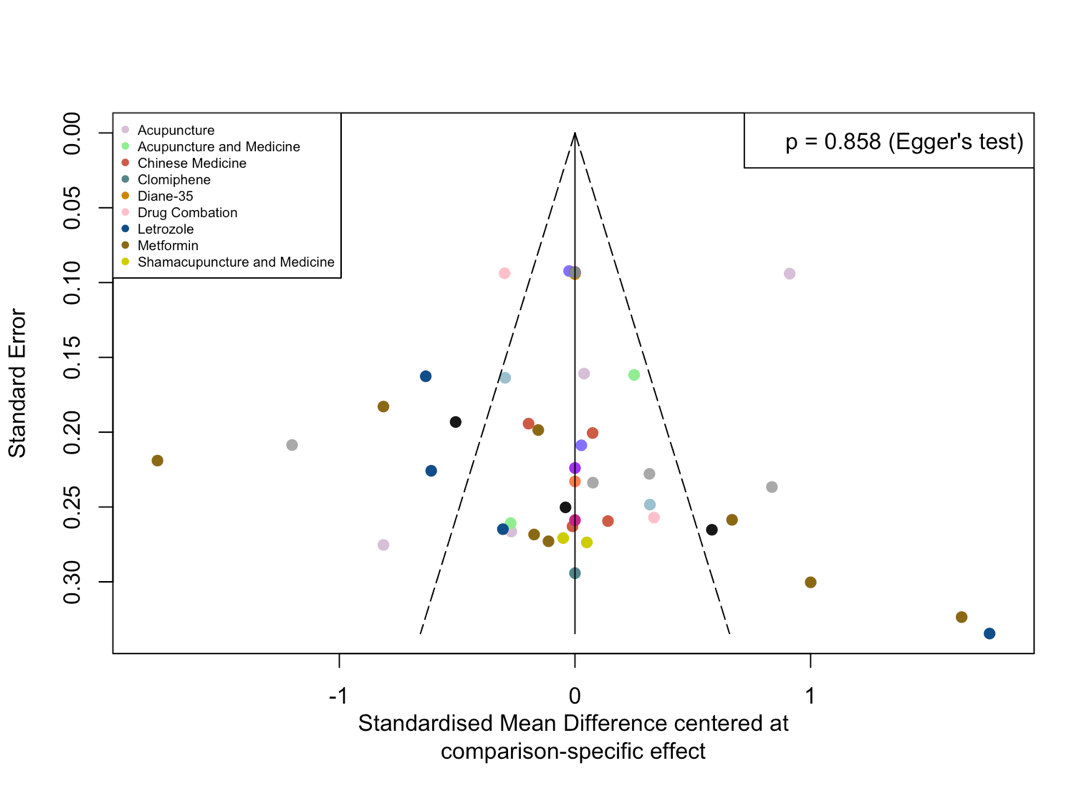


1. **Supplemental file 4. Characteristics of the included studies (n = 43)**

| Trial | Type | Number | Mean Age | Course | Intervention | Outcomes |
| --- | --- | --- | --- | --- | --- | --- |
| Li Saisai 2015(1) | RCT | 75 | 24.10 | 6 month | Acupuncture and Metformin | FSH,LH,LH/FSH,T,BMI,Ovulation Rate |
| Li Saisai 2015 | RCT | 75 | 25.10 | 6 month | Metformin | FSH,LH,LH/FSH,T,BMI,Ovulation Rate |
| Ma Hong 2016(2) | RCT | 29 | 23.00 | 3 month | Acupuncture and Clomiphene | T,Ovulation Rate |
| Ma Hong 2016 | RCT | 30 | 24.00 | 3 month | Acupuncture | T,Ovulation Rate |
| Ma Hong 2016 | RCT | 28 | 25.00 | 3 month | Clomiphene | T,Ovulation Rate |
| Xu Juan 2018(3) | RCT | 30 | 25.70 | 2 menstryal cycle | Acupuncture and Diane-35 | FSH,LH,T,BMI,Ovulation Rate |
| Xu Juan 2018 | RCT | 30 | 25.80 | 2 menstryal cycle | Diane-35 | FSH,LH,T,BMI,Ovulation Rate |
| Yin Yan 2018(4) | RCT | 40 | 28.00 | 6 month | Diane-35 and Letrozole | FSH,LH,LH/FSH,T,BMI,Ovulation Rate，AMH,IHNB |
| Yin Yan 2018 | RCT | 40 | 28.00 | 6 month | Chinese Medicine | FSH,LH,LH/FSH,T,BMI,Ovulation Rate，AMH,IHNB |
| Yin Yan 2018 | RCT | 40 | 28.00 | 6 month | Electroacupuncture、Chinese Medicine、Diane-35 and Letrozole | FSH,LH,LH/FSH,T,BMI,Ovulation Rate，AMH,IHNB |
| Zhuo Yuanyuan 2016(5) | RCT | 50 | 29.00 | 3 menstrual cycle | Acupuncture | FSH,LH,T,Ovulation Rate |
| Zhuo Yuanyuan 2016 | RCT | 50 | 28.00 | 3 menstrual cycle | Clomiphene | FSH,LH,T,Ovulation Rate |
| Chen Ming 2015(6) | RCT | 50 | 24.00 | 3 menstrual cycle | Acupuncture and Clomiphene and Diane-35 | FSH,LH,LH/FSH,T,Ovulation Rate |
| Chen Ming 2015 | RCT | 50 | 26.00 | 3 menstrual cycle | Clomiphene and Metformin and Diane-35 | FSH,LH,LH/FSH,T,Ovulation Rate |
| Wen Pan 2022(7) | RCT | 31 | 30.80 | 3 menstrual cycle | Acupuncture | FSH,LH,LH/FSH,T,HOMA-IR,Ovulation Rate |
| Wen Pan 2022 | RCT | 33 | 29.70 | 3 menstrual cycle | Shamacupuncture | FSH,LH,LH/FSH,T,HOMA-IR,Ovulation Rate |
| Wu Xiaoke 2017(8) | RCT | 235 | 28.20 | 16 week | Electroacupuncture and Clomiphene | FSH,LH,T,SAS,SDS,BMI,Ovulation Rate |
| Wu Xiaoke 2017 | RCT | 235 | 27.80 | 16 week | Shamacupuncture and Clomiphene | FSH,LH,T,SAS,SDS,BMI,Ovulation Rate |
| Wu Xiaoke 2017 | RCT | 223 | 27.80 | 16 week | Electroacupuncture | FSH,LH,T,SAS,SDS,BMI,Ovulation Rate |
| Wu Xiaoke 2017 | RCT | 232 | 28.00 | 16 week | Shamacupuncture | FSH,LH,T,SAS,SDS,BMI,Ovulation Rate |
| Pastore 2011(9) | RCT | 38 | 28.00 | 8 week | Acupuncture | FSH,LH,LH/FSH |
| Pastore 2011 | RCT | 43 | 26.50 | 8 week | Shamacupuncture | FSH,LH,LH/FSH |
| He Xiaoxia 2019(10) | RCT | 40 | 28.00 | 3 menstrual cycle | Letrozole | FSH,LH,LH/FSH,T,BMI,Ovulation Rate |
| He Xiaoxia 2019 | RCT | 40 | 32.00 | 3 menstrual cycle | Chinese Medicine and Letrozole | FSH,LH,LH/FSH,T,BMI,Ovulation Rate |
| He Xiaoxia 2019 | RCT | 40 | 29.00 | 3 menstrual cycle | Electroacupuncture and Chinese Medicine and Letrozole | FSH,LH,LH/FSH,T,BMI,Ovulation Rate |
| Liu Min 2024(11) | RCT | 40 | 32.20 | 3 menstrual cycle | Acupuncture and Chinese Medicine | LH,T,Ovulation Rate |
| Liu Min 2024 | RCT | 40 | 32.50 | 3 menstrual cycle | Clomiphene | LH,T,Ovulation Rate |
| Chen Lifang 2020(12) | RCT | 43 | 29.62 | 12 week | Acupuncture and Diane-35 | Ovulation Rate |
| Chen Lifang 2020 | RCT | 43 | 29.65 | 12 week | Diane-35 | Ovulation Rate |
| Chen Jiao 2024(13) | RCT | 56 | 26.90 | 3 menstrual cycle | Electroacupuncture and Clomiphene | FSH,LH,LH/FSH,T,Ovulation Rate |
| Chen Jiao 2024 | RCT | 54 | 26.80 | 3 menstrual cycle | Clomiphene | FSH,LH,LH/FSH,T,Ovulation Rate |
| Li Ning 2017(14) | RCT | 53 | 26.61 | 3 menstrual cycle | Electroacupuncture | FSH,LH,T,Ovulation Rate |
| Li Ning 2017 | RCT | 53 | 27.53 | 3 menstrual cycle | Acupuncture | FSH,LH,T,Ovulation Rate |
| Li Ning 2017 | RCT | 53 | 27.61 | 3 menstrual cycle | Clomiphene | FSH,LH,T,Ovulation Rate |
| Li Xiaojing 2021(15) | RCT | 33 | 29.63 | 3 menstrual cycle | Acupuncture and Chinese Medicine | FSH,LH,T,Ovulation Rate |
| Li Xiaojing 2021 | RCT | 33 | 29.53 | 3 menstrual cycle | Clomiphene | FSH,LH,T,Ovulation Rate |
| Chen Pengdian 2017(16) | RCT | 30 | 28.33 | 3 menstrual cycle | Acupuncture and Clomiphene | Ovulation Rate |
| Chen Pengdian 2017 | RCT | 30 | 28.33 | 3 menstrual cycle | Clomiphene | Ovulation Rate |
| Luo Jianlin 2020(17) | RCT | 34 | 32.54 | 3 month | Acupuncture and Chinese Medicine | Ovulation Rate |
| Luo Jianlin 2020 | RCT | 34 | 32.16 | 3 month | Clomiphene | Ovulation Rate |
| Xiao Zhaohua 2022(18) | RCT | 40 | 23.30 | 3 menstrual cycle | Acupuncture and Chinese Medicine | Ovulation Rate |
| Xiao Zhaohua 2022 | RCT | 40 | 23.80 | 3 menstrual cycle | Chinese Medicine | Ovulation Rate |
| Zhao Meilan 2014(19) | RCT | 24 | 26.00 | 4 menstryal cycle | Electroacupuncture | Ovulation Rate |
| Zhao Meilan 2014 | RCT | 24 | 26.00 | 4 menstryal cycle | Shamacupuncture | Ovulation Rate |
| Yu Liqing 2018(20) | RCT | 38 | 30.00 | 3 menstrual cycle | Acupuncture and Clomiphene | Ovulation Rate |
| Yu Liqing 2018 | RCT | 37 | 29.00 | 3 menstrual cycle | Clomiphene | Ovulation Rate |
| Yang Hongwei 2022(21) | RCT | 30 | 30.20 | 6 menstrual cycle | Acupuncture and Chinese Medicine | FSH,LH,T,Ovulation Rate |
| Yang Hongwei 2022 | RCT | 30 | 29.80 | 6 menstrual cycle | Chinese Medicine | FSH,LH,T,Ovulation Rate |
| Yang Hongwei 2022 | RCT | 30 | 29.90 | 6 menstrual cycle | Acupuncture | FSH,LH,T,Ovulation Rate |
| Ren Qiuwei 2024(22) | RCT | 40 | 31.70 | 3 menstrual cycle | Acupuncture and Chinese Medicine | FSH,LH,LH/FSH,HOMA-IR,Ovulation Rate |
| Ren Qiuwei 2024 | RCT | 40 | 31.43 | 3 menstrual cycle | Diane-35 and Metformin | FSH,LH,LH/FSH,HOMA-IR,Ovulation Rate |
| Yao Fang 2024(23) | RCT | 38 | 33.56 | 3 month | Acupuncture and Chinese Medicine | FSH,LH,T,Ovulation Rate |
| Yao Fang 2024 | RCT | 37 | 33.59 | 3 month | Diane-35 and Metformin | FSH,LH,T,Ovulation Rate |
| Wang Chenye 2016(24) | RCT | 49 | 29.43 | 3 month | Acupuncture and Chinese Medicine | HOMA-IR,BMI,Ovulation Rate |
| Wang Chenye 2016 | RCT | 40 | 29.43 | 3 month | Chinese Medicine | HOMA-IR,BMI,Ovulation Rate |
| Wang Chenye 2016 | RCT | 35 | 29.43 | 3 month | Metformin | HOMA-IR,BMI,Ovulation Rate |
| Peng Yanli 2018(25) | RCT | 60 | 26.41 | 3 menstrual cycle | Electroacupuncture and Chinese Medicine | FSH,LH,T,Ovulation Rate |
| Peng Yanli 2018 | RCT | 60 | 25.14 | 3 menstrual cycle | Clomiphene | FSH,LH,T,Ovulation Rate |
| Lin Wanshan 2018(26) | RCT | 30 | 28.70 | 3 menstrual cycle | Acupuncture | LH,LH/FSH,T,Ovulation Rate |
| Lin Wanshan 2018 | RCT | 30 | 28.20 | 3 menstrual cycle | Clomiphene | LH,LH/FSH,T,Ovulation Rate |
| Zhou Hao 2021(27) | RCT | 40 | 28.64 | 3 month | Acupuncture and Clomiphene and Chinese Medicine | FSH,LH,Ovulation Rate |
| Zhou Hao 2021 | RCT | 40 | 28.51 | 3 month | Chinese Medicine and Clomiphene | FSH,LH,Ovulation Rate |
| Zhou Hao 2021 | RCT | 40 | 28.90 | 3 month | Clomiphene | FSH,LH,Ovulation Rate |
| Li Yonghong 2022(28) | RCT | 62 | 28.05 | 3 menstrual cycle | Acupuncture and Letrozole | FSH,LH,LH/FSH,T,Ovulation Rate |
| Li Yonghong 2022 | RCT | 62 | 27.64 | 3 menstrual cycle | Letrozole | FSH,LH,LH/FSH,T,Ovulation Rate |
| Huang Aiqin 2021(29) | RCT | 30 | 30.54 | 3 menstrual cycle | Acupuncture and Diane-35 | FSH,LH,T,Ovulation Rate |
| Huang Aiqin 2021 | RCT | 30 | 30.89 | 3 menstrual cycle | Diane-35 | FSH,LH,T,Ovulation Rate |
| Chen Yuting 2017(30) | RCT | 30 | 29.00 | 3 menstrual cycle | Acupuncture | FSH,LH,LH/FSH,T,Ovulation Rate |
| Chen Yuting 2017 | RCT | 30 | 30.00 | 3 menstrual cycle | Letrozole | FSH,LH,LH/FSH,T,Ovulation Rate |
| Ma Saihua 2020(31) | RCT | 56 | 29.00 | 3 month | Acupuncture | Ovulation Rate |
| Ma Saihua 2020 | RCT | 54 | 28.00 | 3 month | Letrozole | Ovulation Rate |
| Ma Saihua 2020 | RCT | 57 | 28.00 | 3 month | Acupuncture and Letrozole | Ovulation Rate |
| Liu Lingzhi 2021(32) | RCT | 35 | 27.12 | 3 month | Acupuncture and Metformin | FSH,LH,T,Ovulation Rate |
| Liu Lingzhi 2021 | RCT | 35 | 26.95 | 3 month | Metformin | FSH,LH,T,Ovulation Rate |
| Yang Yuehong 2005(33) | RCT | 66 | 26.00 | 3 menstrual cycle | Acupuncture | Ovulation Rate |
| Yang Yuehong 2005 | RCT | 60 | 25.00 | 3 menstrual cycle | Clomiphene | Ovulation Rate |
| Wu Dan 2023(34) | RCT | 30 | 21.89 | 3 month | Acupuncture and Letrozole | Ovulation Rate,SAS,SDS |
| Wu Dan 2023 | RCT | 30 | 22.33 | 3 month | Letrozole | Ovulation Rate,SAS,SDS |
| Ying Cong 2019(35) | RCT | 45 | 28.89 | 2 month | Acupuncture and Chinese Medicine | LH,T,Ovulation Rate |
| Ying Cong 2019 | RCT | 45 | 28.54 | 2 month | Clomiphene | LH,T,Ovulation Rate |
| Liang Zhuo 2015(36) | RCT | 30 | 28.64 | 16 week | Acupuncture and Clomiphene | FSH,LH,LH/FSH,HOMA-IR,BMI,Ovulation Rate |
| Liang Zhuo 2015 | RCT | 30 | 27.53 | 16 week | Clomiphene | FSH,LH,LH/FSH,HOMA-IR,BMI,Ovulation Rate |
| Yan Caiping 2019(37) | RCT | 30 | 26.50 | 3 menstrual cycle | Acupuncture and Clomiphene | Ovulation Rate |
| Yan Caiping 2019 | RCT | 30 | 26.10 | 3 menstrual cycle | Clomiphene | Ovulation Rate |
| Zhang Moli 2020(38) | RCT | 32 | 26.00 | 2 month | Acupuncture and Diane-35 | Ovulation Rate |
| Zhang Moli 2020 | RCT | 32 | 26.00 | 2 month | Diane-35 | Ovulation Rate |
| Wang Chongyang 2015(39) | RCT | 40 | 28.10 | 4 month | Acupuncture and Clomiphene | FSH,LH,T,Ovulation Rate |
| Wang Chongyang 2015 | RCT | 40 | 28.90 | 4 month | Clomiphene | FSH,LH,T,Ovulation Rate |
| Yuan Shufen 2019(40) | RCT | 30 | 26.00 | 1 menstrual cycle | Electroacupuncture and Chinese Medicine | LH,LH/FSH,T,Ovulation Rate |
| Yuan Shufen 2019 | RCT | 30 | 23.00 | 1 menstrual cycle | Chinese Medicine | LH,LH/FSH,T,Ovulation Rate |
| Yuan Shufen 2019 | RCT | 30 | 24.00 | 1 menstrual cycle | Clomiphene | LH,LH/FSH,T,Ovulation Rate |
| Ren Fenglan 2022(41) | RCT | 79 | 28.00 | 3 month | Acupuncture and Chinese Medicine | LH,T,Ovulation Rate |
| Ren Fenglan 2022 | RCT | 78 | 29.00 | 3 month | Acupuncture | LH,T,Ovulation Rate |
| Ren Fenglan 2022 | RCT | 75 | 27.00 | 3 month | Chinese Medicine | LH,T,Ovulation Rate |
| Yang Bingwei 2022(42) | RCT | 46 | 29.67 | 3 menstrual cycle | Acupuncture and Chinese Medicine | FSH,LH,T,Ovulation Rate |
| Yang Bingwei 2022 | RCT | 46 | 29.65 | 3 menstrual cycle | Shamacupuncture and Chinese Medicine | FSH,LH,T,Ovulation Rate |
| Wen Lina 2023(43) | RCT | 42 | 22.18 | 6 menstrual cycle | Acupuncture and Letrozole | FSH,LH,T,Ovulation Rate |
| Wen Lina 2023 | RCT | 42 | 21.75 | 6 menstrual cycle | Letrozole | FSH,LH,T,Ovulation Rate |

References:

1. LI Saisai. Metformin and Auxiliary Acupuncture in the treatment of Obese Women Infertility with Polycystic Ovary Syndrome for 75 Cases. Chinese Medicine Modern Distance Education of China 2015;13(6):78–9.

2. MA Hong, QUAN Xiaohong, CHEN Xiuhua, DONG Ying. Flyingneedlingtherapycombined withclomipheneforovulationfailureinpolycysticovarysyndrome:arandomizedcontrolledtrial. Chinese Acupuncture & Moxibustion 2016;36(11):1161–5.

3. XU Juan, ZUO Yu. Efficacy of acupuncture as adjunctive treatment on infertility patients with polycystic ovary syndrome. Chinese Acupuncture& Moxibustion 2018;38(4):358–61, 363.

4. YIN Yan, ZHANG Yingchun, ZHANG Hua, JIANG Duosheng, GUO Guirong. Clinical therapeutic effects of acupuncture combined with Chinese herbal medicine on infertility of polycystic ovary syndrome in the patients with ovulation induction with letrozole. Chinese Acupuncture& Moxibustion 2018;38(1):27–32.

5. ZHUO Yuanyuan, WU Jiaman, LIN Wanshan, PI Min, CHEN Pengdian, YANG Zhuoxin. The “regulatingconception-governorvessel”acupuncturemethodforinfertilityofpolycysticovarian syndrome. Chinese Acupuncture& Moxibustion 2016;36(12):1237–41.

6. CHEN Ming, ZHANG Hua, ZHANG Ying-chun. Clinical Observation of Acupuncture plus Medication for Obesity-type Polycystic Ovary Syndrome. Shanghai Journal of Acupuncture and Moxibustion 2015;34(4):310–3.

7. Pan W, Li F, Wang Q, Huang Z, Yan Y, Zhao L, et al. A randomized sham-controlled trial of manual acupuncture for infertile women with polycystic ovary syndrome. Integr Med Res 2022;11(2):100830.

8. Wu X-K, Stener-Victorin E, Kuang H-Y, Ma H-L, Gao J-S, Xie L-Z, et al. Effect of acupuncture and clomiphene in Chinese women with polycystic ovary syndrome. JAMA 2017;317(24):2502–14.

9. Pastore LM, Williams CD, Jenkins J, Patrie JT. True and Sham Acupuncture Produced Similar Frequency of Ovulation and Improved LH to FSH Ratios in Women with Polycystic Ovary Syndrome. J Clin Endocrinol Metab 2011;96(10):3143–50.

10. HE Xiaoxia, FANG You, LIU Li. Randomized controlled study on the effect of different treatment regimens on endometrial receptivity in infertile patients with polycystic ovary syndrome. Journal of Li-shizhen Traditional Chinese Medicine 2019;30(10):2424–6.

11. LIU Min. Observation on the curative effect of traditional Chinese medicine cycle adjustment method combined with acupuncture in the treatment of polycystic ovary syndrome with ovulation disorder infertility. Journal of Practical Traditional Chinese Medicine 2024;40(3):420–2.

12. CHEN Lifang. Efficacy of oral ethinyl estradiol cyproterone tablets combined with acupuncture in the treatment of polycystic ovary syndrome complicated with infertility. Contemporary Medical Symposium 2020;18(10):93–4.

13. CHEN Jiao, FAN Huaying, ZENG Jiuzhi, LIU Yu, HU Jinqun, ZHOU Minqing, et al. Clinical efficacy observation of electroacupuncture cycle therapy combined with clomiphene citrate treatment based on the theory of ‘Ren zhu Baotai’ on polycystic ovary syndrome. China Journal of Traditional Chinese Medicine and Pharmacy 2024;39(1):496–9.

14. LI Ning, ZHANG Ying. Observation on the curative effect of Ziwuliuzhu acupoint opening method in the treatment of ovulation disorder of polycystic ovary syndrome. Beijing Journal of Traditional Chinese Medicine 2017;36(1):74–6.

15. LI Xiao-Jing, ZHANG Hao, ZHANG Zhi-Ling, GUO Bei-Ning. Clinical Observation of Zuogui Drink and HuanglianEjiao Decoction Combined with Acupuncture in Treatment of Infertility Associated with Polycystic Ovary Syndrome. Journal of Guangzhou University of Traditional Chinese Medicine 2021;38(10):2145–51.

16. CHEN Pengdian, YANG Zhuoxin, LIU Fang, YAN Bin, QIU Tingting. Clinical observation of Clomiphene Citrate Tablets plus conception and governor vessels acupuncture in the treatment for ovulation of polycystic ovarian syndrome. China Medical Herald 2017;14(7):91–4.

17. LUO Jianlin. Clinical study on traditional Chinese medicine combined with acupuncture in the treatment of infertility with polycystic ovary syndrome. Friends of Health 2020;(18):46.

18. XIAO Zhao-hua, ZHONG Hai-ying, ZHENG Dong-ying. Clinical study of Ziyin Bushen Decoction combined with acupuncture therapy on patients with polycystic ovary syndrome of kidney yin deficiency type. Guangming Journal of Chinese Medicine 2022;37(23):4284–7.

19. ZHAO Meilan, LIANG Ruining. Randomized single-blind clinical controlled study of electroacupuncture for ovulation induction in polycystic ovary syndrome. Practical Clinical Journal of Integrated Traditional Chinese and Western Medicine 2014;14(8):66–8.

20. YU Liqing, CAO Lianying, XIE Jing, SHI Yin. Therapeutic effects on ovulation and reproduction promotion with acupuncture and clomiphene in polycystic ovary syndrome. Chinese Acupuncture& Moxibustion 2018;38(3):263–8.

21. YANG Hongwei, ZHAO Xuejuan, WEI Meixia, LIU Xiuming. Clinical Study on Yishen Huatan Prescription Combined with Acupuncture for Infertility Due to Polycystic Ovary Syndrome of Kidney Deficiency with Phlegm-Dampness Type. New Chinese Medicine 2022;54(17):153–7.

22. REN Qiuwei, SUN Jie, CUI Shaobin, CHEN Daomang. Effects of Wuzi Xiezhuo decoction combined with acupuncture on glucose and lipid metabolism and ovulation rate in obese polycystic ovary syndrome. Chinese Archives of Traditional Chinese Medicine 2024;1–7.

23. YAO Fang. Clinical Observation of Cangfu Daotan Pill Combined with Acupuncture in the Treatment of Polycystic Ovary Syndrome with Spleen Deficiency and Phlegm Dampness. Journal of Practical Traditional Chinese Medicine 2024;40(4):623–6.

24. WANG Chenye, SUN Xin, DING Caifei, SHEN Yinghong. Effect of Cangfu Daotan Decoction Combined with Acupuncture on Glucose and Lipid Metabolism and Ovulation Rate in Patients with Obese Polycystic Ovary Syndrome. Modern Journal of Integrated Traditional Chinese and Western Medicine 2016;25(36):4056–8.

25. PENG Yan-li, SHEN Yan, LUO Lan, HU Yan-bing. Effect of Bushen Huoxue recipe combined with electroacupuncture on endocrine hormone and pregnancy outcome in infertile patients with polycystic ovary syndrome. Guangxi Medical Journal 2018;40(7):795–8.

26. LIN Wanshan, PI Min, ZHUO Yuanyuan, YANG Zhuoxin. Observation on the Effect of Tiaoren Tongdu Acupuncture in the Treatment of Polycystic Ovary Syndrome Infertility of Kidney Yang Deficiency Type. Guiding Journal of Traditional Chinese Medicine and Pharmacy 2018;24(4):80–2.

27. ZHOU Hao, SHAO Suxia. Clinical Study on Acupuncture Method of Regulating Conception Vessel and Governor Vessel Combined with Dingkun Pills for Polycystic Ovarian Disease Syndrome with Infertility. New Chinese Medicine 2021;53(2):106–11.

28. LI Yonghong, KE Yan, LIU Yujie, FENG Ting, LI Yuchang, LI Hui, et al. Efficacy of Regulating Ren Meridian and Dredging Du Meridian Acupuncture Combined with Letrozole in the Treatment of Infertility Caused by Polycystic Ovary Syndrome with Kidney-Yang Deficiency Type and Its Effect on Lipid Metabolism and Sex Hormone Levels. Journal of Liaoning University of Traditional Chinese Medicine 2022;24(7):171–5.

29. HUANG Aiqin. Effect of Lai ’s Tongyuan acupuncture combined with conventional western medicine on infertility patients with polycystic ovary syndrome. Medical Journal of Chinese People’s Health 2021;33(15):91–3.

30. CHEN Yu-ting, Li Yue-mei, LUO Jiao-long, CHEN Yu-jia, ZHENG Shu-zhen. Clinical Observation of Tong Yuan Needling Method plus Letrozol and HCG for Refractory Sterility Caused by Polycystic Ovary Syndrome. Shanghai Journal of Acupuncture and Moxibustion 2017;36(6):692–6.

31. MA Sai-hua, DOU Zhen, SONG Jia-yi, LIU Sha-sha, TIAN Xia, FU Yu. Clinical Observation of Mind-refreshing and Orifice-opening Needling Combined with Western Medication for Polycystic Ovary Syndrome of Kidney Deficiency and Liver Depression Syndrome. Shanghai Journal of Acupuncture and Moxibustion 2020;39(9):1128–32.

32. LIU Lingzhi, CHEN Chaoying, HE Shuguang. Effect of acupuncture on ovarian function and sex hormone levels in patients with polycystic ovary syndrome. Laboratory Medicine and Clinic 2021;18(11):1596–8.

33. YANG Yueh-ong, HONG Jian-yun, WEI Da-you, CHEN Xue-mei, LIN Jian. Acupuncture on polycystic ovarian syndrome-induced infertility. Journal of Guangdong Medical College 2005;(4):377–8.

34. WU Dan, LUO Jian, PANG Zhuochao, YOU Zhehui. Clinical effect of acupuncture on infertility of polycystic ovary syndrome with kidney deficiency and liver depression. China Modern Medicine 2023;30(20):126–30.

35. YING Cong. Clinical Observation on Acupuncture Combined with Shugan Qutan Sanyu Decoction in the Treatment of Infertility Caused by Polycystic Ovary Syndrome. Journal of Practical Traditional Chinese Medicine 2019;35(8):914–5.

36. LIANG Zhuo. Effect of acupuncture combined with promoting ovulation treatment on pregnancy and ovulation rate of infecundity women with polycystic ovary syndrome of phlegm wet type. Modern Journal of Integrated Traditional Chinese and Western Medicine 2015;24(14):1498–500, 1509.

37. YAN Caiping, FAN Chen, YAN Hong, ZHANG Lixin, ZHONG Yanfen, ZHOU Xiaoxia. Clinical Study of Acupuncture Combined with Clomiphene to Induce Ovulation on Follicular Development and Endometrium of Patients with Polycystic Ovary Syndrome. New Chinese Medicine 2019;51(2):204–6.

38. ZHANG-Moli, WANG Xiao-guang, SHAN Zheng-peng, DONG Bao-qiang. Effects of Acupuncture Combined with Western Medication on Endometrial Thickness, Ovulation Rate and Clinical Pregnancy Rate in Infertile Polycystic Ovary Syndrome Patients. Shanghai Journal of Acupuncture and Moxibustion 2020;39(9):1133–7.

39. WANG Chongyang. Clinical Observation of Acupuncture Combined with Clomiphene in the Treatment of Polycystic Ovary Syndrome. Asia-Pacific Traditional Medicine 2015;11(11):83–5.

40. YUAN Shu-fen, YE Yong-ju, WANG Ting-ting. Effect of Acupuncture plus Medication on Ovary Function in Polycystic Ovary Syndrome Due to Kidney Deficiency and Blood Stagnation. Shanghai Journal of Acupuncture and Moxibustion 2019;38(3):286–9.

41. REN Fenglan, YANG Lianjie, LIU Yuchun. Efficacy Observation of Acupuncture Combined with Medication for Infertility in Polycystic Ovary Syndrome of Kidney Deficiency and Liver Depression Pattern. Shanghai Journal of Acupuncture and Moxibustion 2022;41(1):43–9.

42. YANG Bingyi, SHI Huanli, ZHOU Yichen, LU Qitian, YIN Xiuqi. Clinical observation on efficacy of acupuncture combined with traditional Chinese medicine for treating polycystic ovary syndrome patients with kidney Yang deficiency syndrome. Academic Journal of Shanghai University of Traditional Chinese Medicine 2022;36(S1):88–92.

43. WEN Lina, WANG Miao, ZHOU Yahong. Effect of longsha open and close pivot needle method combined with Letrozole in the treatment of infertility with polycystic ovarian syndrome. Guiding Journal of Traditional Chinese Medicine and Pharmacy 2023;20(30):117–20.

1. **Supplemental file 5.** **Risk of bias of included studies:**


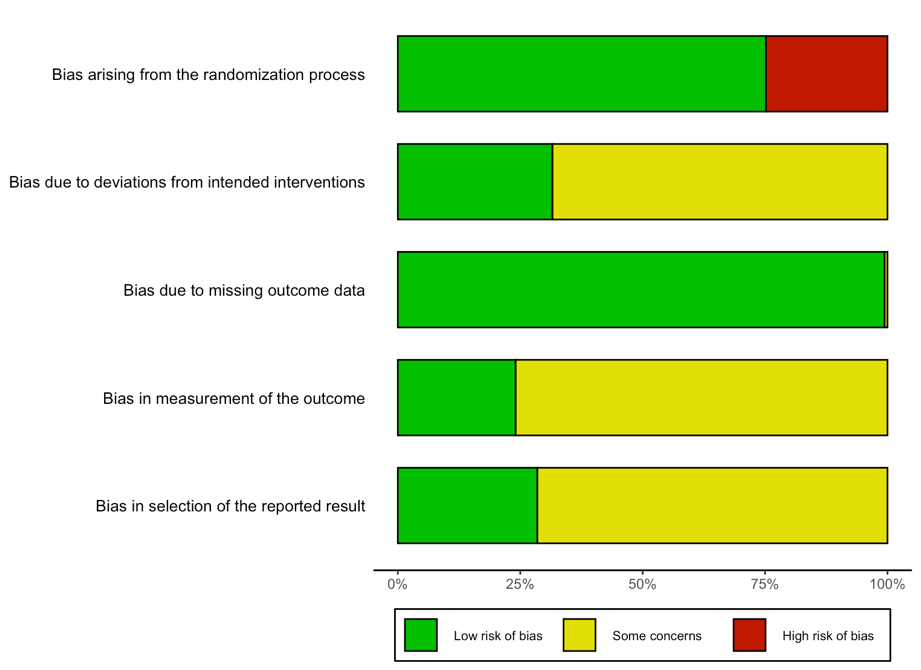

1. **Supplemental file 6. Forest plot of Pairwise Meta-Analysis:**

**FIGURE 6. 1. Mean difference (95% CI) for BMI in acupuncture versus blank groups at the end of treatment. Results are based on a common-effects model.**


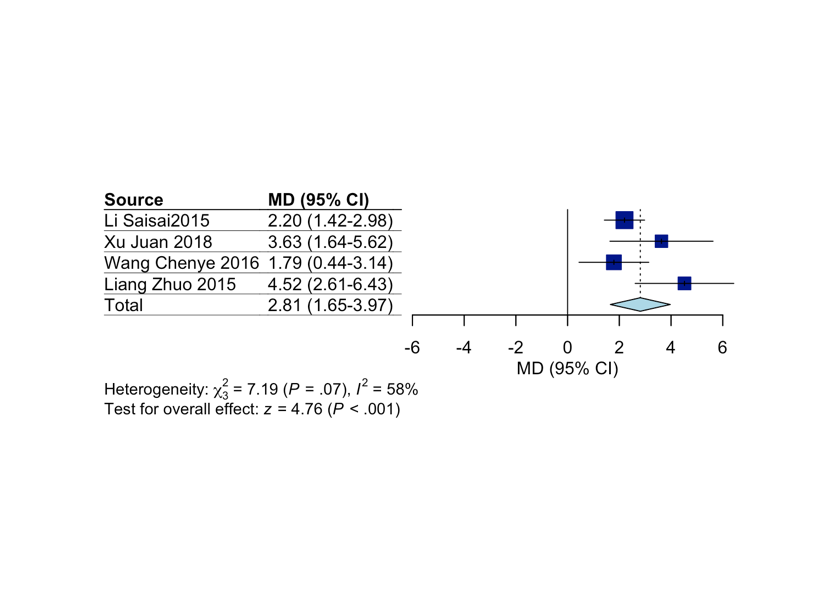


**FIGURE 6. 2. Standardized mean difference (95% CI) for FSH in acupuncture and medicine versus western medicine at the end of treatment. Results are based on a common-effects model.**


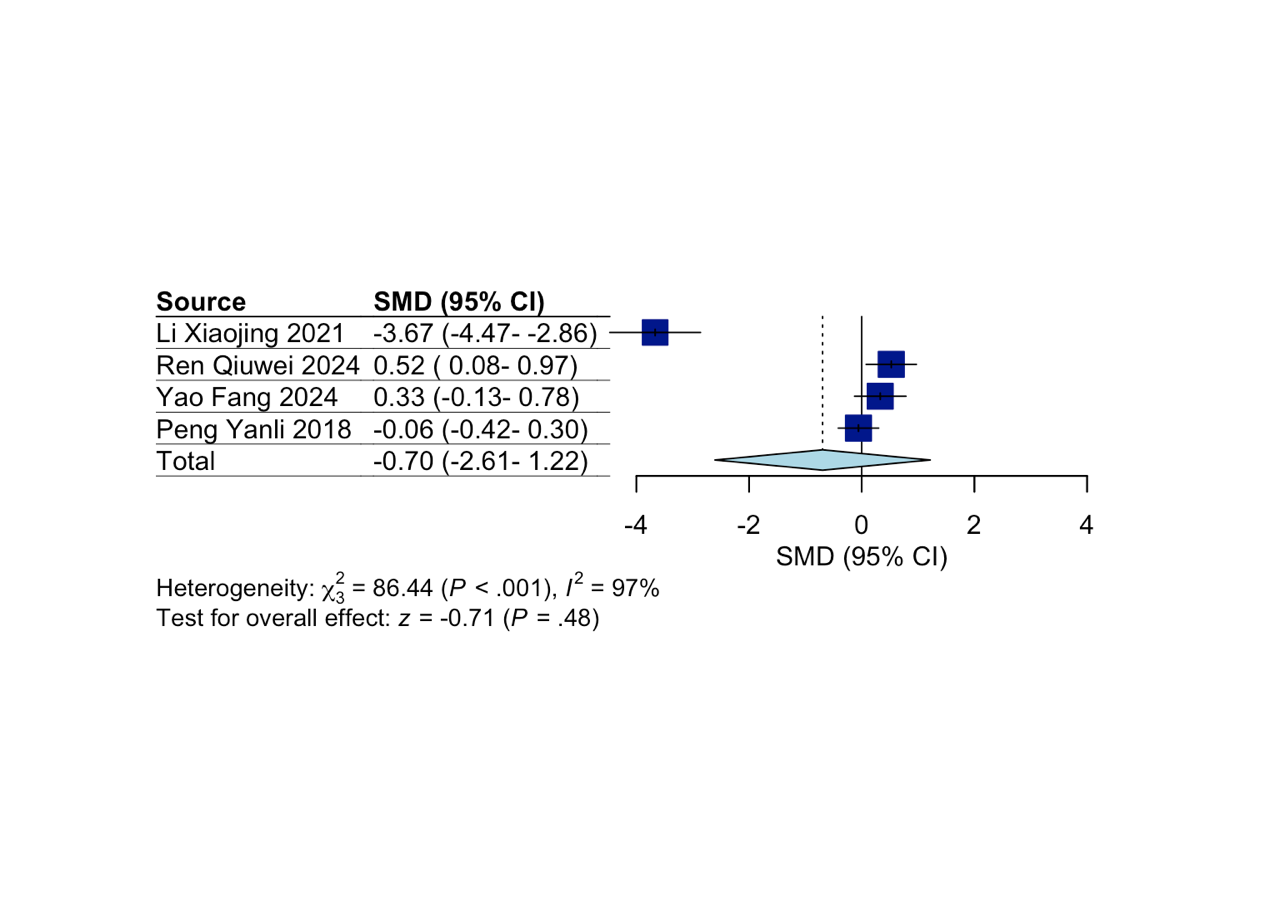


**FIGURE 6. 3.** **Standardized mean difference (95% CI) for FSH in acupuncture versus medicine at the end of treatment. Results are based on a common-effects model.**


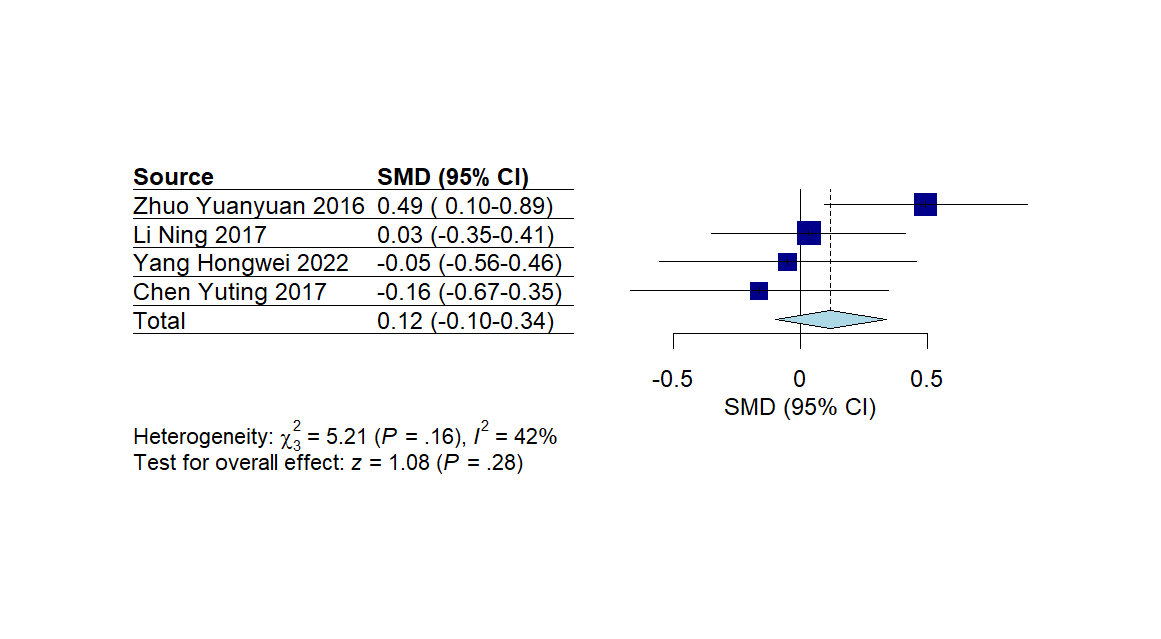


**FIGURE 6. 4. Standardized mean difference (95% CI) for FSH in acupuncture versus sham acupuncture at the end of treatment. Results are based on a common-effects model.**


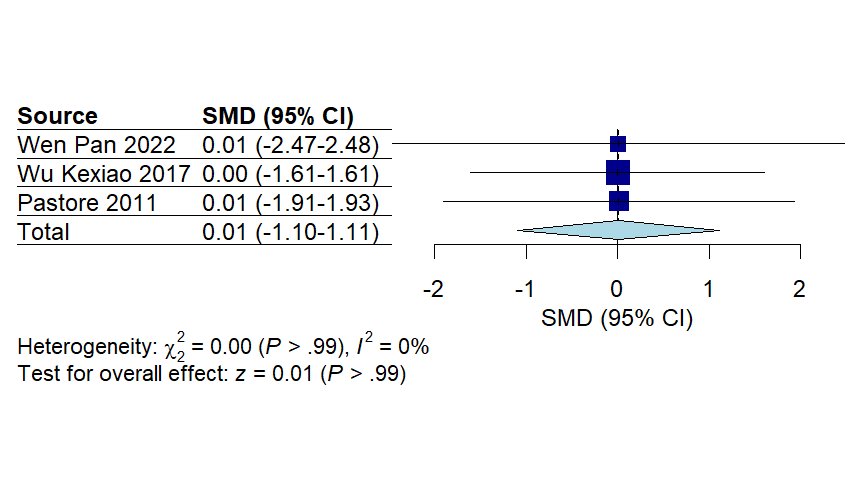


**FIGURE 6. 5 Standardized mean difference (95% CI) for FSH in acupuncture versus blank at the end of treatment. Results are based on a random-effects model.**


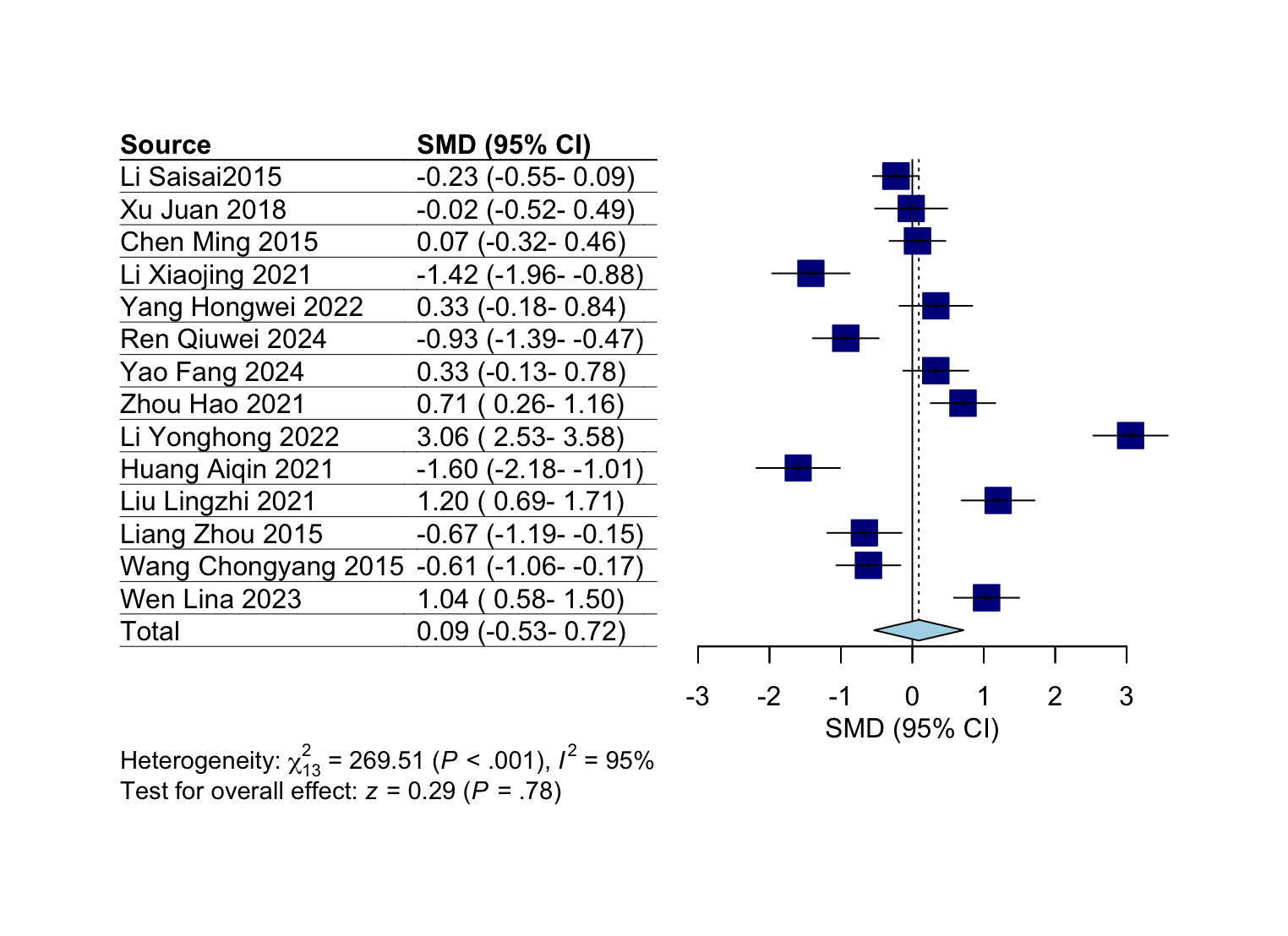


**FIGURE 6. 6. Standardized mean difference (95% CI) for LH in acupuncture versus medicine at the end of treatment. Results are based on a random-effects model.**


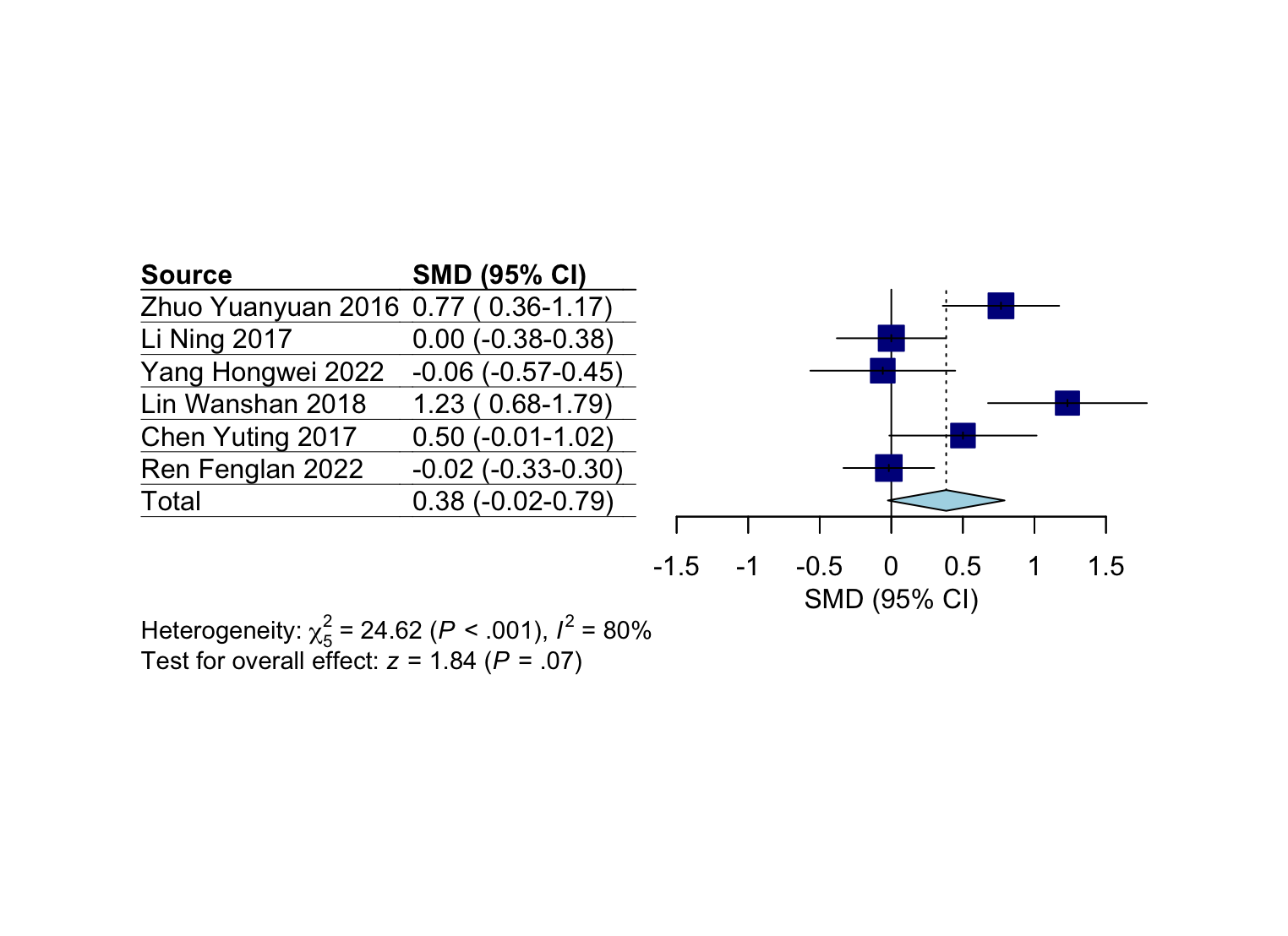


**FIGURE 6. 7. Standardized mean difference (95% CI) for LH in acupuncture and Chinese medicine versus western medicine at the end of treatment. Results are based on a random-effects model.**


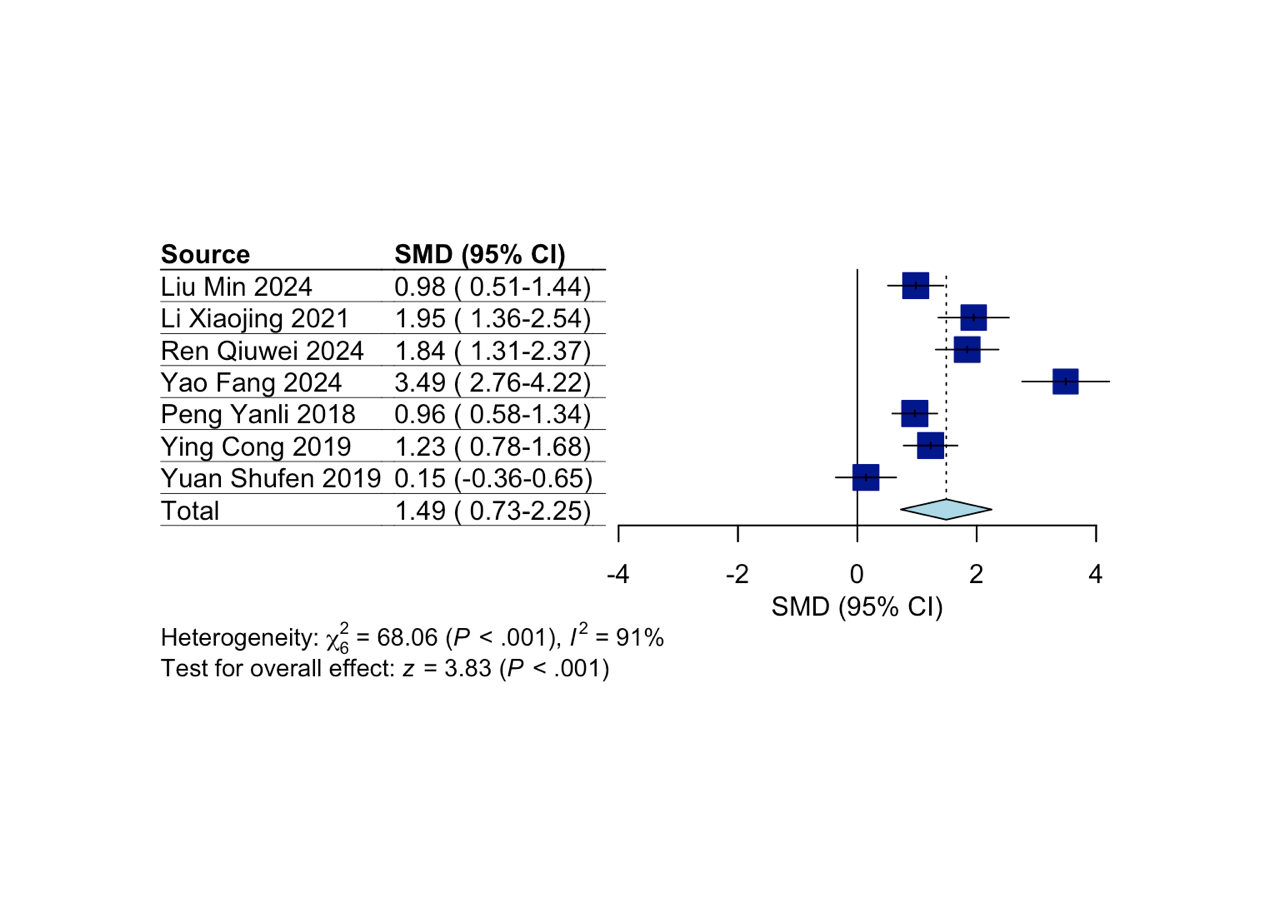


**FIGURE 6. 8. Standardized mean difference (95% CI) for FSH in acupuncture versus sham acupuncture at the end of treatment. Results are based on a random-effects model.**


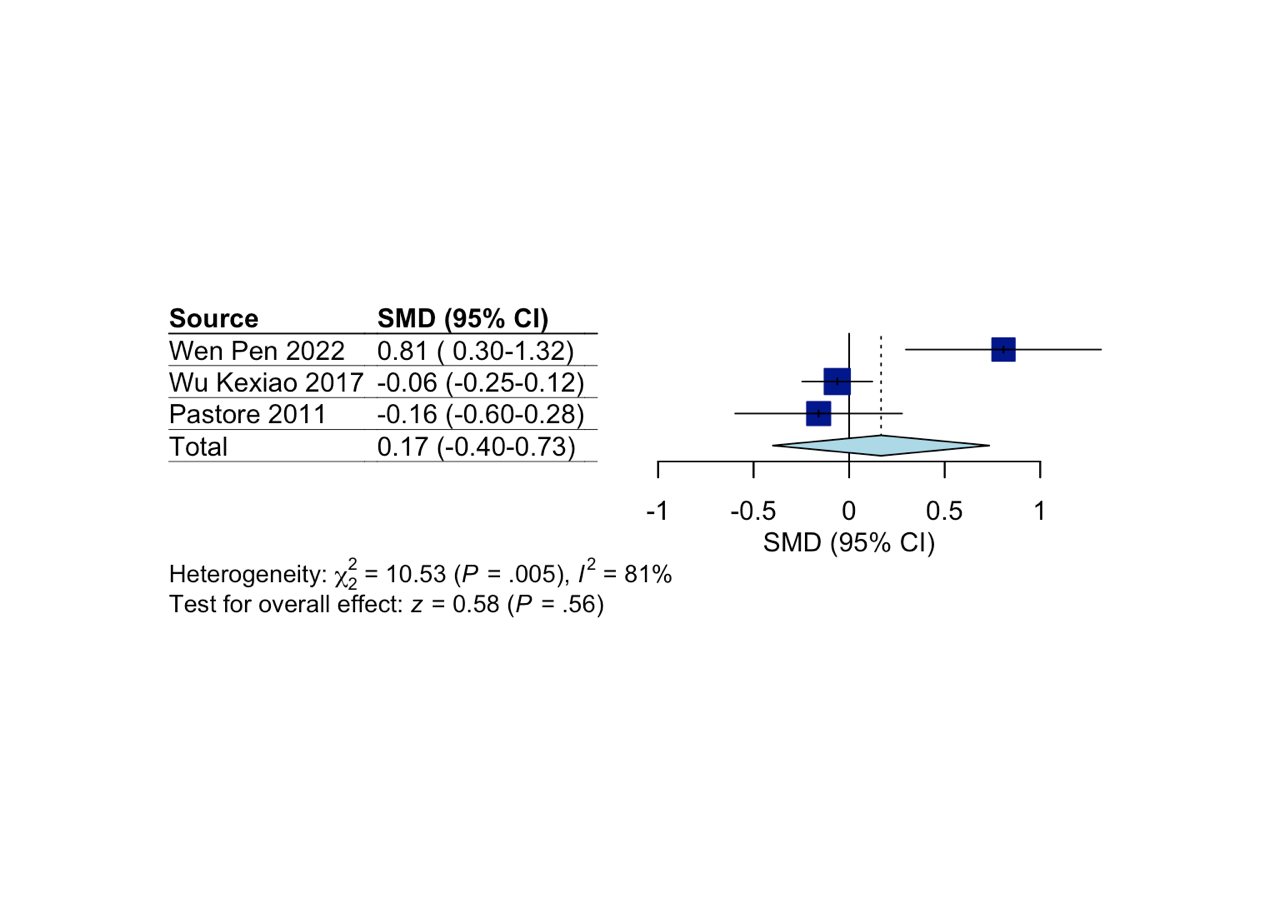


**FIGURE 6. 9. Standardized mean difference (95% CI) for LH in acupuncture versus blank groups at the end of treatment. Results are based on a random-effects model.**


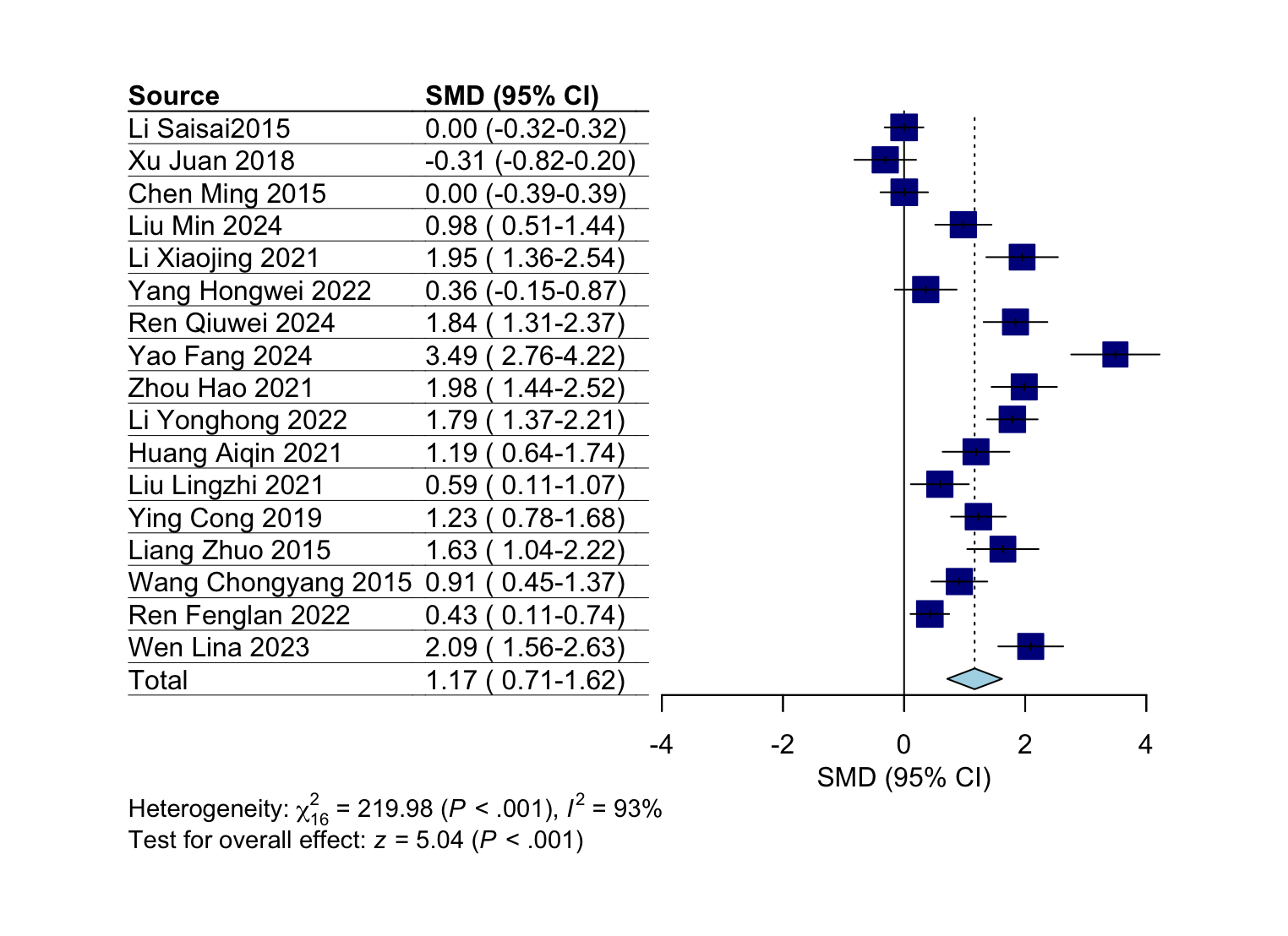


**FIGURE 6. 10. Standardized mean difference (95% CI) for LH/FSH ratio in acupuncture versus blank at the end of treatment. Results are based on a random-effects model.**


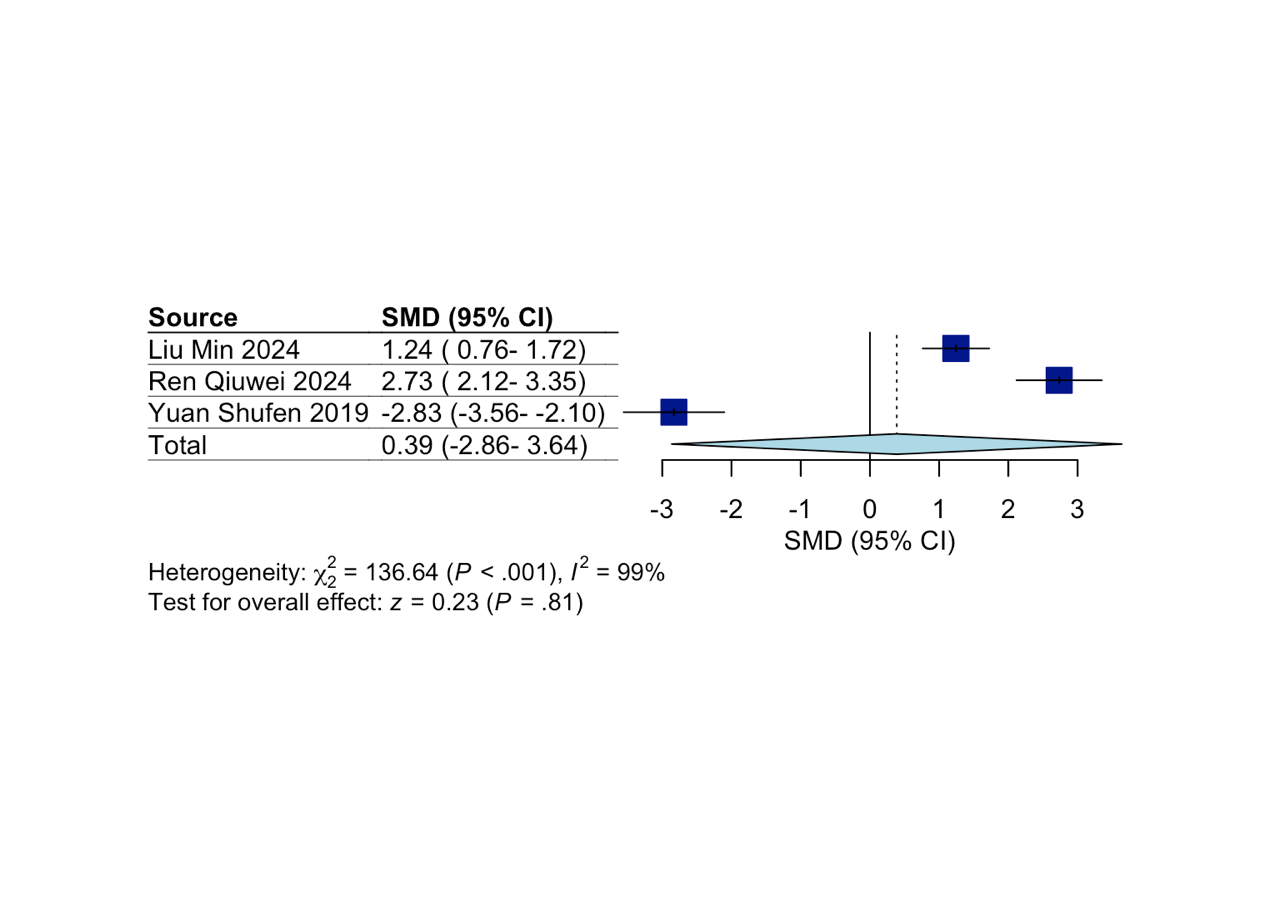


**FIGURE 6. 11 Standardized mean difference (95% CI) for T in acupuncture versus medicine at the end of treatment. Results are based on a random-effects model.**


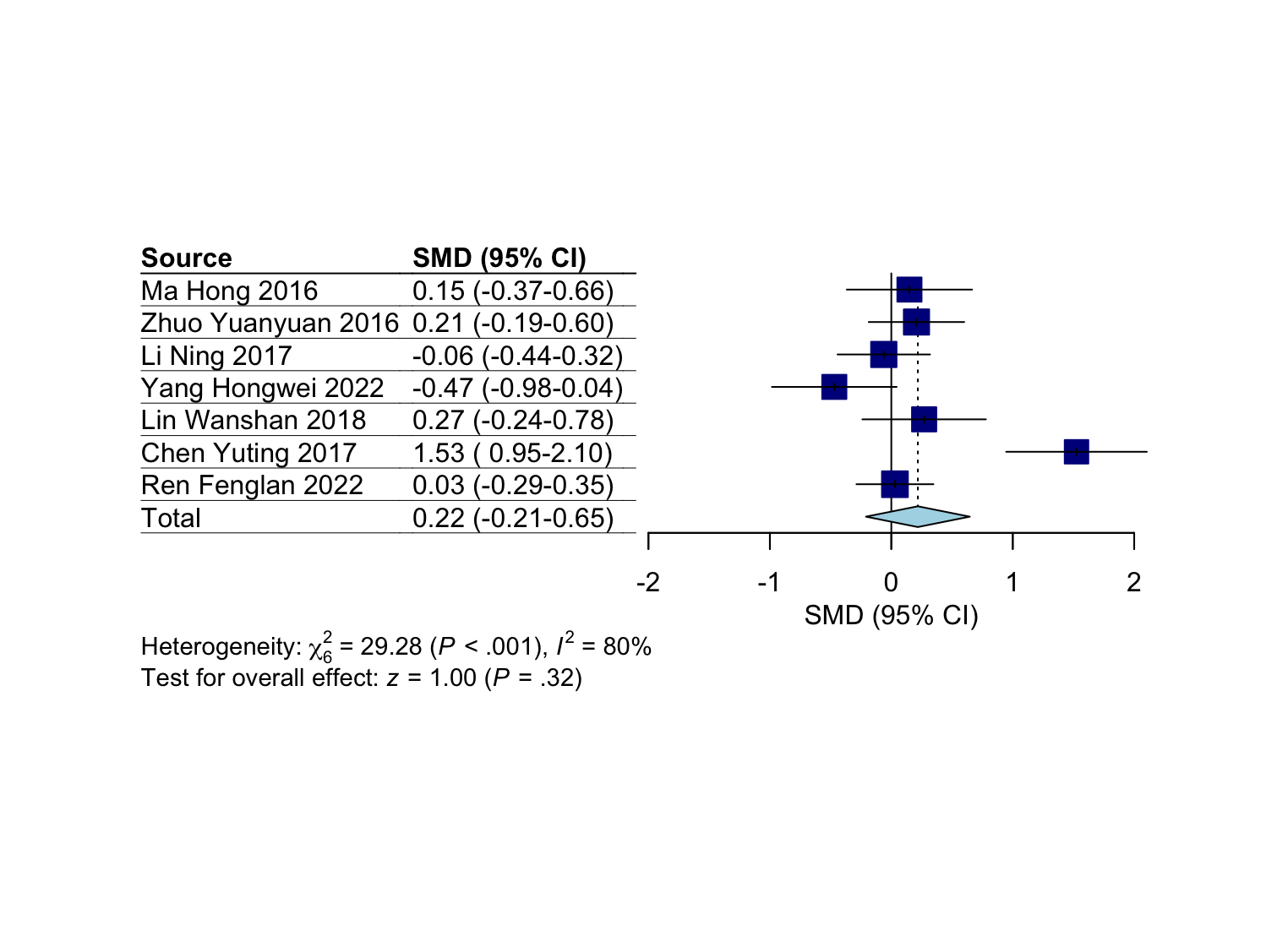


**FIGURE 6. 12 Standardized mean difference (95% CI) for T in acupuncture and Chinese medicine versus Western medicine at the end of treatment. Results are based on a random-effects model.**


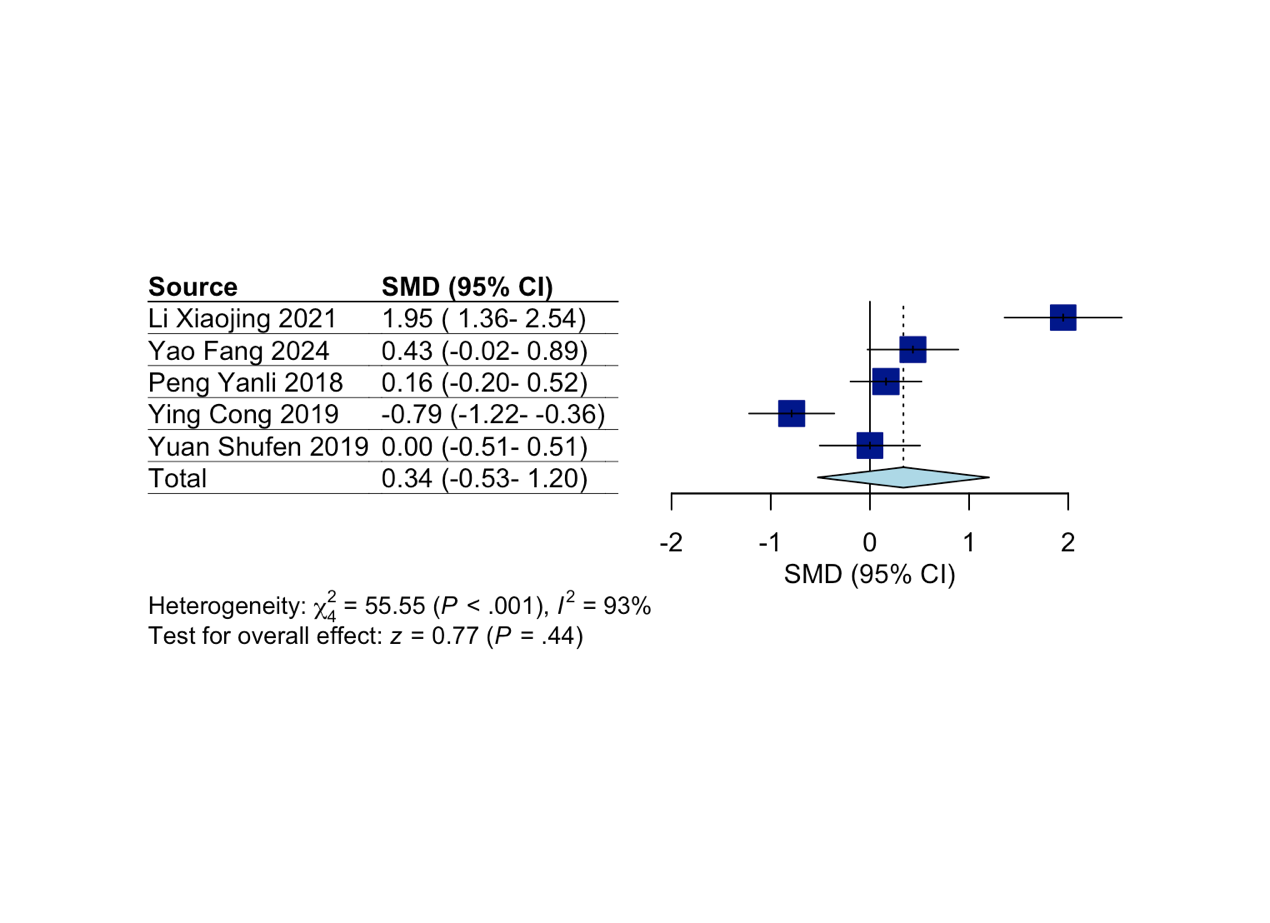


**FIGURE 6. 13 Standardized mean difference (95% CI) for T in acupuncture versus blank at the end of treatment. Results are based on a random-effects model.**


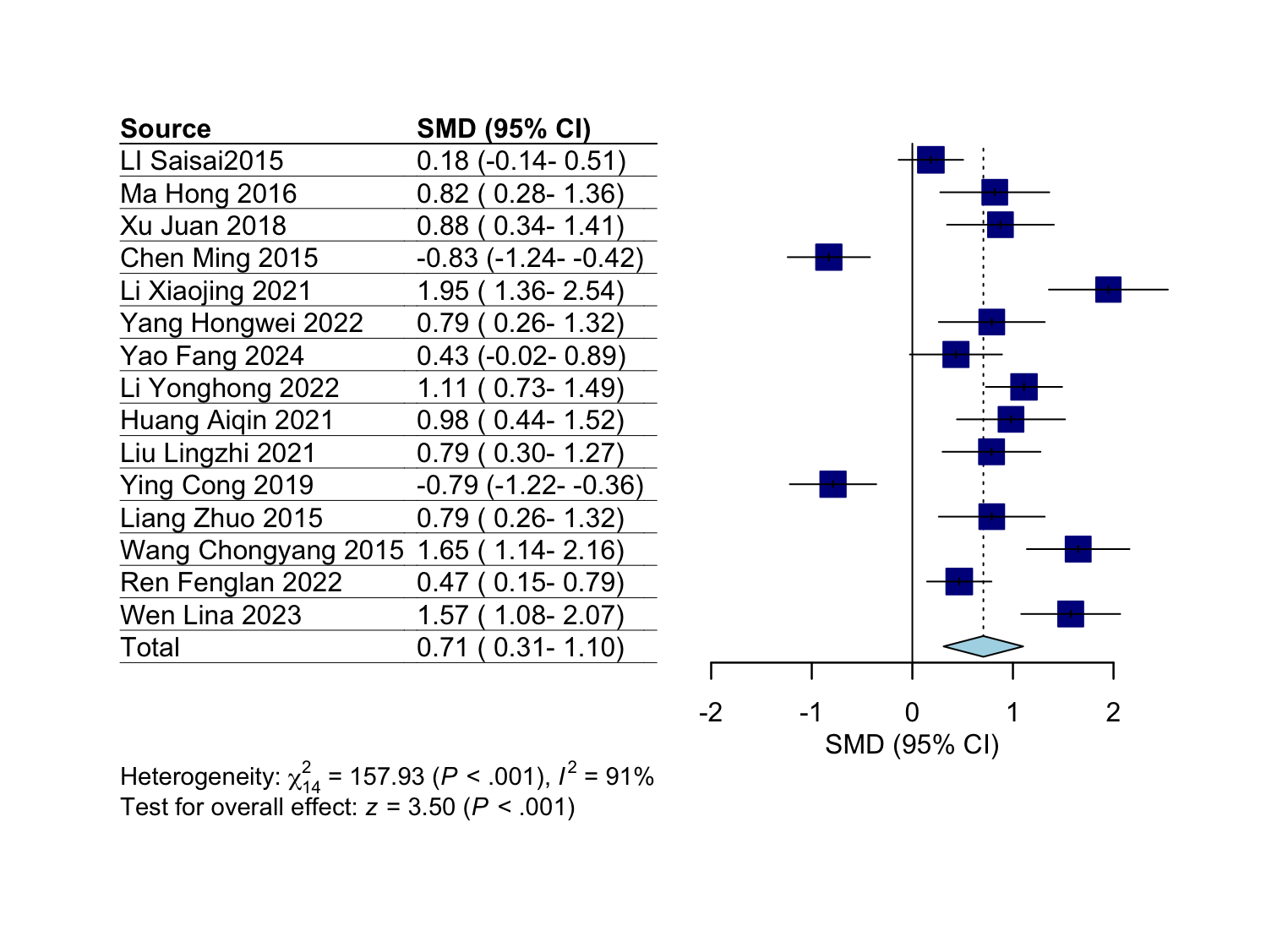


1. **Supplemental file 7. Forest plot of sensitivity analysis：**

**FIGURE7. 1.Sensitivity analysis for ovulation rate in acupuncture versus sham acupuncture**

**
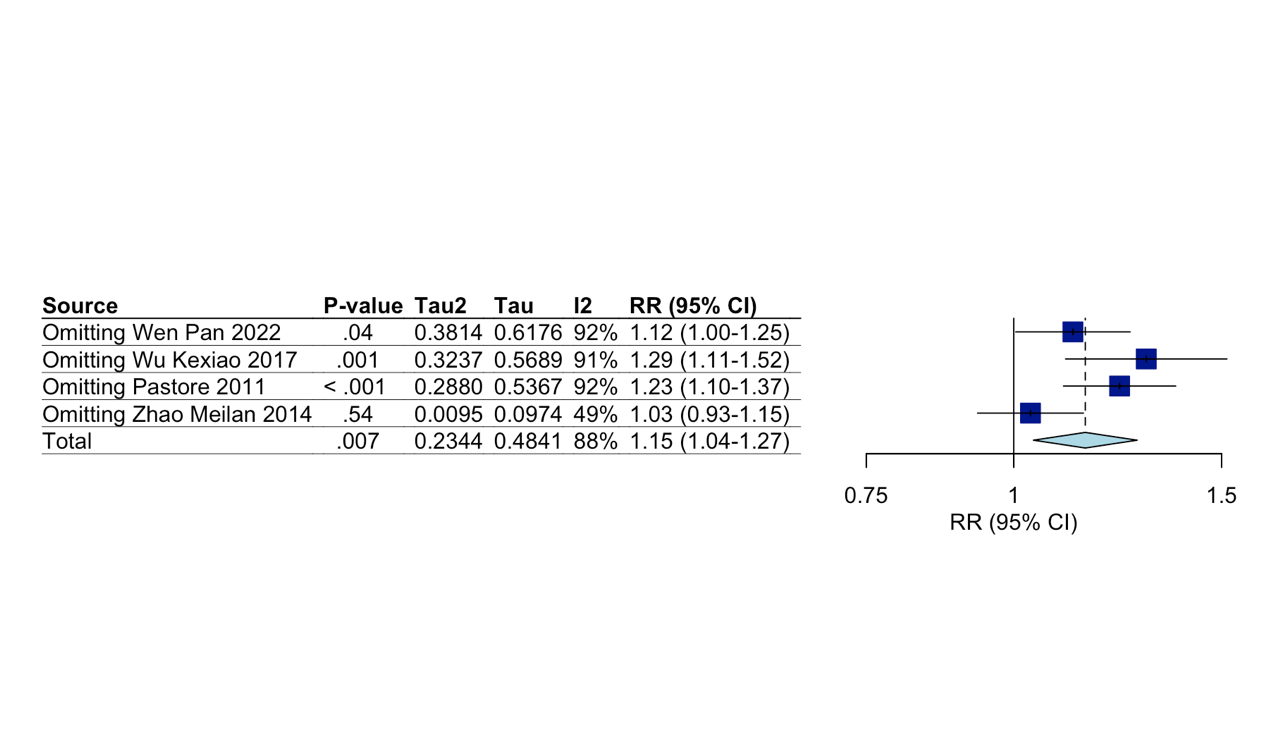
**

**FIGURE7. 2 Sensitivity analysis for ovulation rate in acupuncture versus medicine**

**
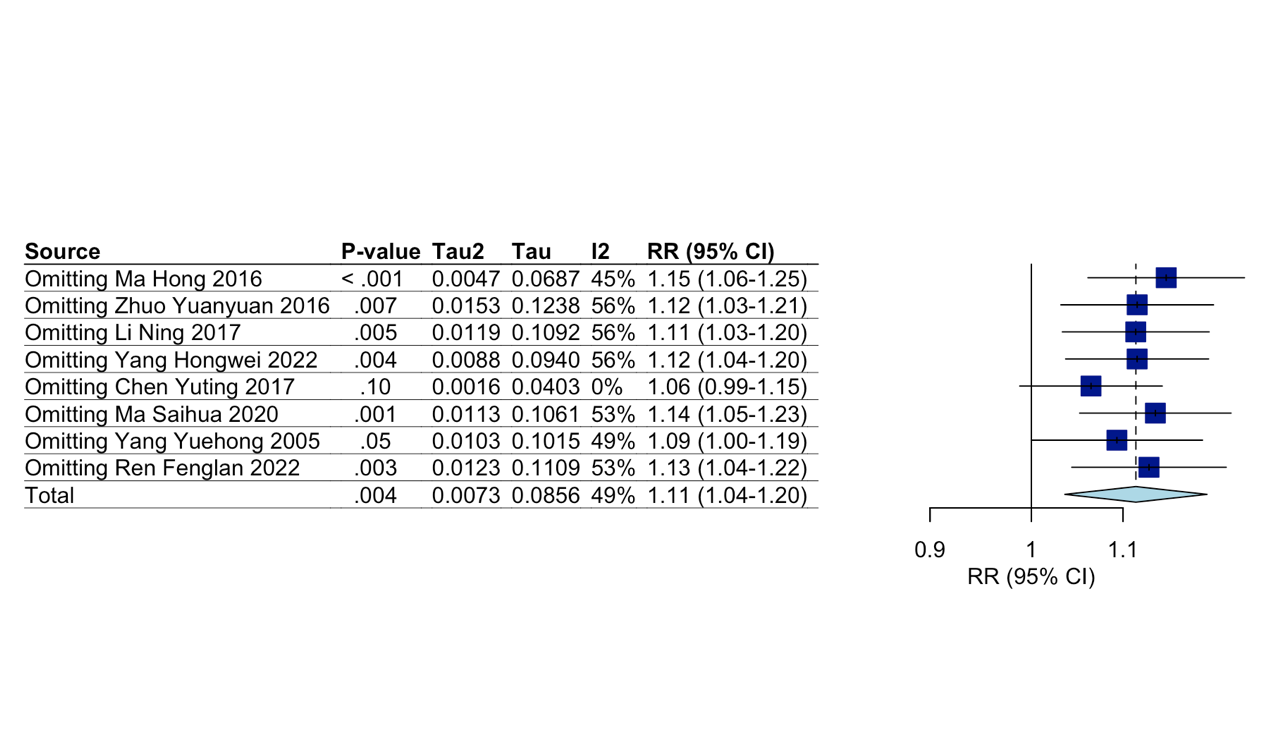
**

**FIGURE7. 3. Sensitivity analysis for ovulation rate in acupuncture versus blank**

**
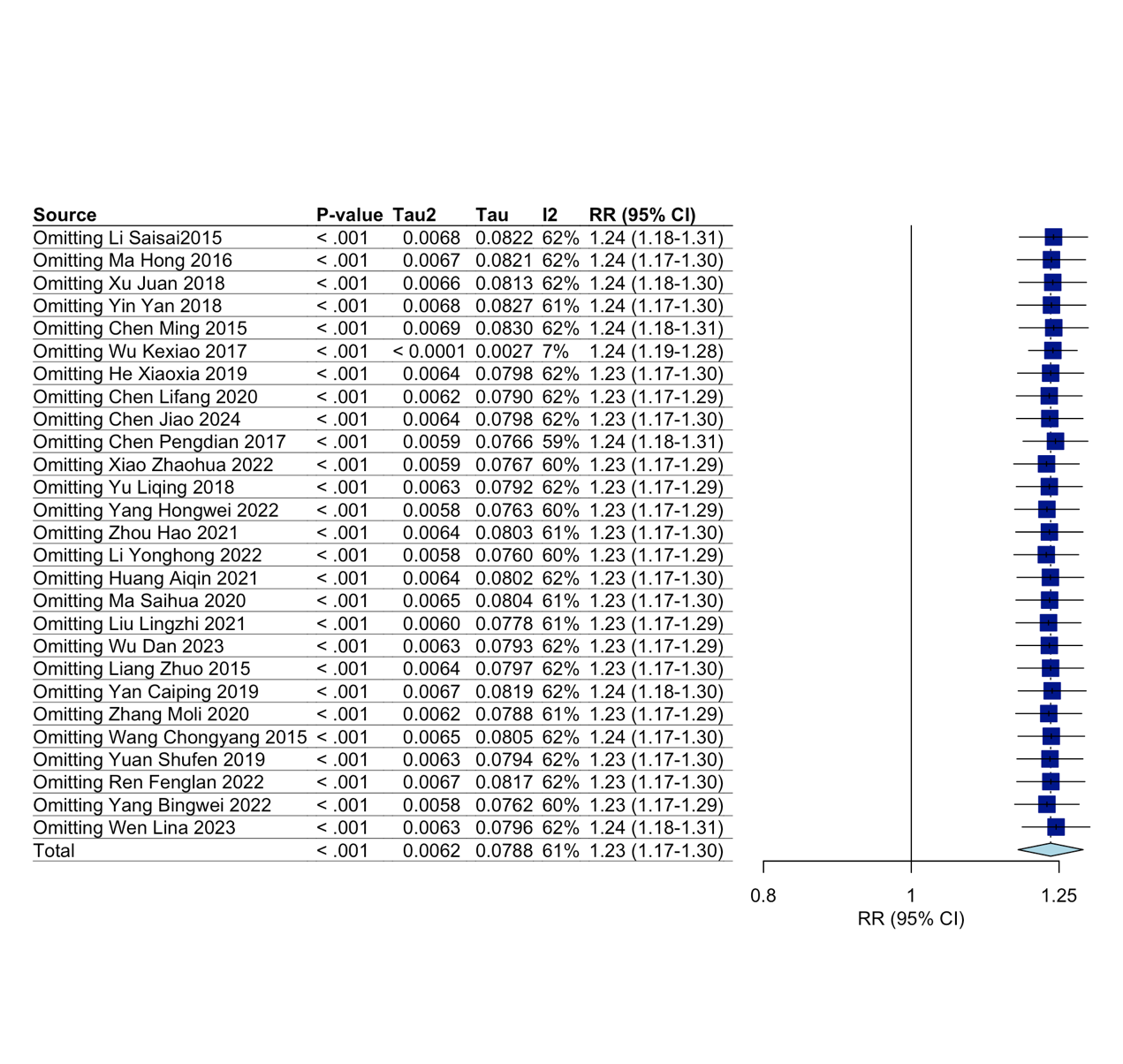
**

**FIGURE7. 4 Sensitivity analysis for ovulation rate in acupuncture and Chinese medicine versus Western medicine**

**
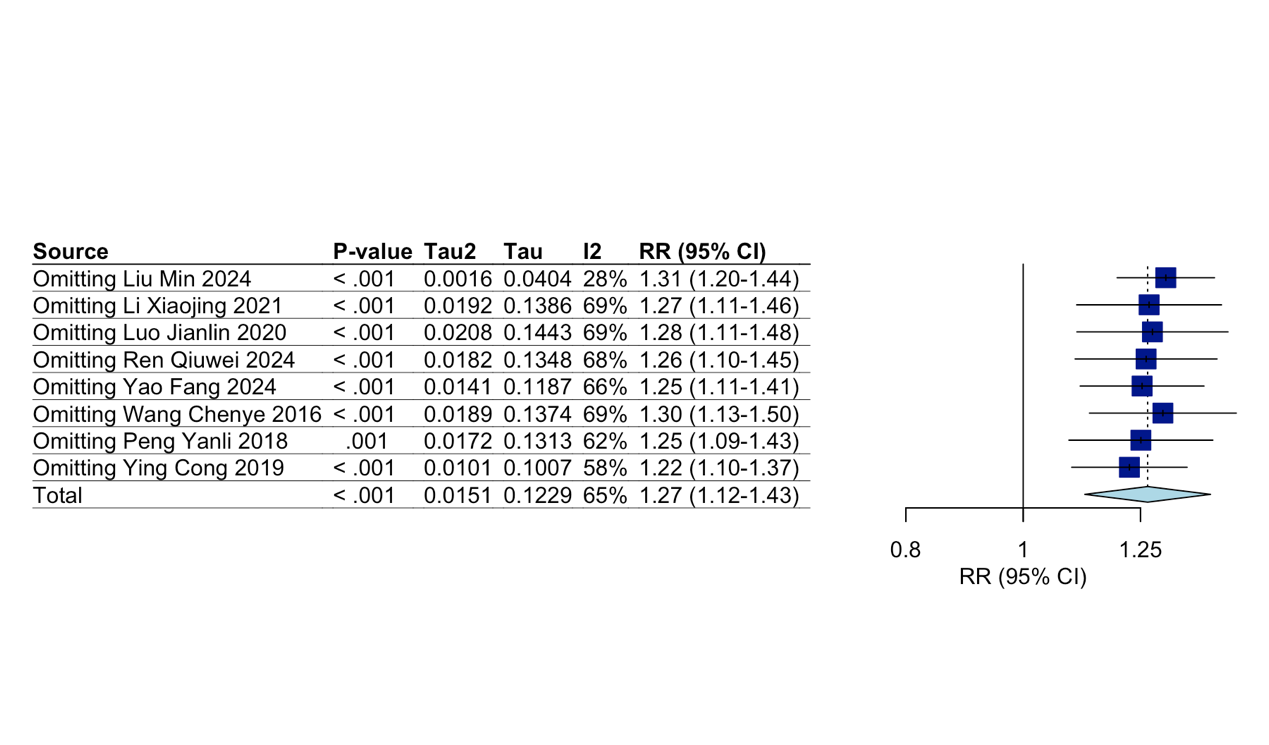
**

**FIGURE7. 5. Sensitivity analysis for BMI in acupuncture versus blank**

**
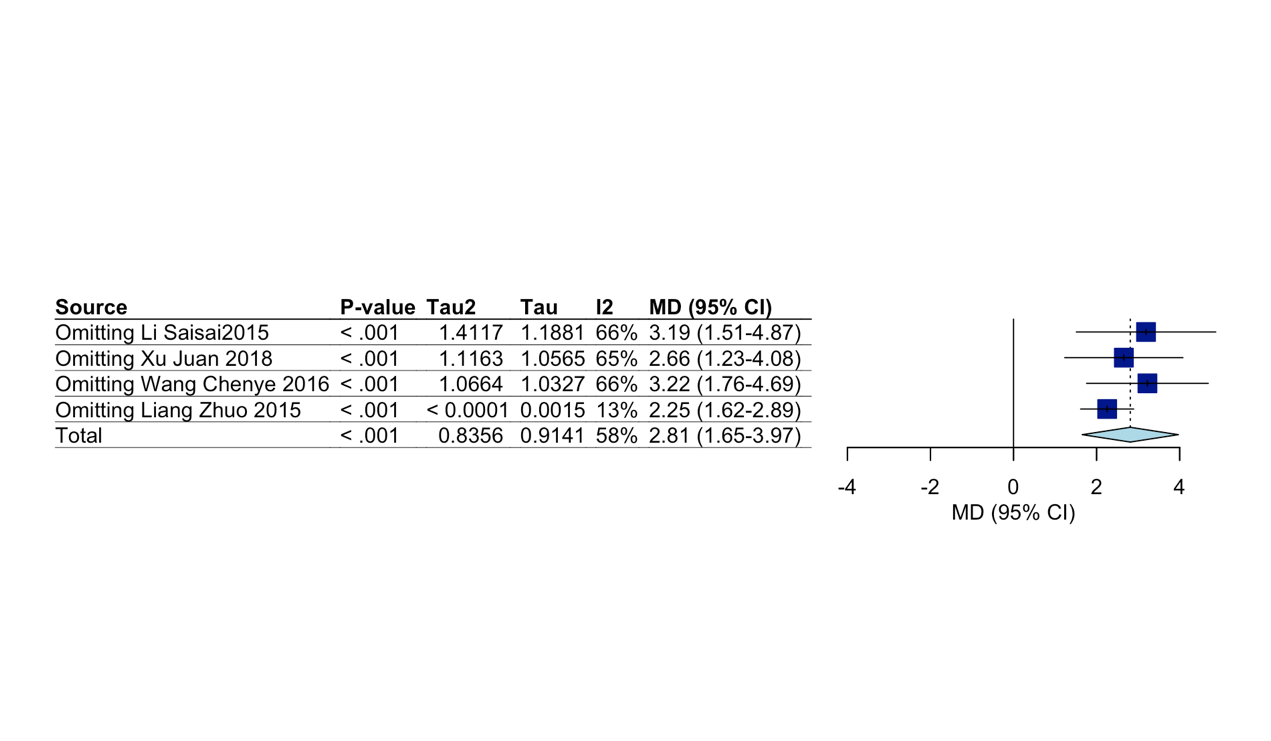
**

**FIGURE7. 6. Sensitivity analysis for FSH in acupuncture and Chinese medicine versus Western medicine**

**
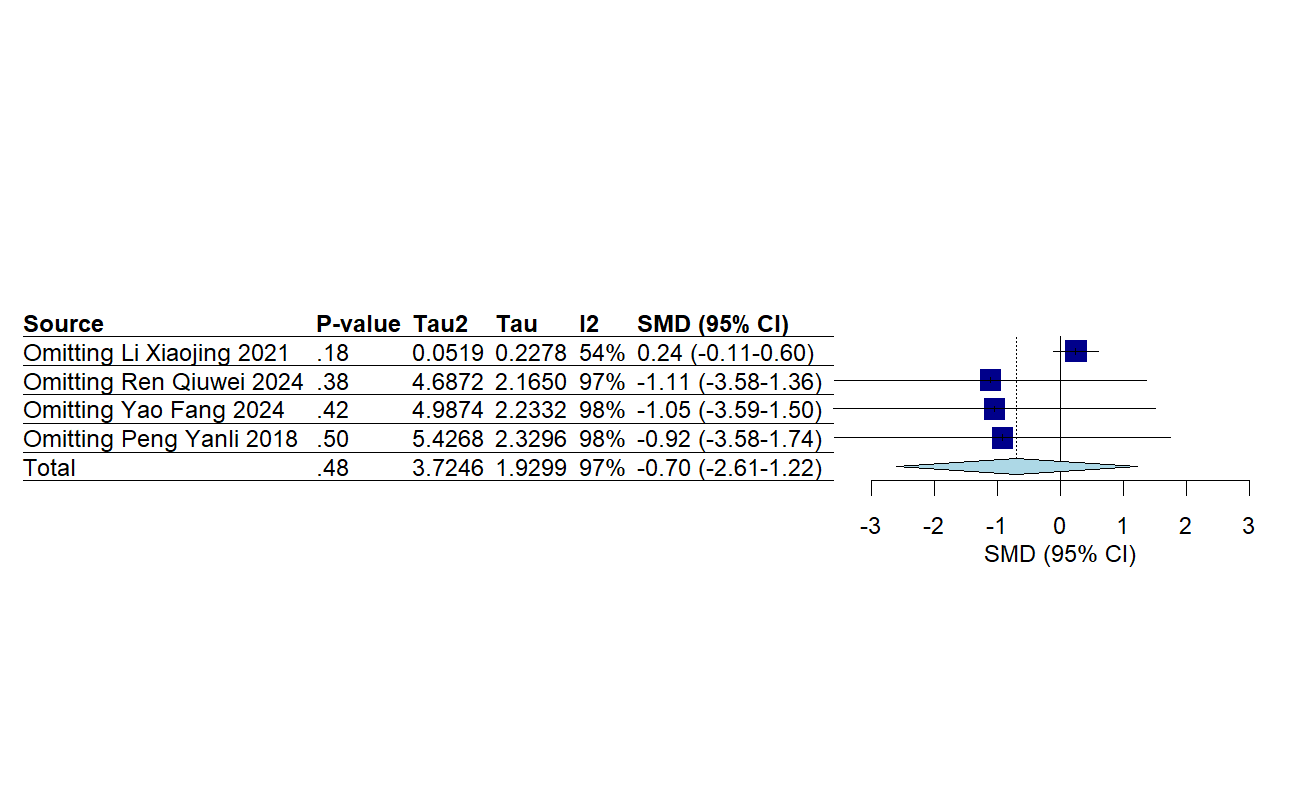
**

**FIGURE7. 7. Sensitivity analysis for FSH in acupuncture versus medicine**

**
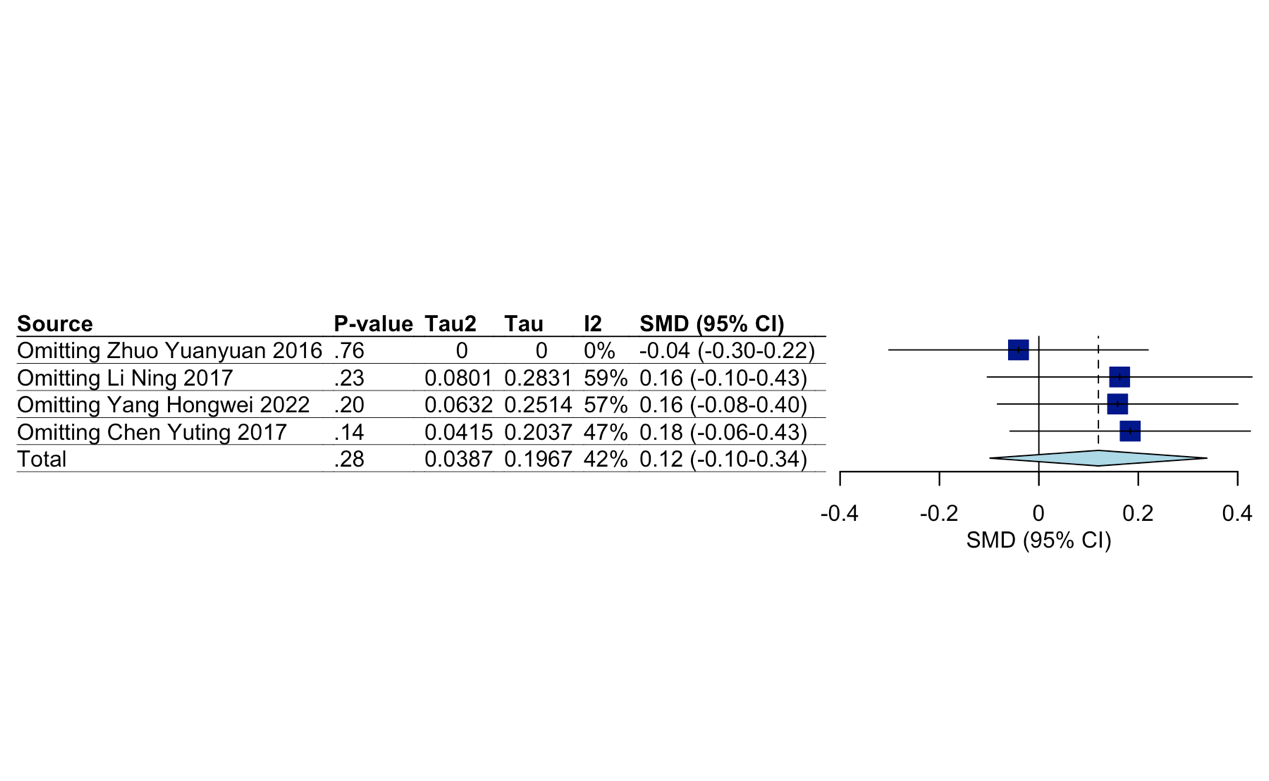
**

**FIGURE7. 8. Sensitivity analysis for FSH in acupuncture versus sham acupuncture**

**
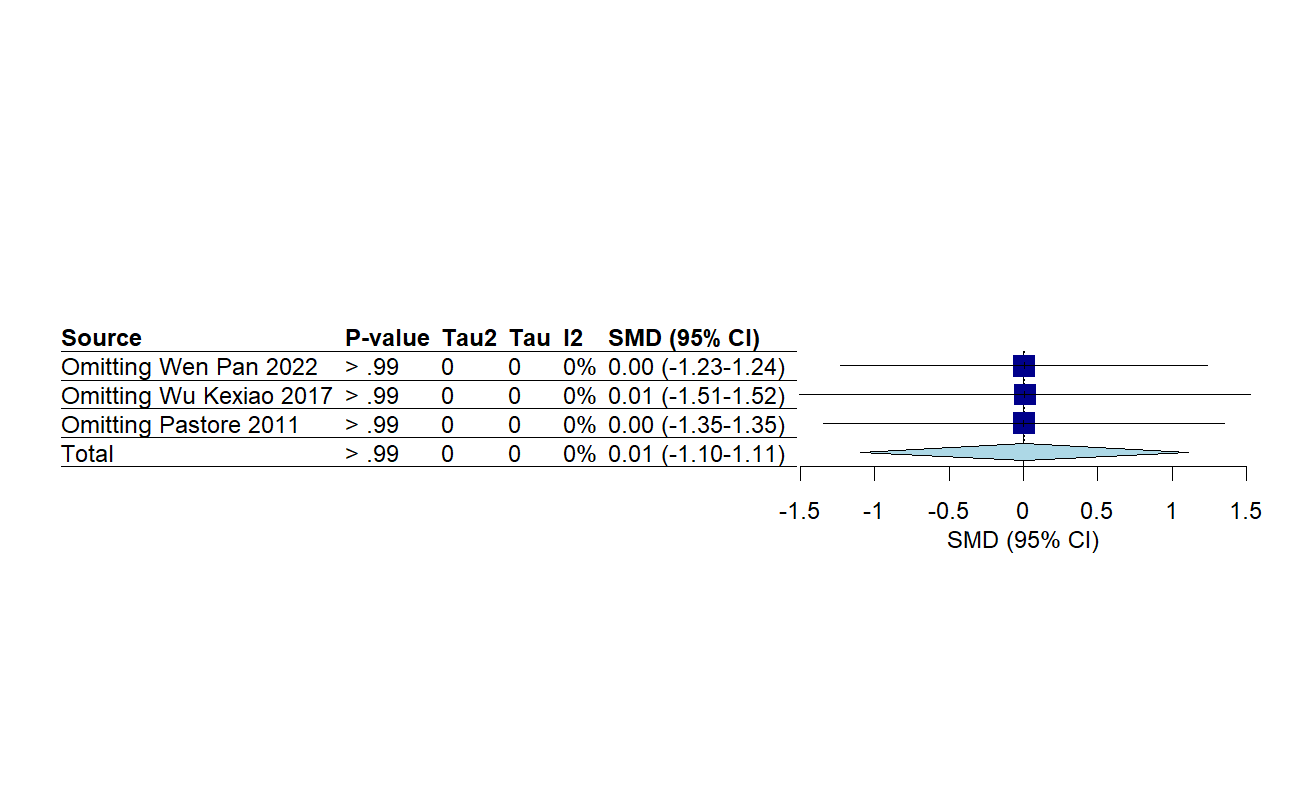
**

**FIGURE7. 9. Sensitivity analysis for FSH in acupuncture versus blank**

**
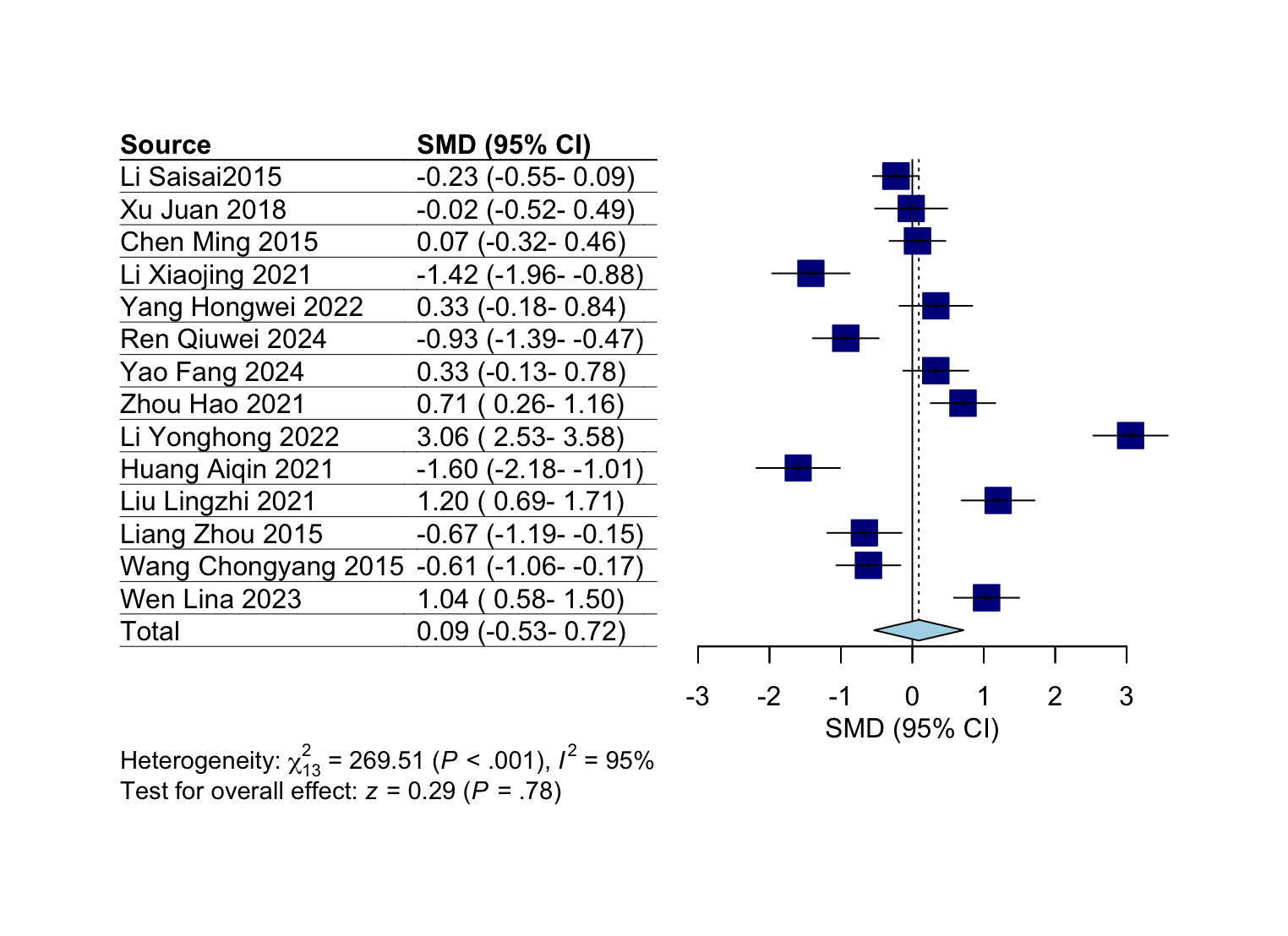
**

**FIGURE7. 10. Sensitivity analysis for LH in acupuncture versus medicine**

**
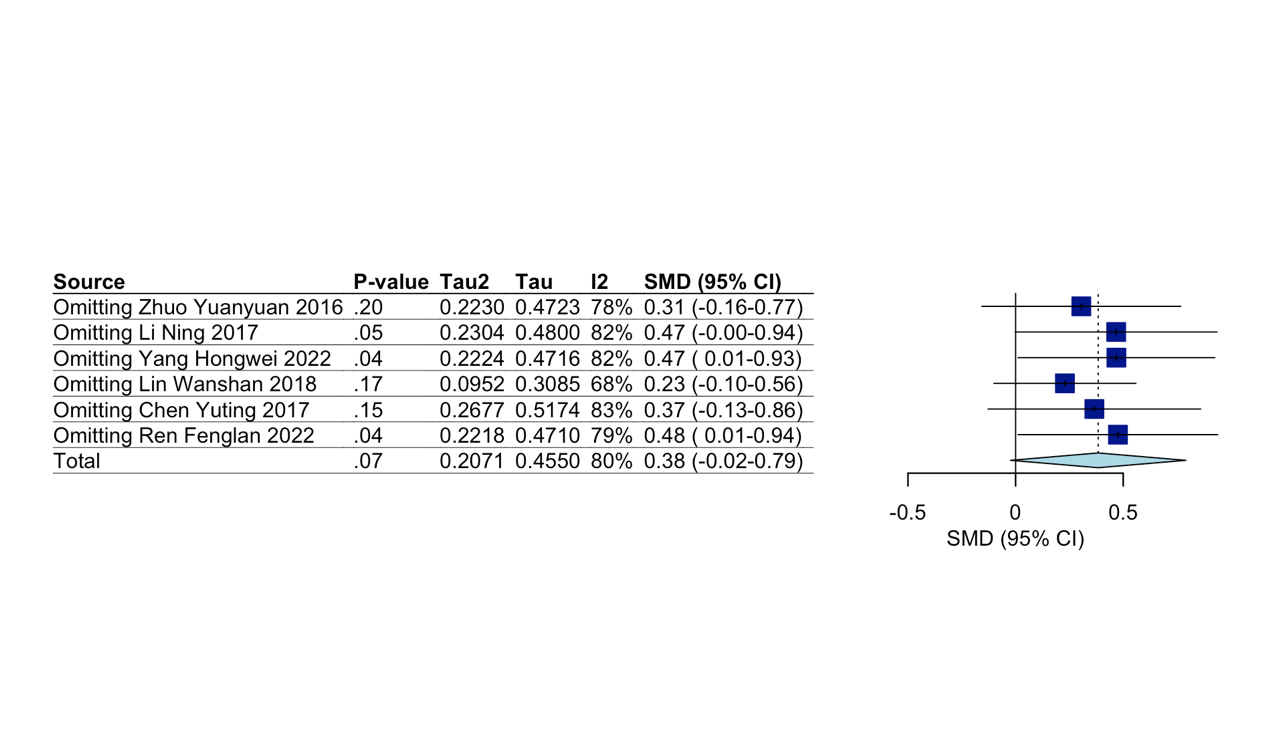
**

**FIGURE7. 11. Sensitivity analysis for LH in acupuncture and Chinese medicine versus western medicine**

**
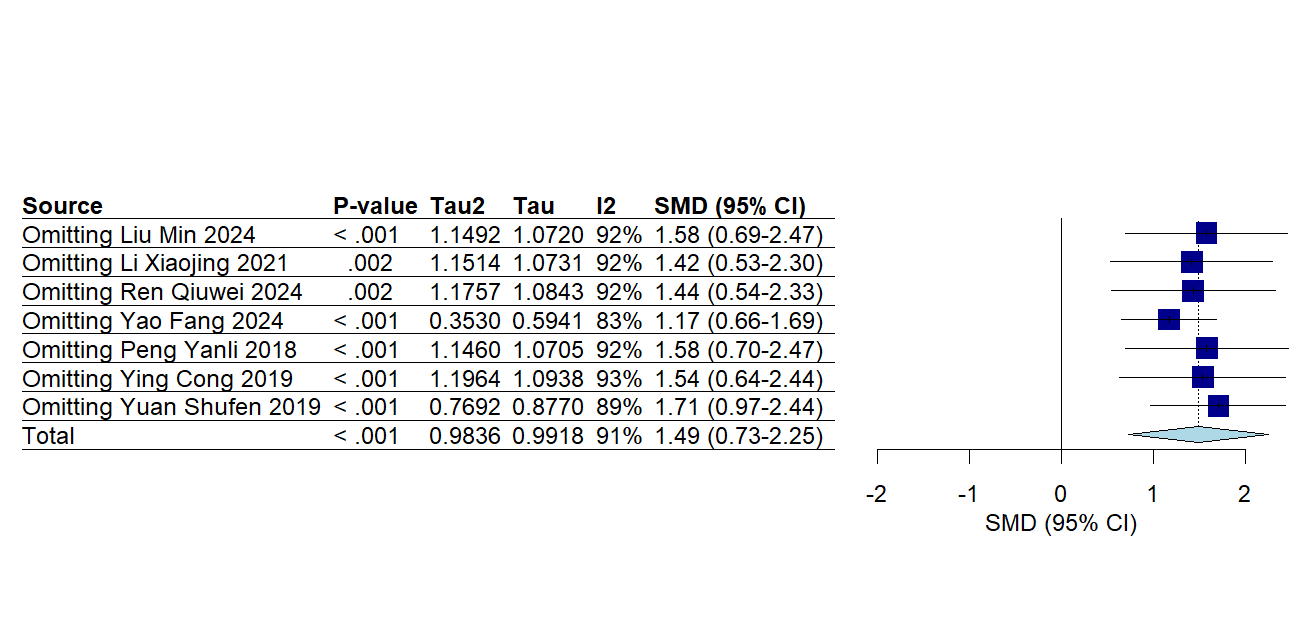
**

**FIGURE7. 12. Sensitivity analysis for LH in acupuncture versus sham acupuncture**

**
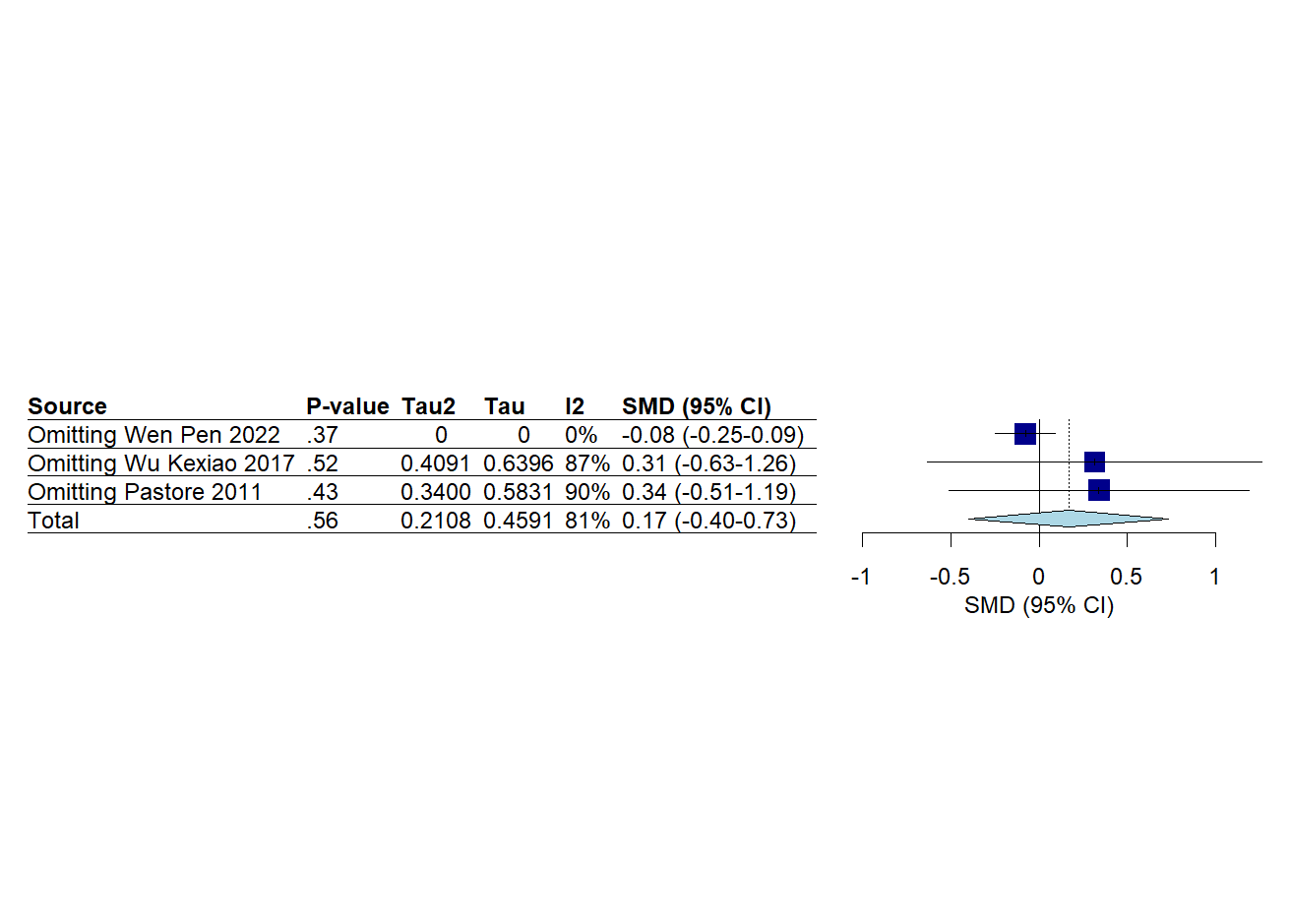
**

**FIGURE7. 13. Sensitivity analysis for LH in acupuncture versus blank**

**
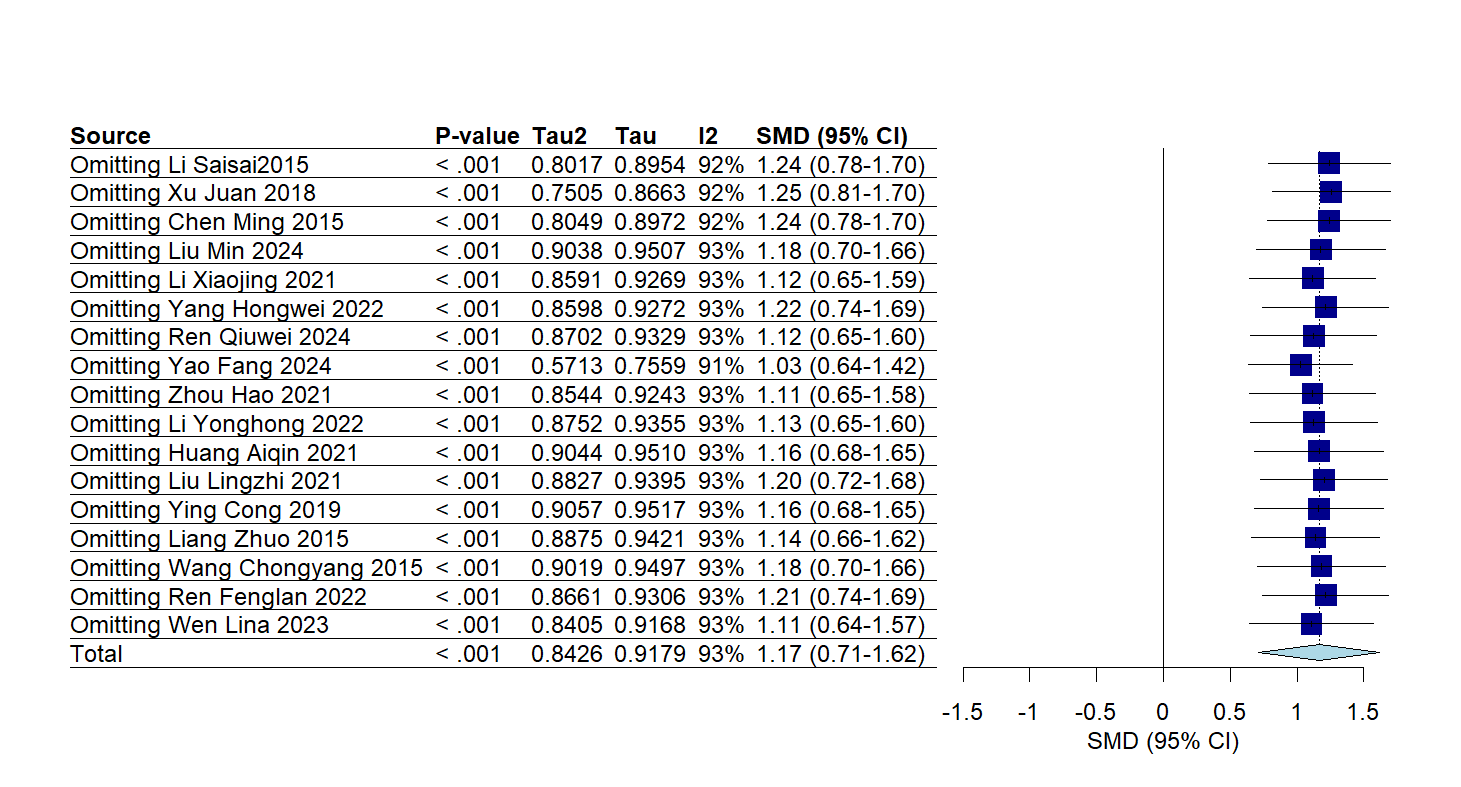
**

**FIGURE7. 14. Sensitivity analysis for LH:FSH ratio in acupuncture and Chinese medicine versus Western medicine**

**
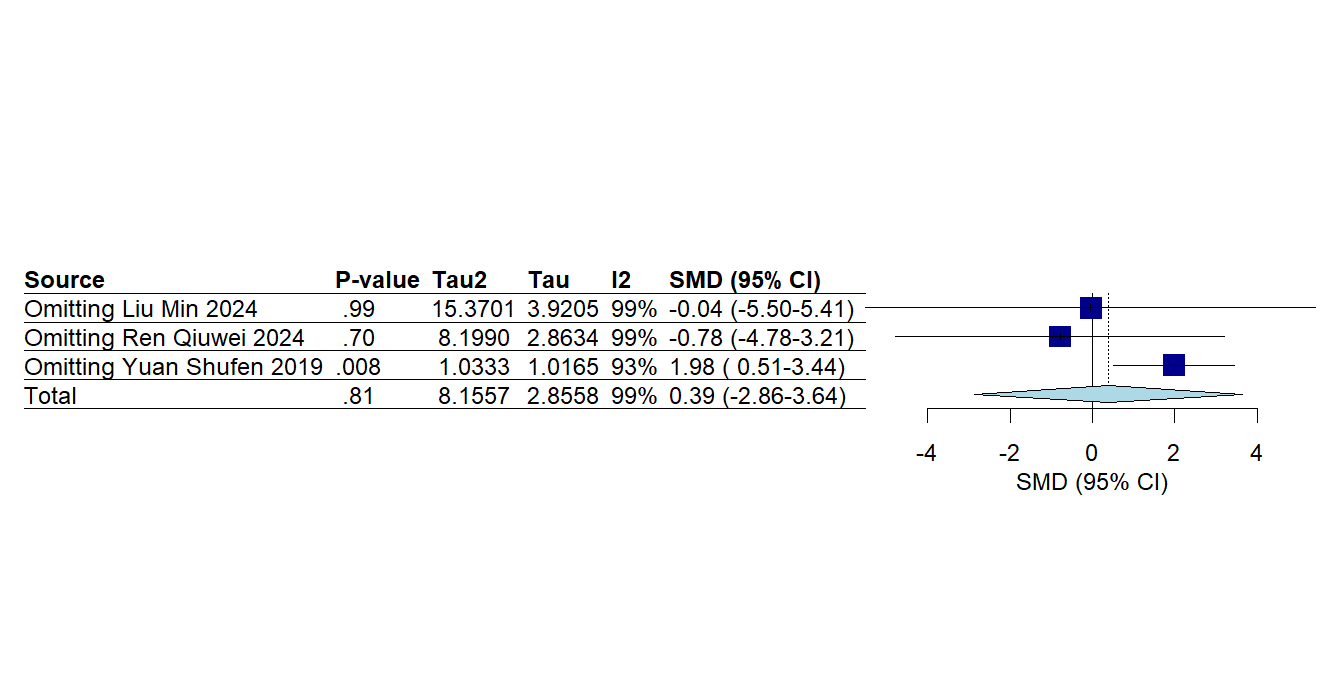
**

**FIGURE7. 15. Sensitivity analysis for LH:FSH ratio in acupuncture versus blank**

**
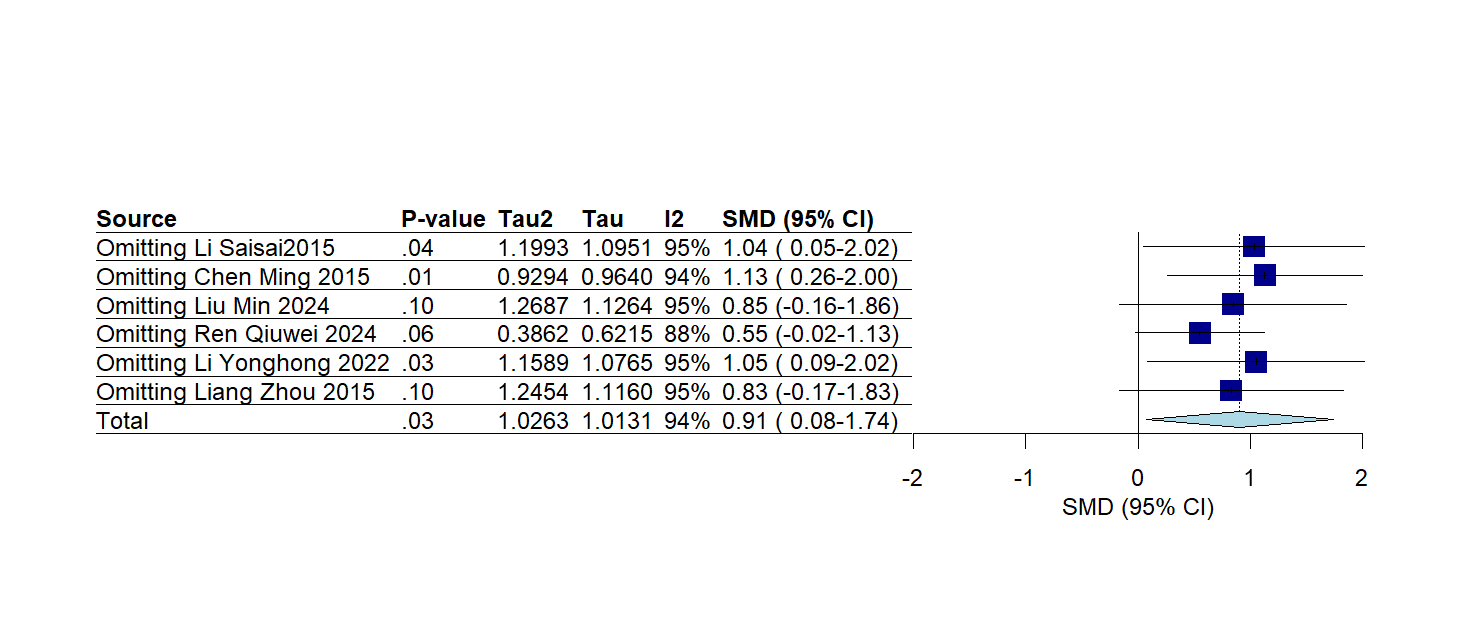
**

**FIGURE7. 16. Sensitivity analysis for T in acupuncture versus medicine**

**
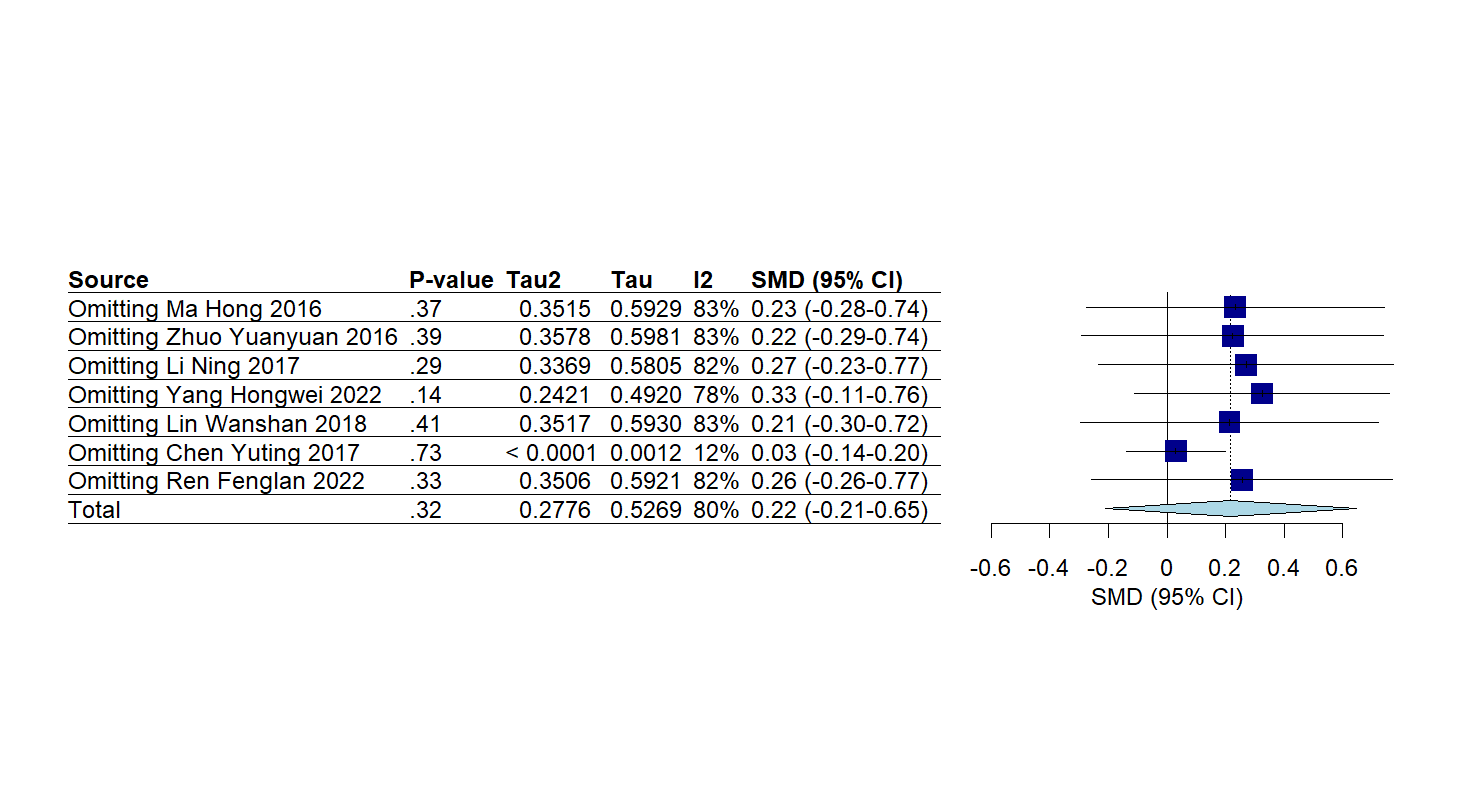
**

**FIGURE7. 17. Sensitivity analysis for T in acupuncture and Chinese medicine versus western medicine**

**
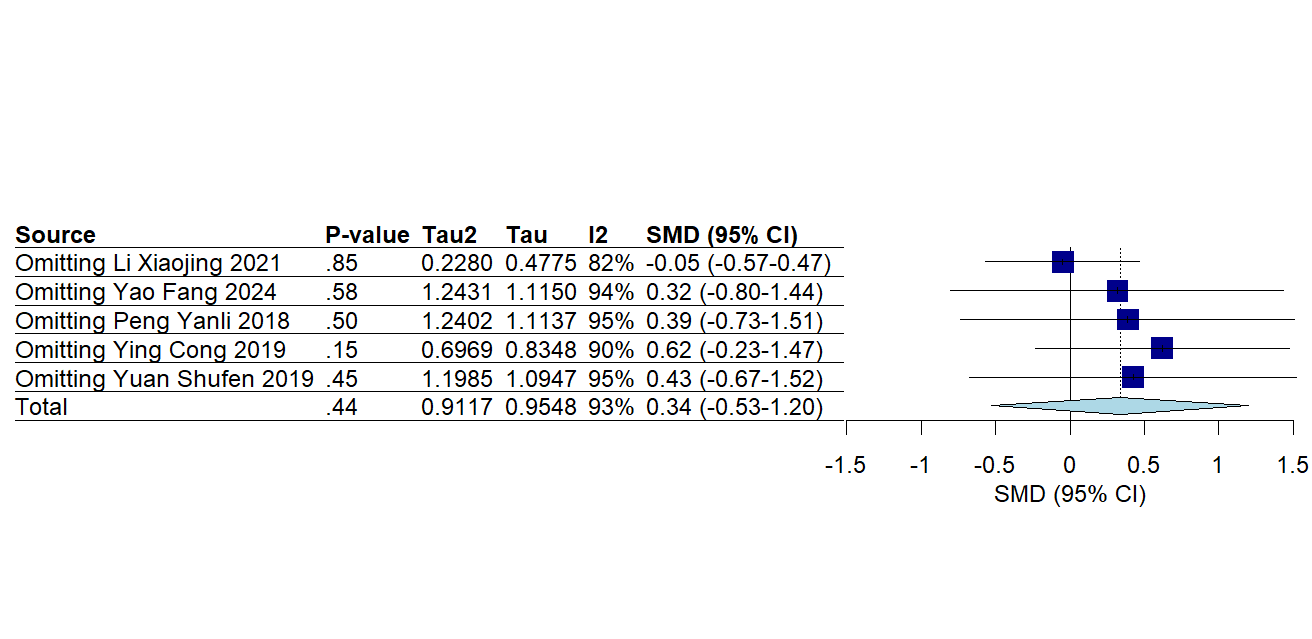
**

**FIGURE7. 18. Sensitivity analysis for T in acupuncture versus blank**

**
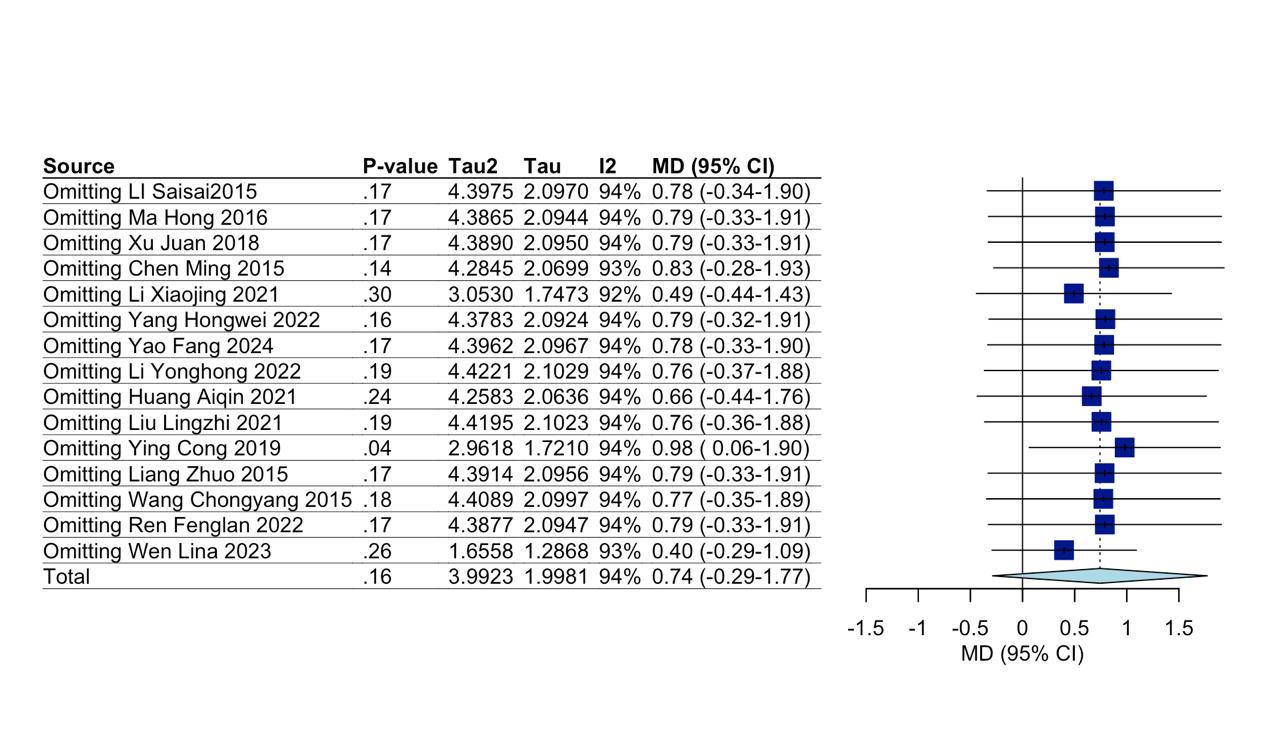
**

1. **Supplemental file 8. Subgroup analysis of Pairwise Meta-Analysis**

**FIGURE8. 1. Forest plot of the subgroup analyses for ovulation ratio in acupuncture and Chinese medicine versus Western medicine with different acupuncture dose**

**
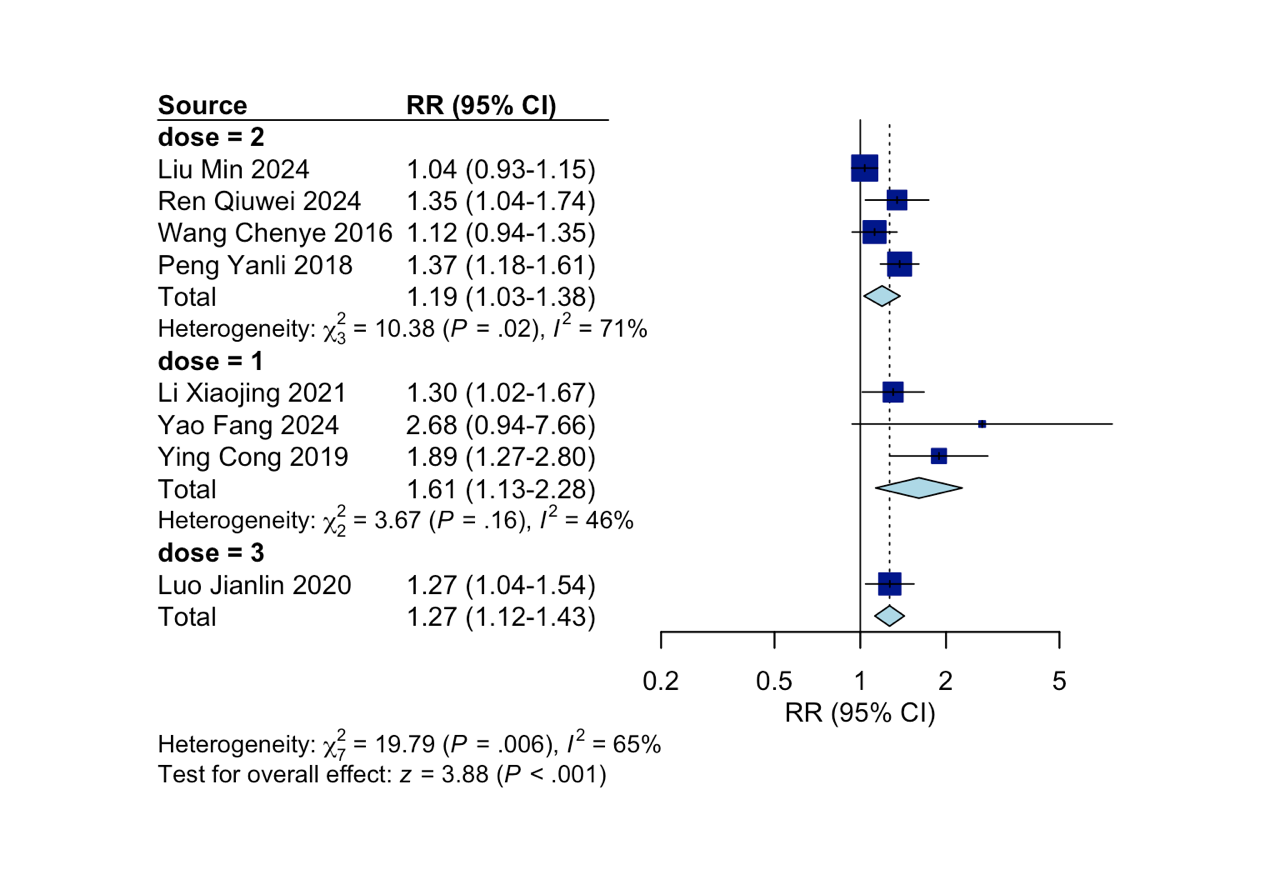
**

**FIGURE8. 2. Forest plot of the subgroup analyses for ovulation ratio in acupuncture and Chinese medicine versus Western medicine with different acupuncture type**

**
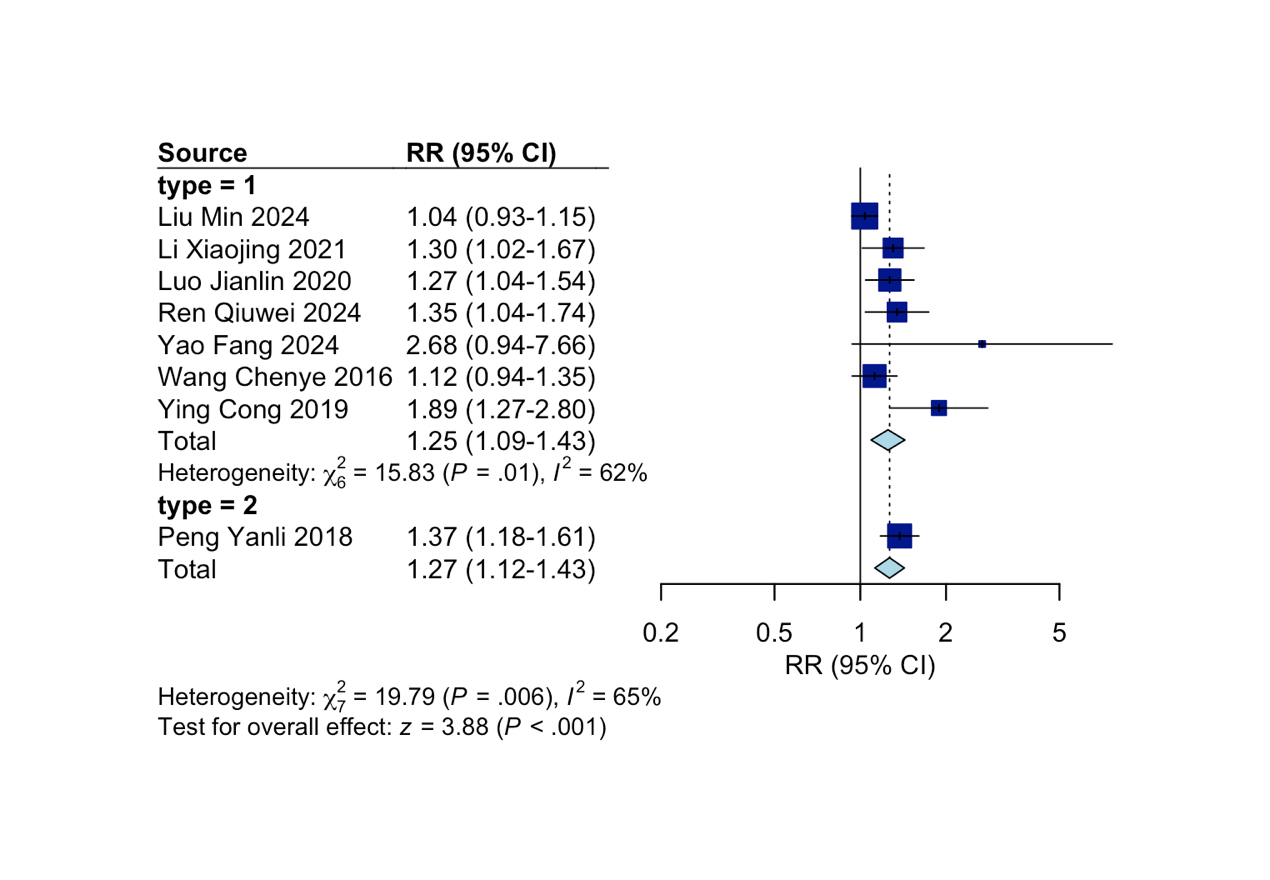
**

**FIGURE8. 3. Forest plot of the subgroup analyses for ovulation ratio in acupuncture and Chinese medicine versus Western medicine with different retaining time**

**
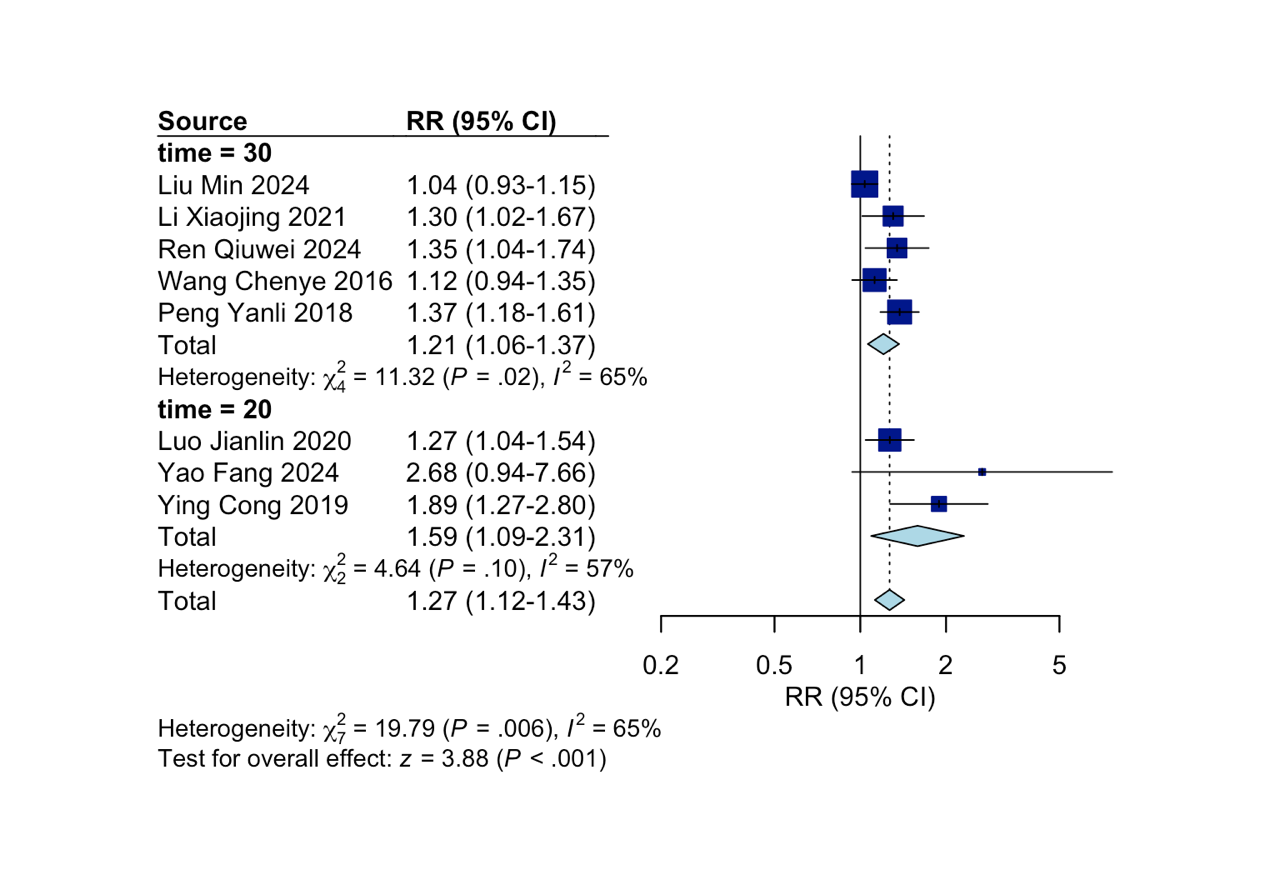
**

**FIGURE8. 4. Forest plot of the subgroup analyses for ovulation ratio in acupuncture and Chinese medicine versus Western medicine with different acupuncture frequency**

**
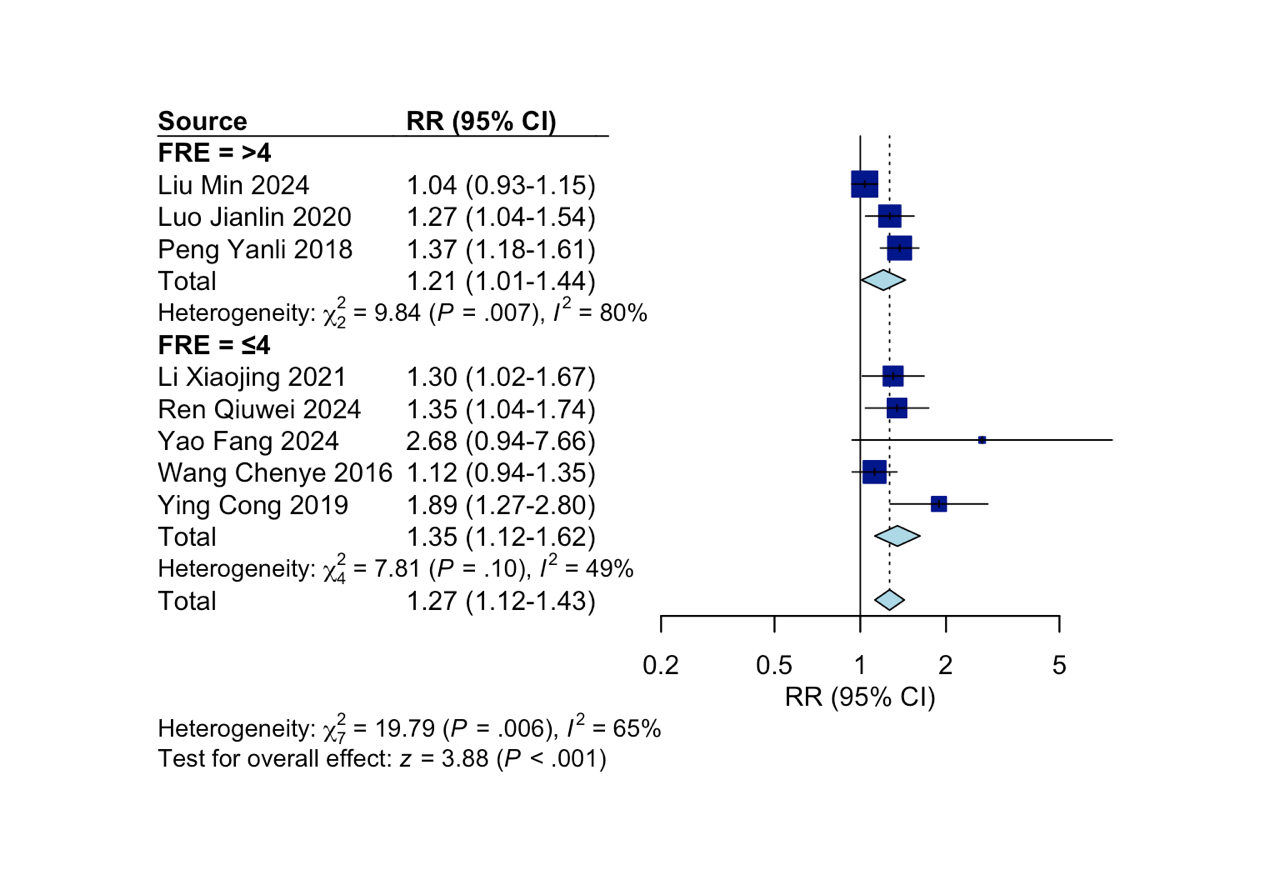
**

**FIGURE8. 5. Forest plot of the subgroup analyses for ovulation ratio in acupuncture and Chinese medicine versus Western medicine with different acupoints number**

**
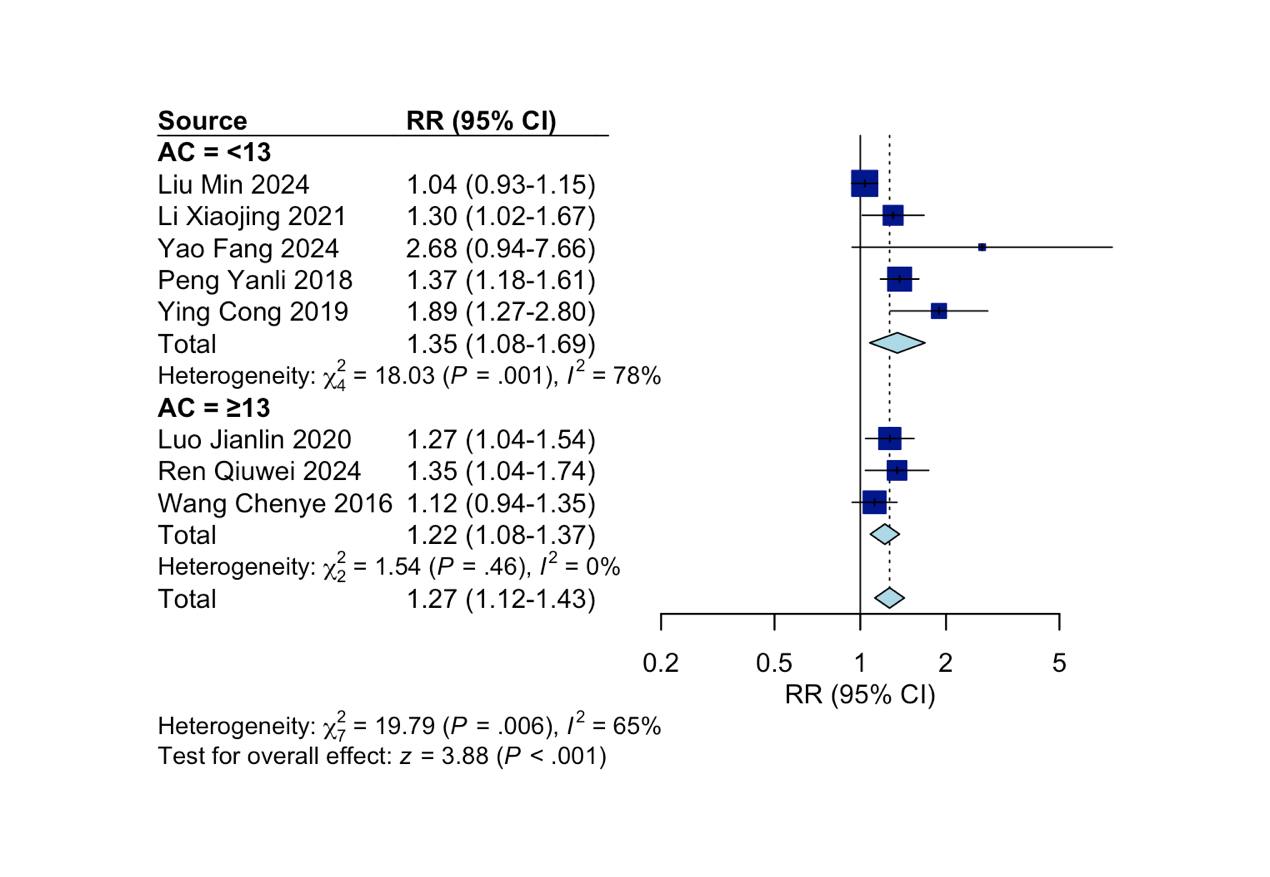
**

**FIGURE8. 6.Forest plot of the subgroup analyses for ovulation ratio in acupuncture versus blank with different acupuncture dose**

**
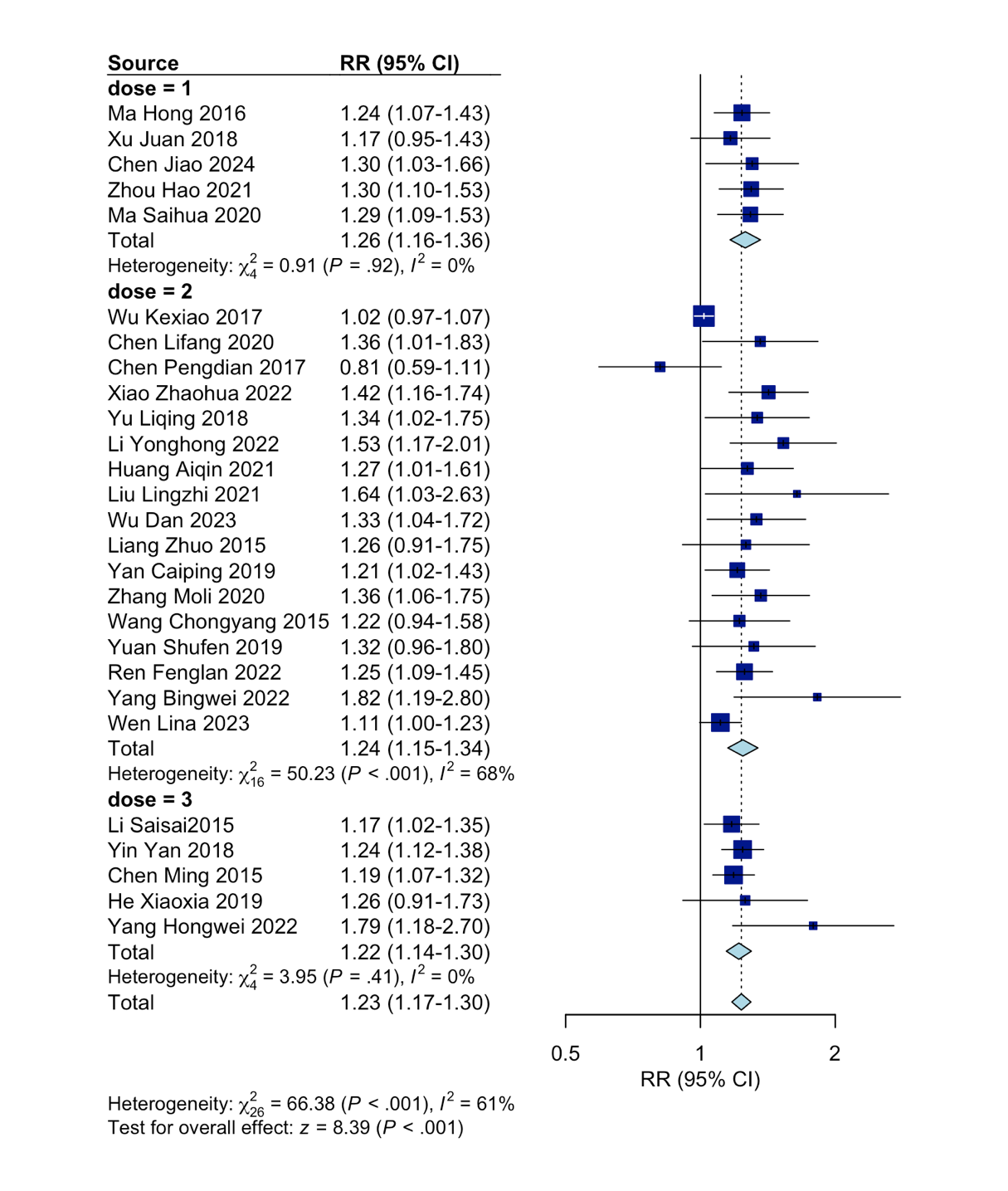
**

**FIGURE8. 7. Forest plot of the subgroup analyses for ovulation ratio in acupuncture versus blank with different acupuncture type**

**
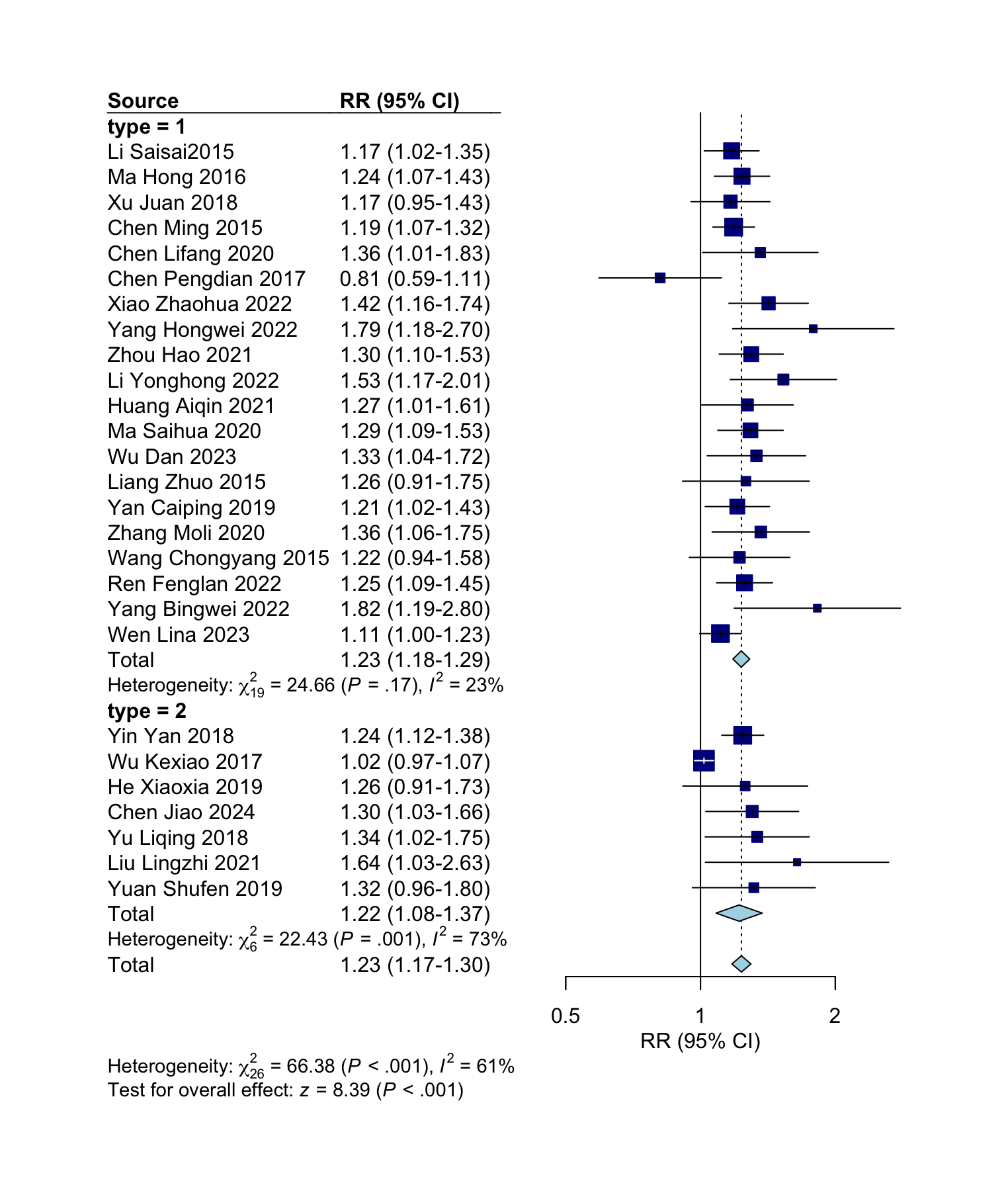
**

**FIGURE8. 8. Forest plot of the subgroup analyses for ovulation ratio in acupuncture versus blank with different mean age**

**
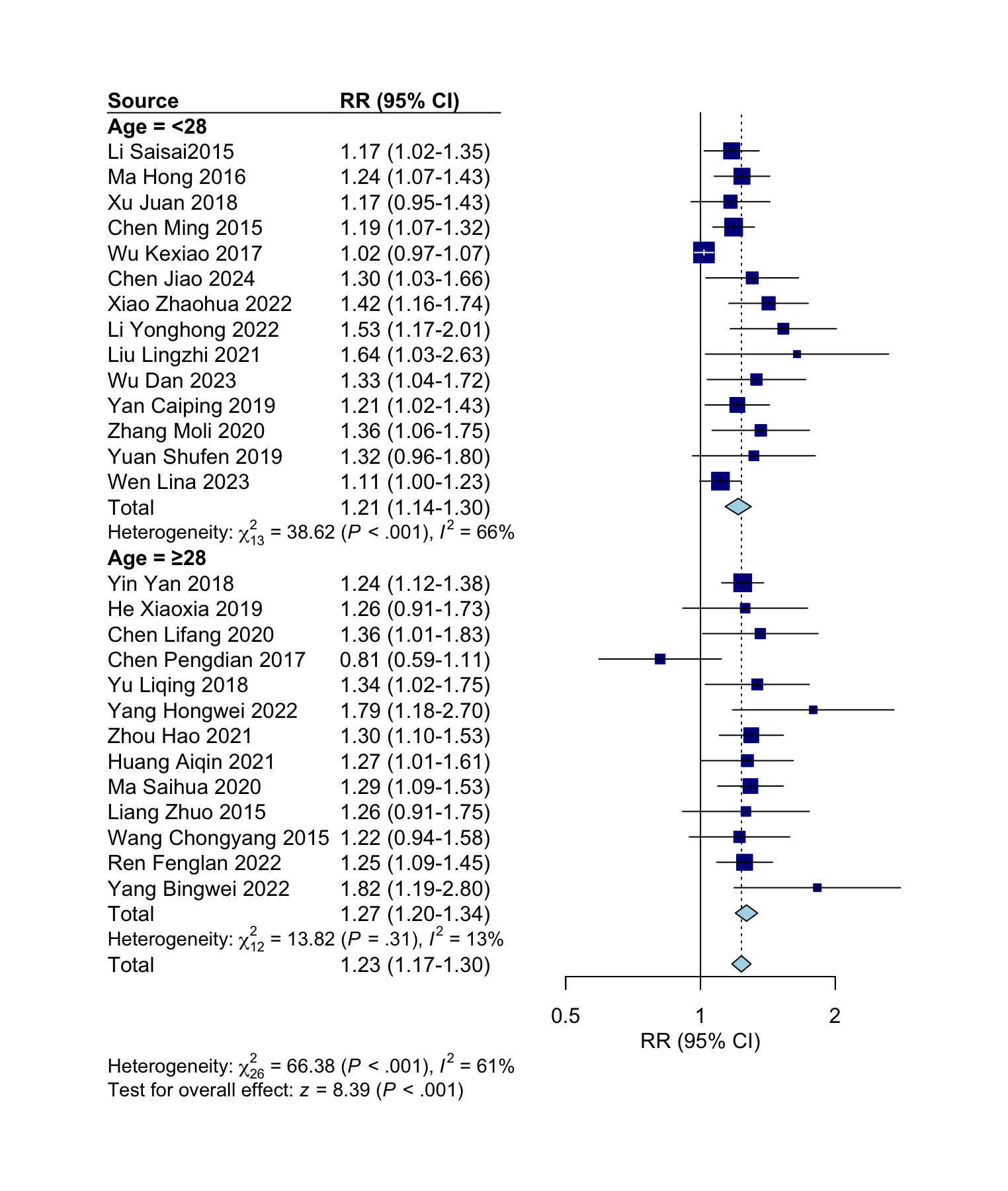
**

**FIGURE8. 9. Forest plot of the subgroup analyses for ovulation ratio in acupuncture versus blank with different acupuncture frequency**

**
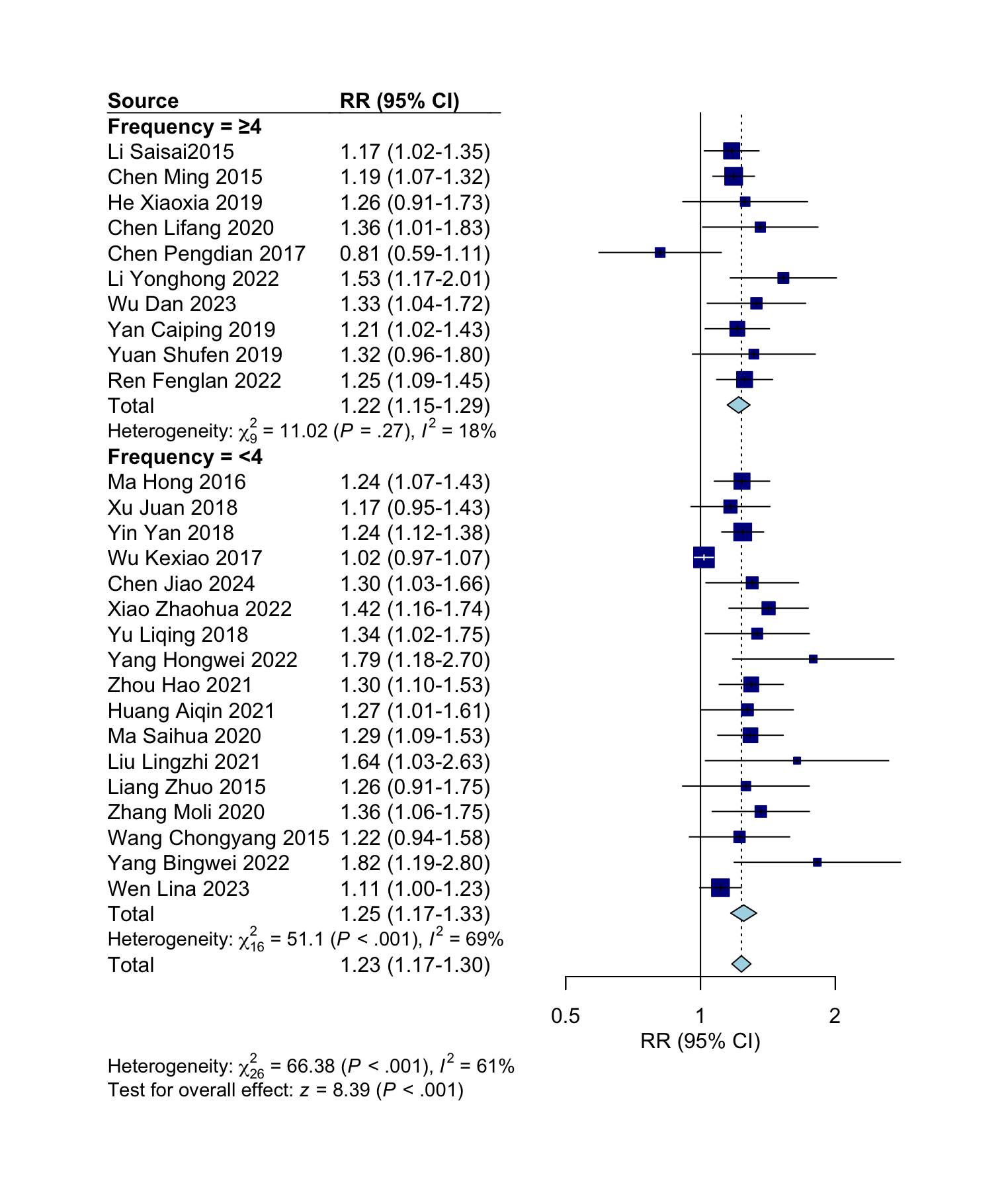
**

**FIGURE8. 10. Forest plot of the subgroup analyses for ovulation ratio in acupuncture versus blank with different acupoint number**

**
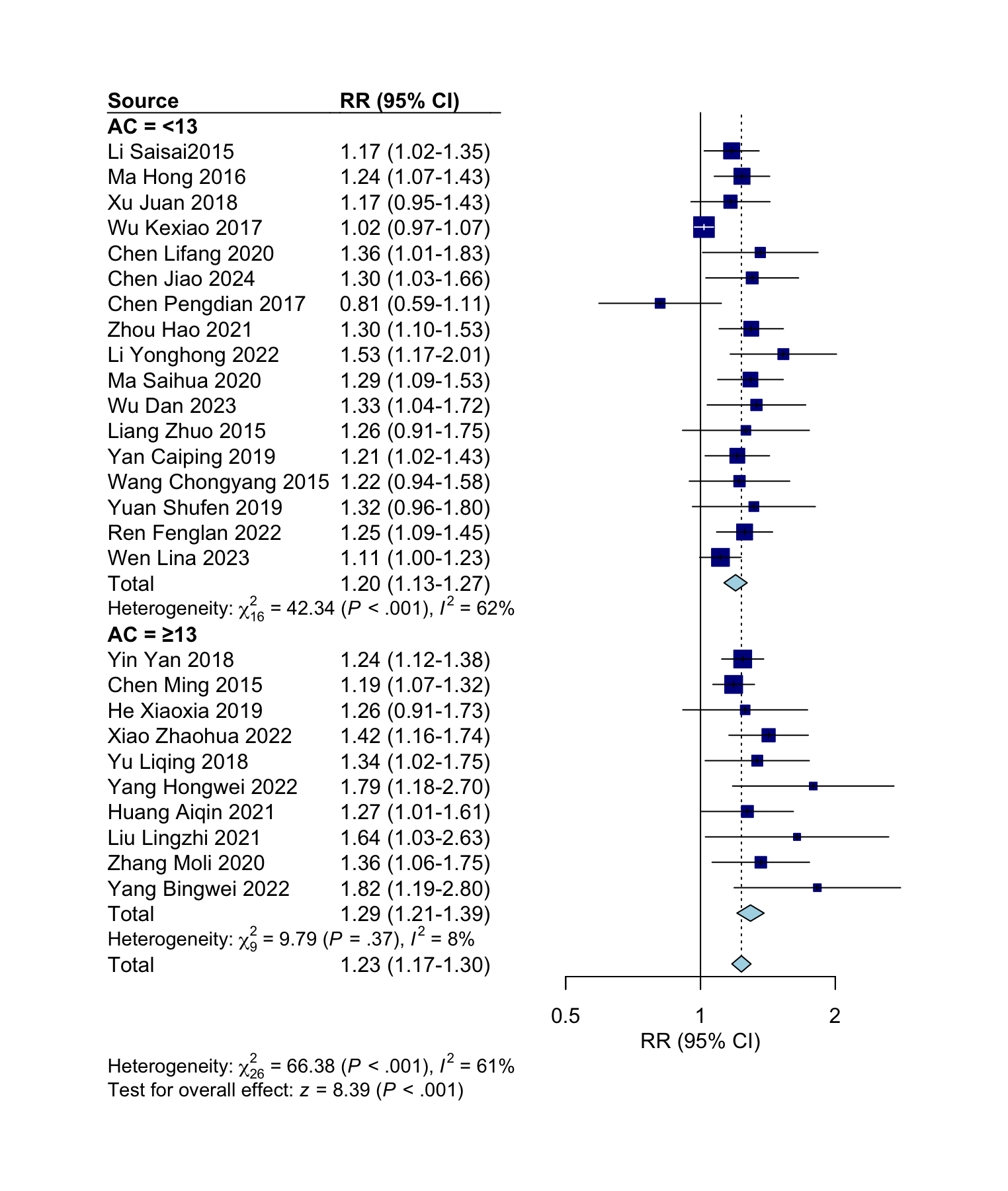
**

FIGURE8. 11. **Forest plot of the subgroup analyses for ovulation ratio in acupuncture versus medicine with different acupuncture dose**

**
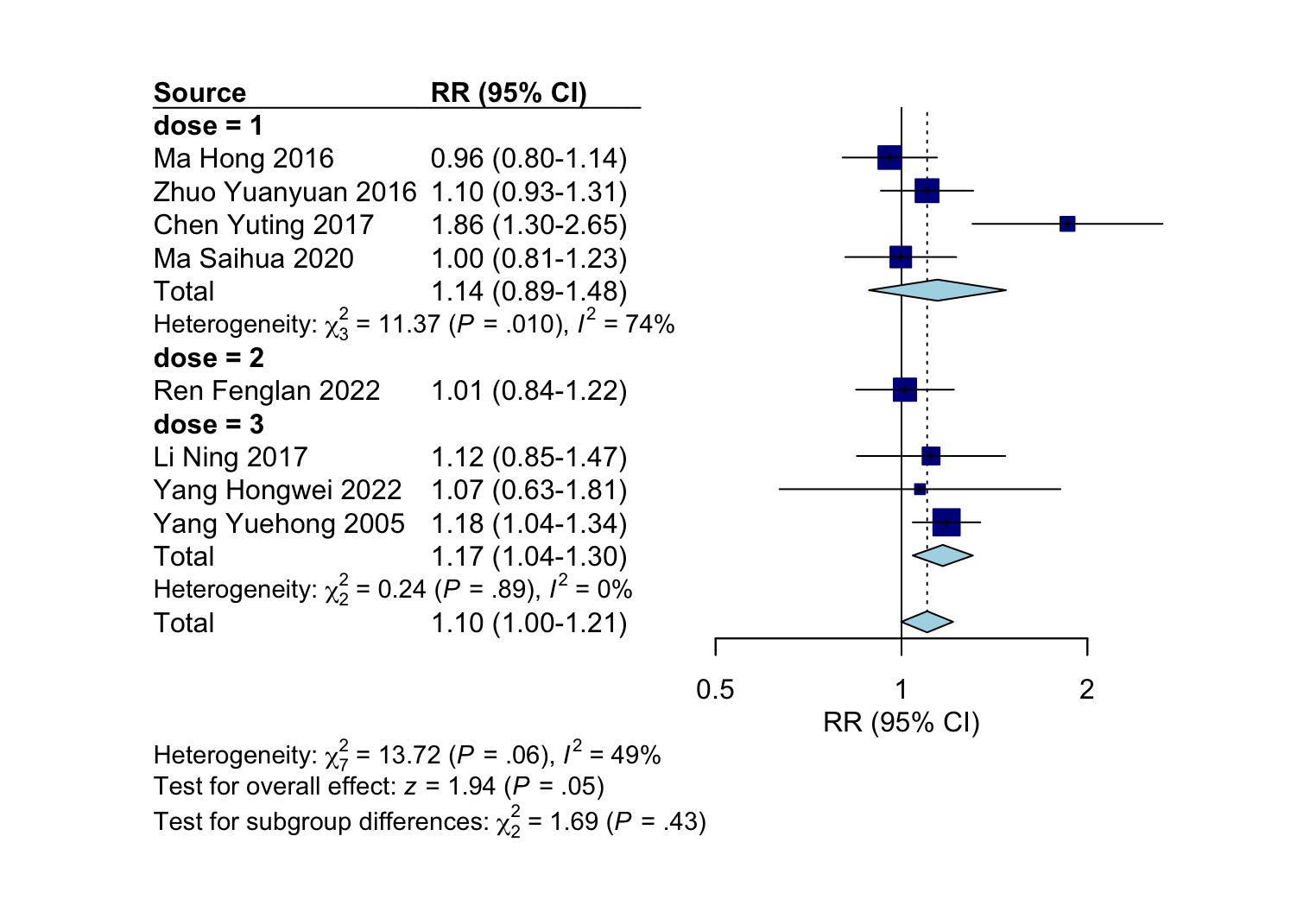
**

**FIGURE8. 12. Forest plot of the subgroup analyses for ovulation ratio in acupuncture versus medicine with different mean age**

**
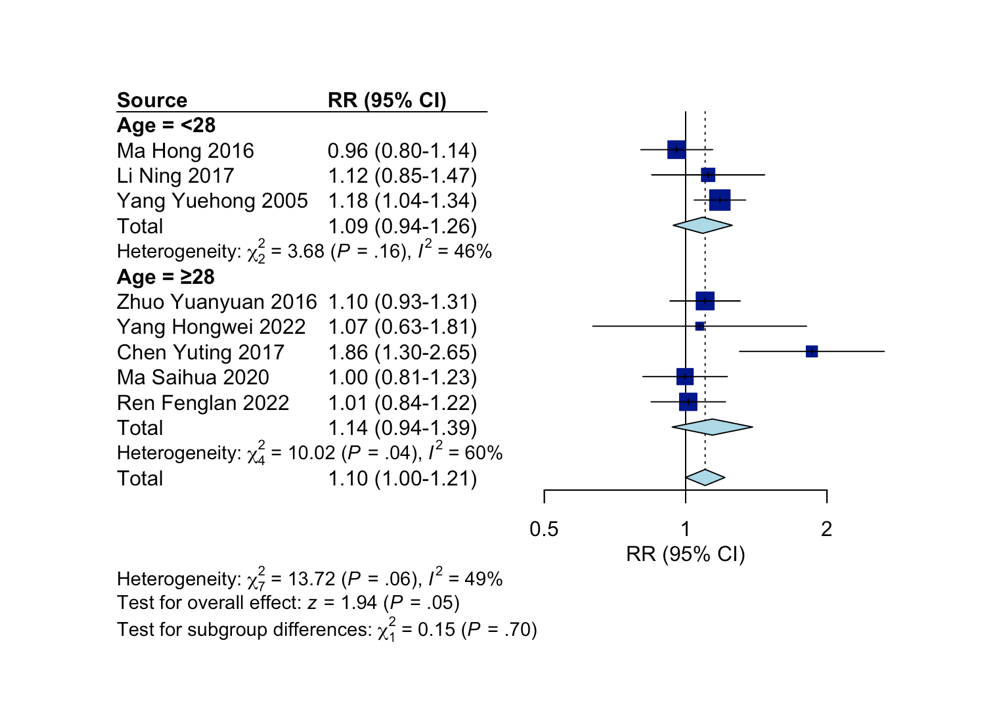
**

**FIGURE8. 13. Forest plot of the subgroup analyses for ovulation ratio in acupuncture versus medicine with different acupuncture frequency**

**
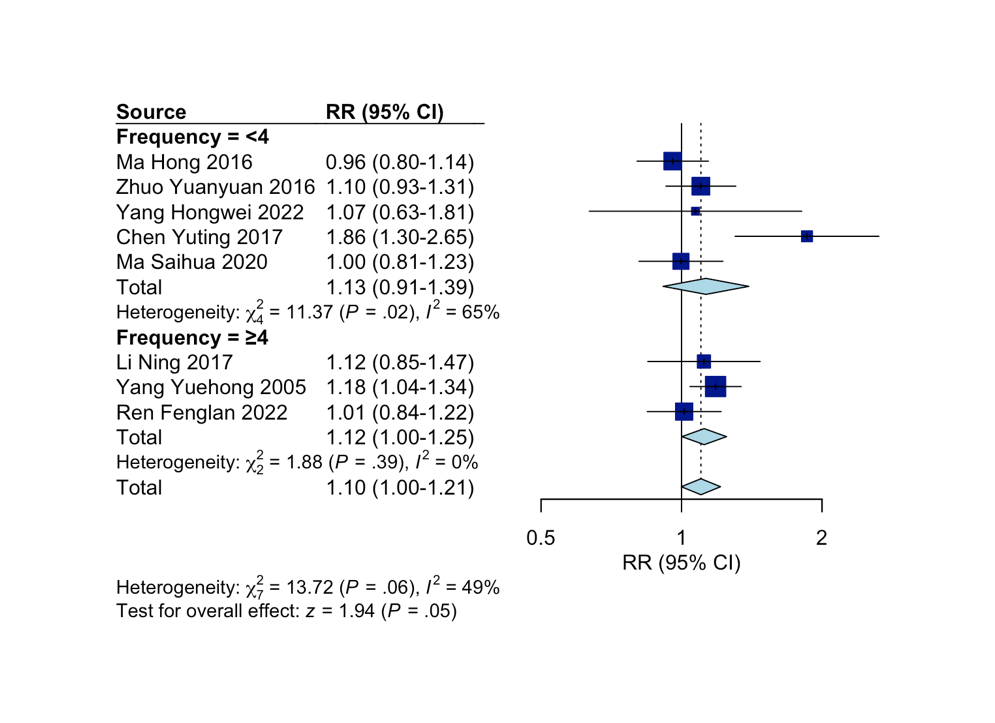
**

**FIGURE8. 14. Forest plot of the subgroup analyses for ovulation ratio in acupuncture versus medicine with different acupoint number**

**
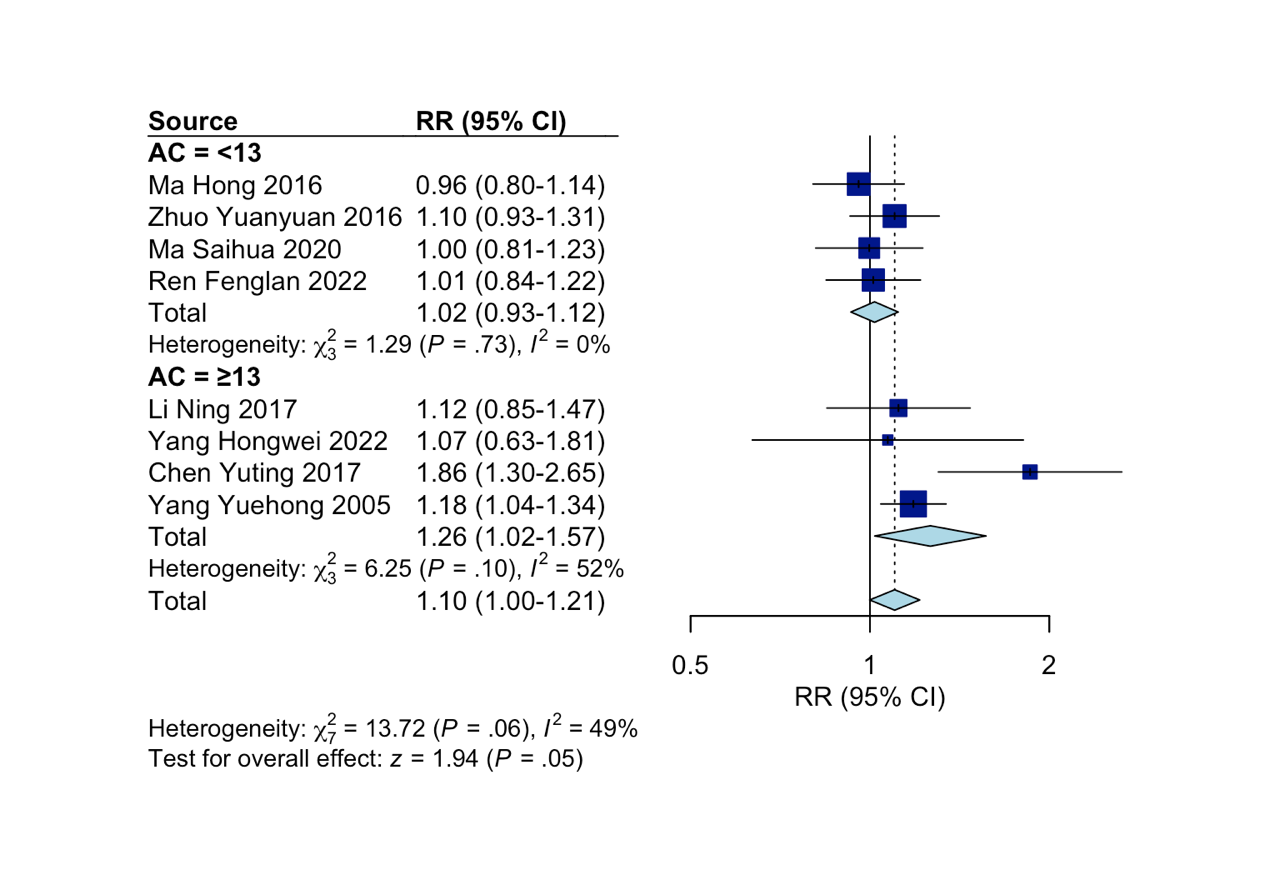
**

1. **Supplemental file 9. Histogram plot of the network meta-analyses**

**FIGURE9. 1.** **Histogram plot of the network meta-analyses for ovulation ratio**

**
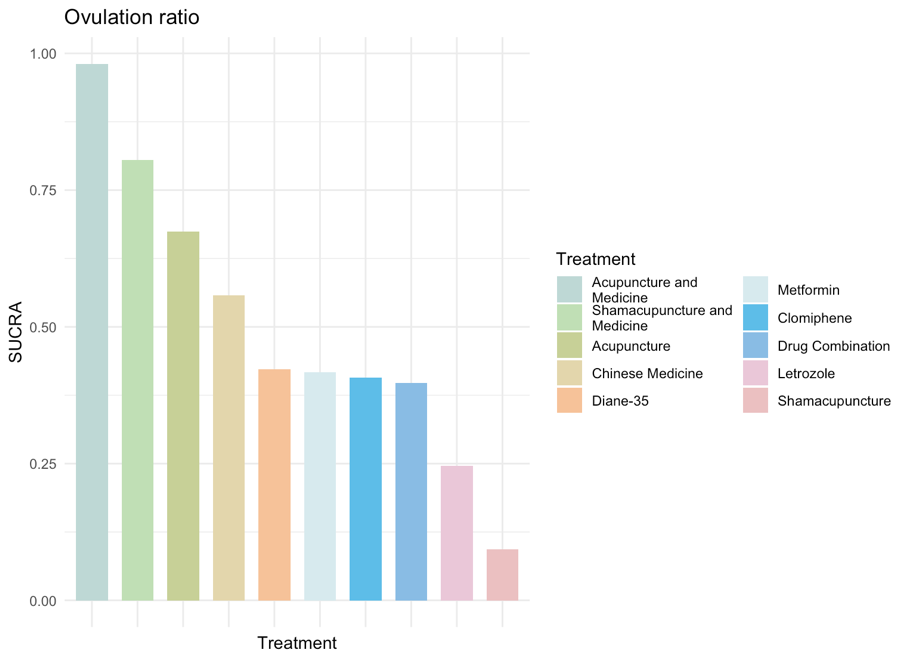
**

**FIGURE9. 2. Histogram plot of the network meta-analyses for BMI**

**
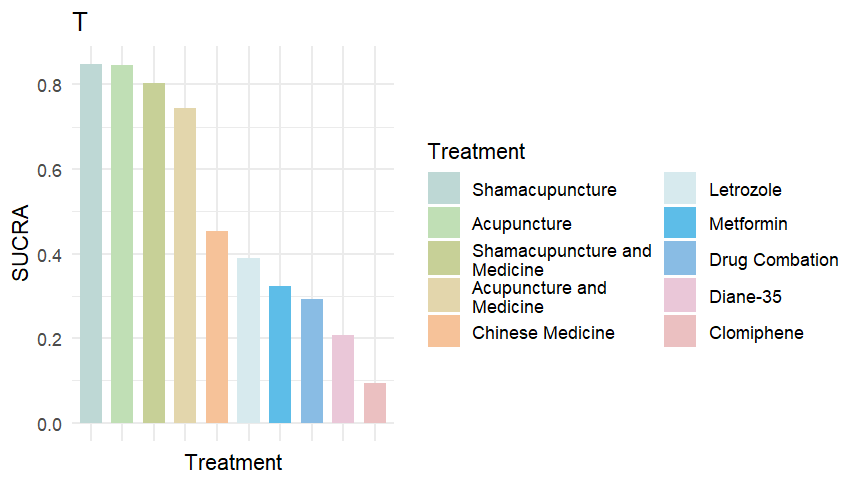
**

**FIGURE9. 3. Histogram plot of the network meta-analyses for LH**

**
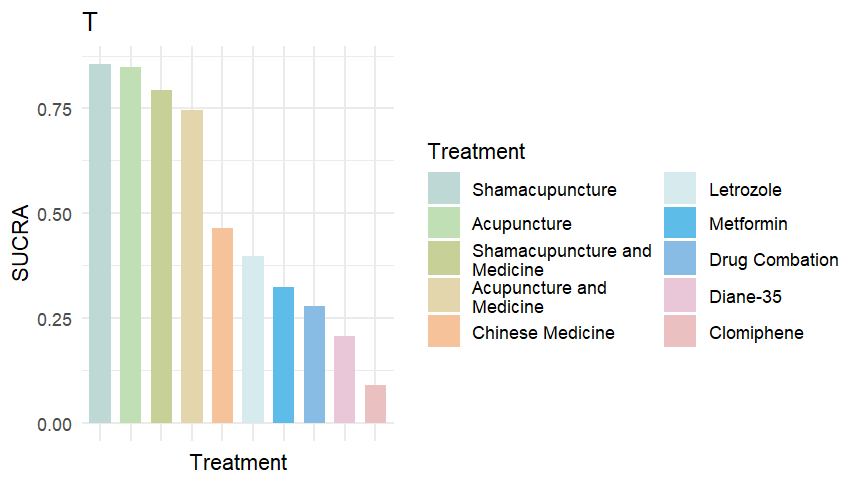
**

**FIGURE9. 4. Histogram plot of the network meta-analyses for FSH**

**
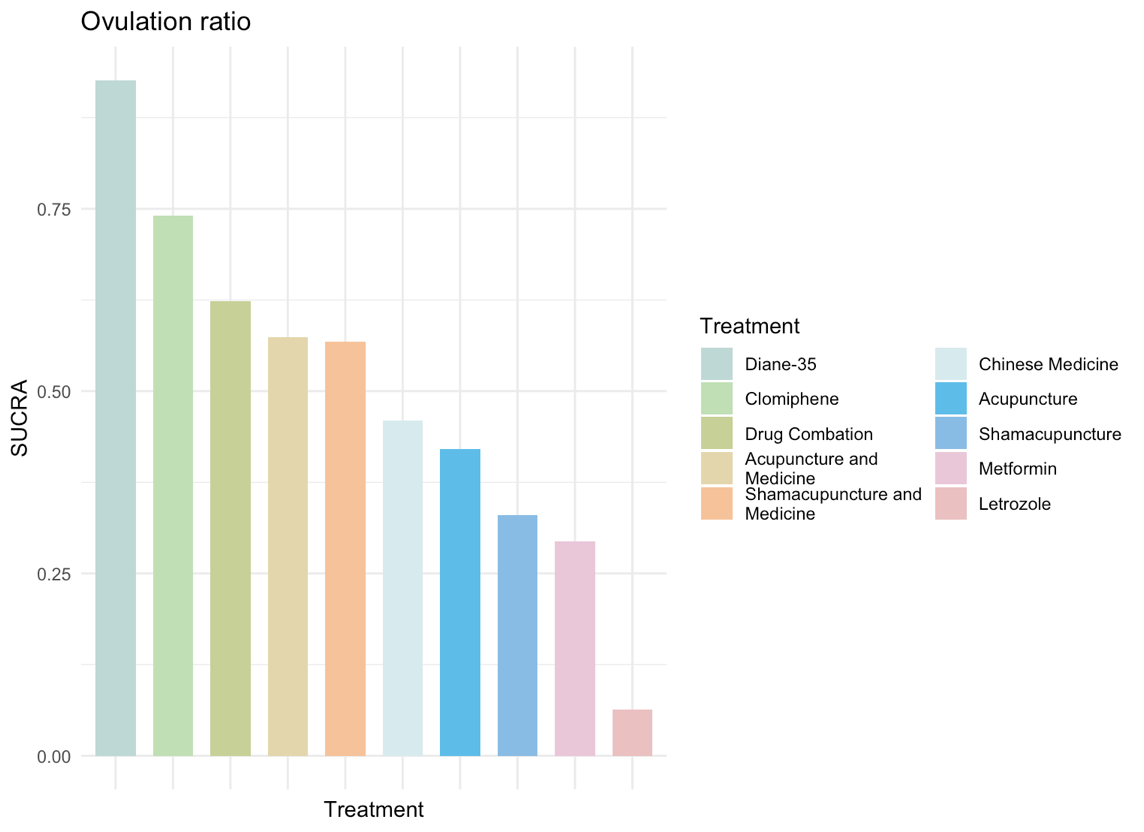
**

**FIGURE9. 5. Histogram plot of the network meta-analyses for LH: FSH**

**
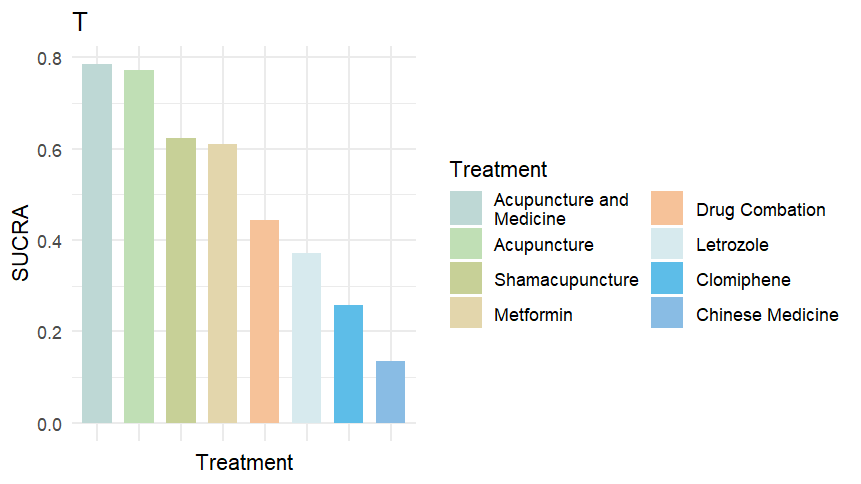
**

**FIGURE9. 6. Histogram plot of the network meta-analyses for T**

**
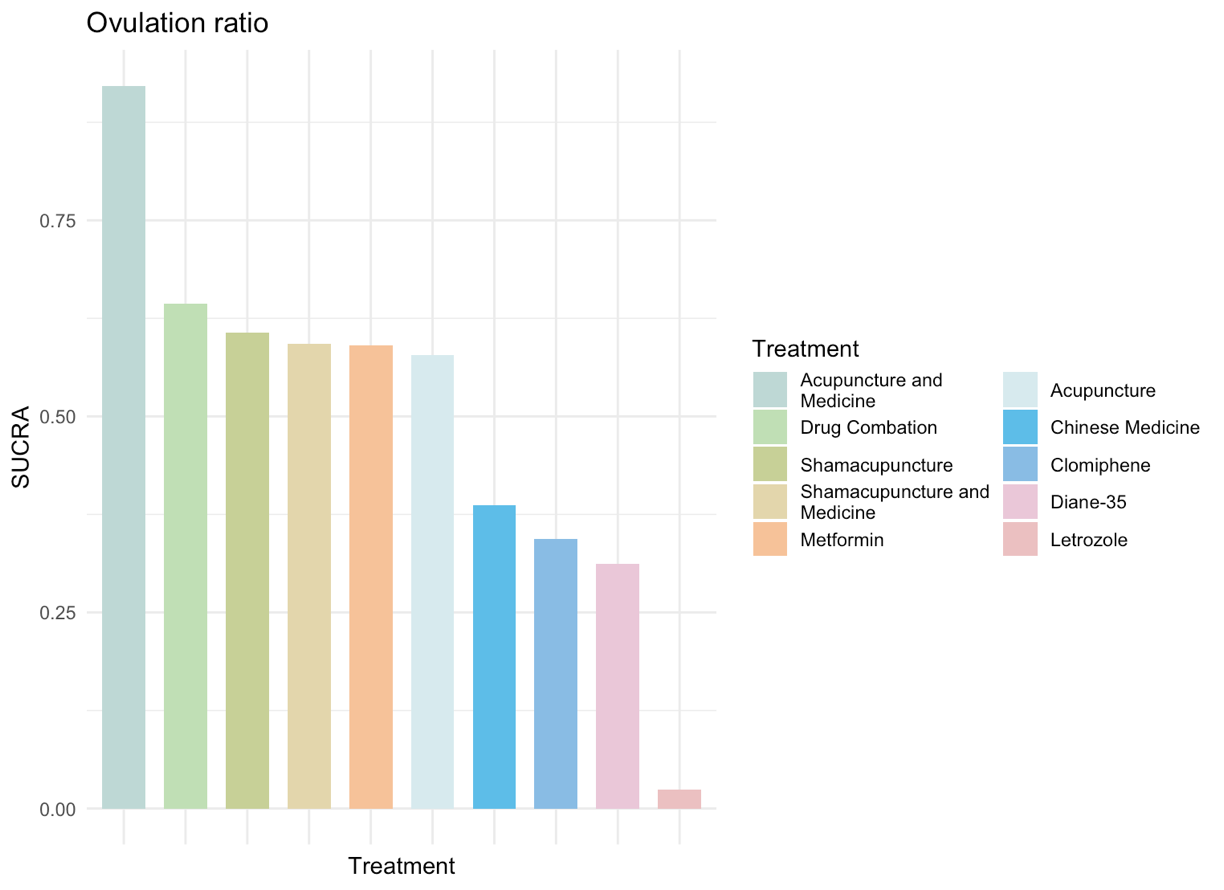
**

1. **Supplemental file 10. Exploratory acupuncture dose-response**
   1. **Supplemental file 10.1. Connect:**

**FIGURE 10.1. 1. Treatment-level network for acupoints**

**
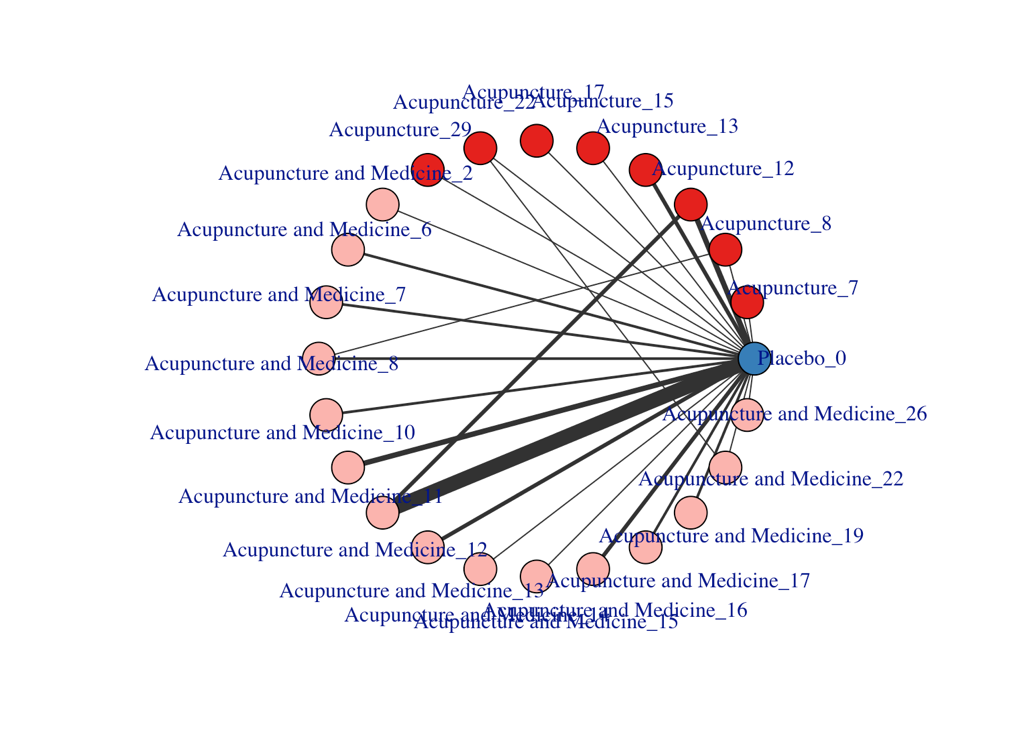
**

**FIGURE 10.1. 2. Treatment-level network for acupuncture frequency**

**
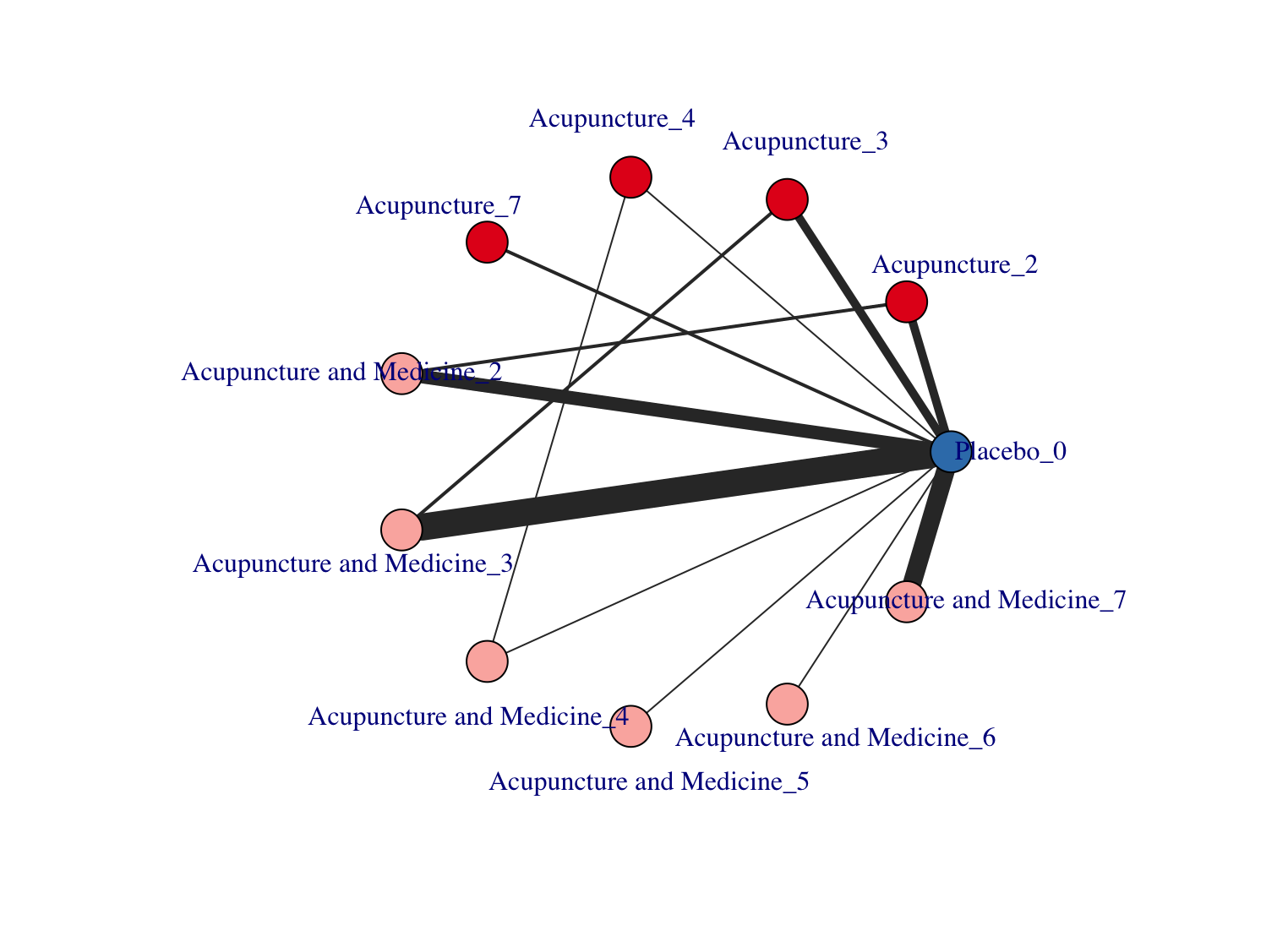
**

**FIGURE 10.1. 3. Treatment-level network for treatment duration**

**
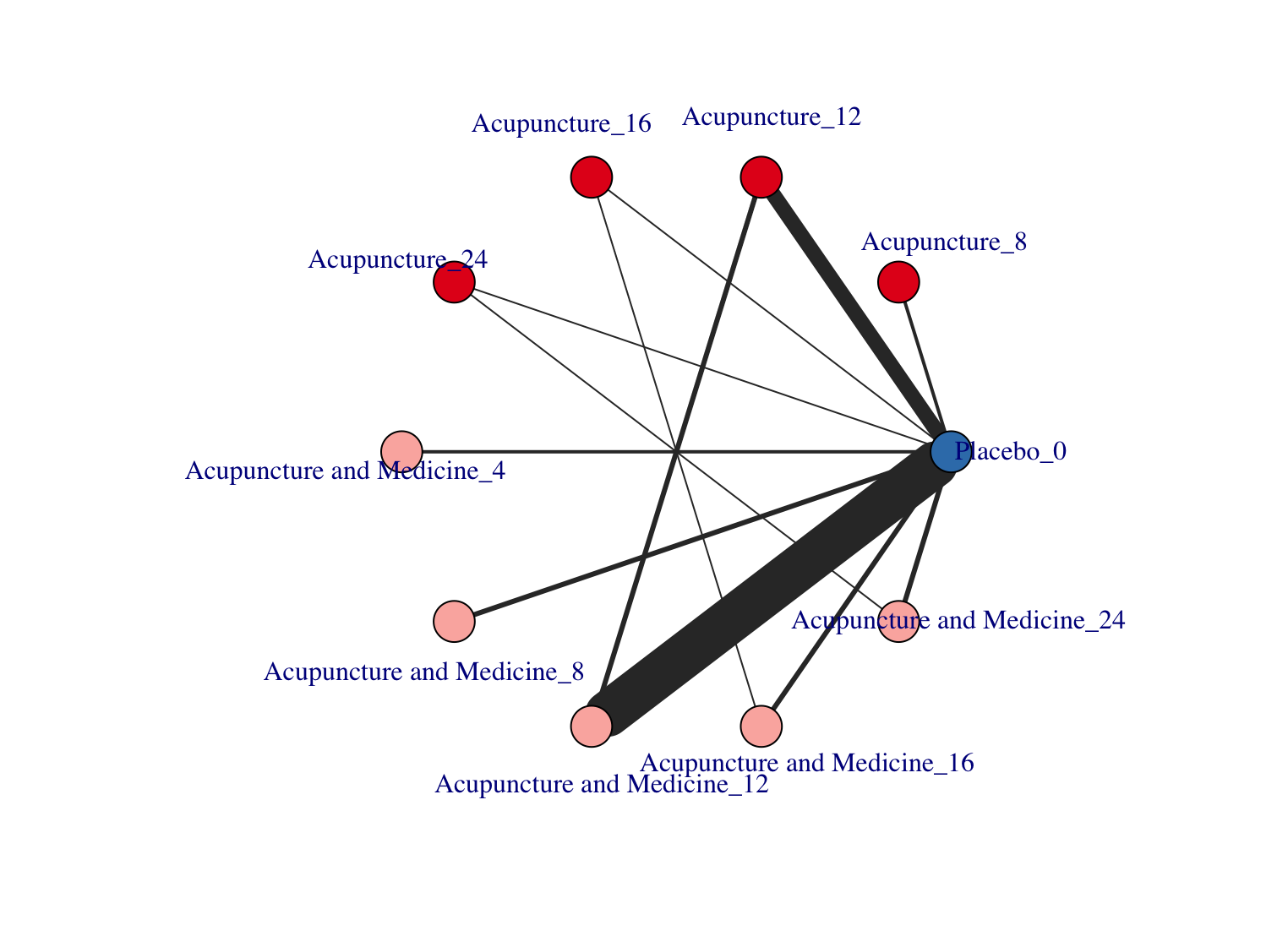
**

**FIGURE 10.1. 4. Treatment-level network for retaining time**

**
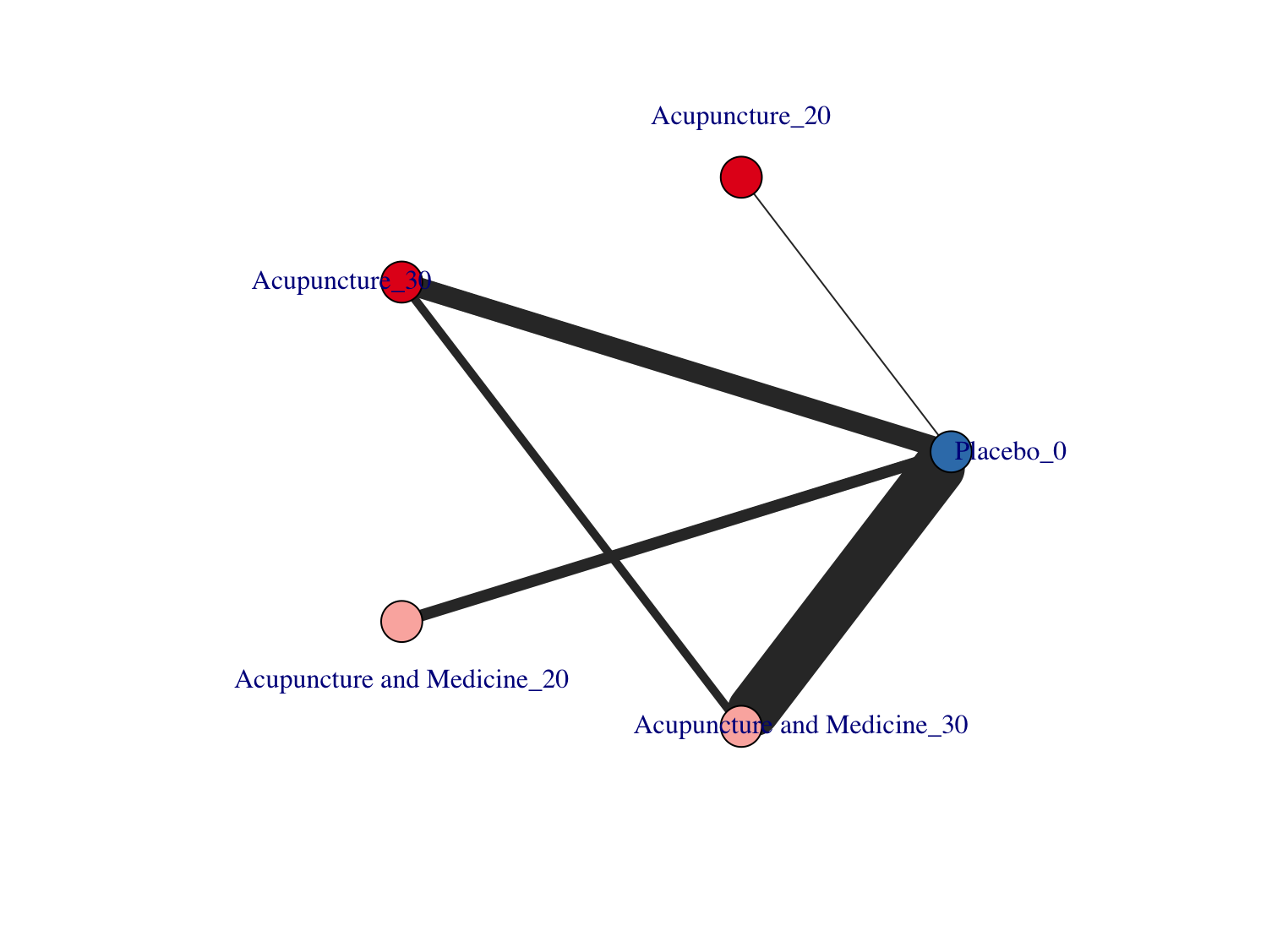
**

- 1. **Supplemental file 10.2.** **Consistency:**

Consistency analysis was conducted by comparing network effect sizes (i.e., estimates under the consistency model) with those from the Unrelated Mean Effects (UME) model (i.e., pairwise effect estimates). Methodologically, we assessed whether model fit, the number of estimated parameters in the network, and deviance information criterion (DIC) values were comparable between the two models, which would indicate adequate agreement. The comparative evaluation of these parameters demonstrated good consistency between the models.

**TABLE10.2. 1.Consistent and UME models fit comparison for acupoints**

| **Model** | **pV** | **Residual deviance** | **Deviance** | **DIC** | **SD** |
| --- | --- | --- | --- | --- | --- |
| **Consistent** | 85.1 | 88.445 | 490.498 | 575.6 | 0.488 |
| **UME** | 83.1 | 90.182 | 492.235 | 575.4 | 0.574 |

**TABLE10.2. 2. Consistent and UME models fit comparison for acupuncture frequency**

| **Model** | **pV** | **Residual deviance** | **Deviance** | **DIC** | **SD** |
| --- | --- | --- | --- | --- | --- |
| **Consistent** | 84.2 | 88.061 | 490.114 | 574.4 | 0.445 |
| **UME** | 85.7 | 91.852 | 493.906 | 569.6 | 0.701 |

**TABLE10.2. 3. Consistent and UME models fit comparison for treatment duration**

| **Model** | **pV** | **Residual deviance** | **Deviance** | **DIC** | **SD** |
| --- | --- | --- | --- | --- | --- |
| **Consistent** | 89.2 | 89.679 | 491.732 | 580.9 | 0.445 |
| **UME** | 89.0 | 90.413 | 492.466 | 581.5 | 0.588 |

TABLE10.2. 4. **Consistent and UME models fit comparison for retaining time**

| **Model** | **pV** | **Residual deviance** | **Deviance** | **DIC** | **SD** |
| --- | --- | --- | --- | --- | --- |
| **Consistent** | 83.9 | 87.341 | 489.394 | 573.3 | 0.486 |
| **UME** | 81.2 | 89.080 | 491.133 | 572.3 | 0.588 |

- 1. **Supplemental file 10.2. Transitivity:**

Network meta-analyses (NMAs) are predicated on the assumption of potential indirect/mixed comparisons, implying consistency between direct and indirect evidence in estimating treatment effects, subject to variability under a random-effects model framework. This assumption is analogous to heterogeneity in conventional meta-analyses. In accordance with prior methodological recommendations, transitivity was evaluated at the treatment level within the network. Transitivity was assessed using the MBNMA node-splitting approach, which partitions and compares the contributions of direct and indirect evidence for specific treatment contrasts.

**FIGURE 10.3. 1. Node-splitting analysis (density plot) for duration**

**
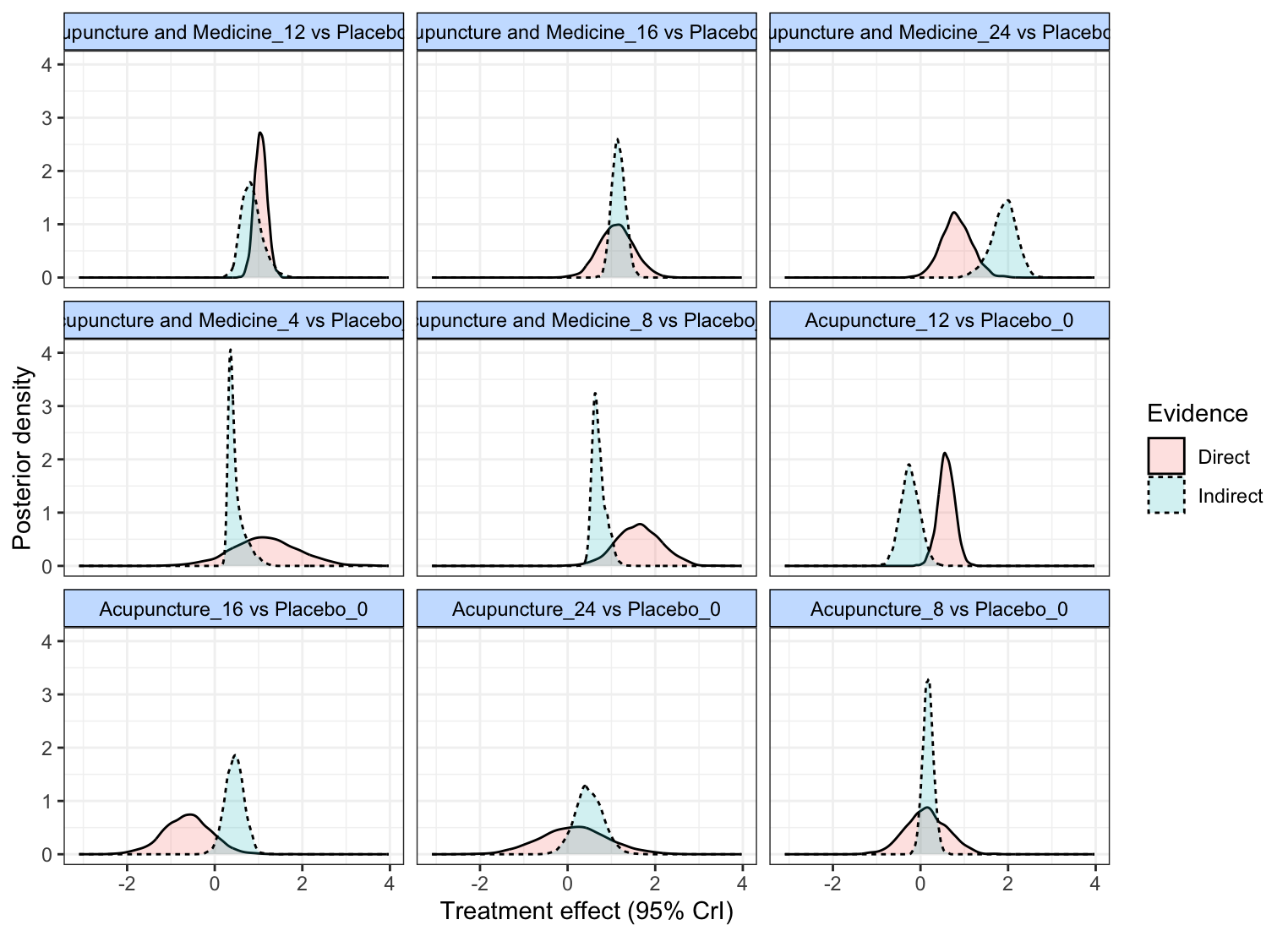
**

**FIGURE 10.3. 2. Node-splitting analysis (density plot) for acupoints**

**
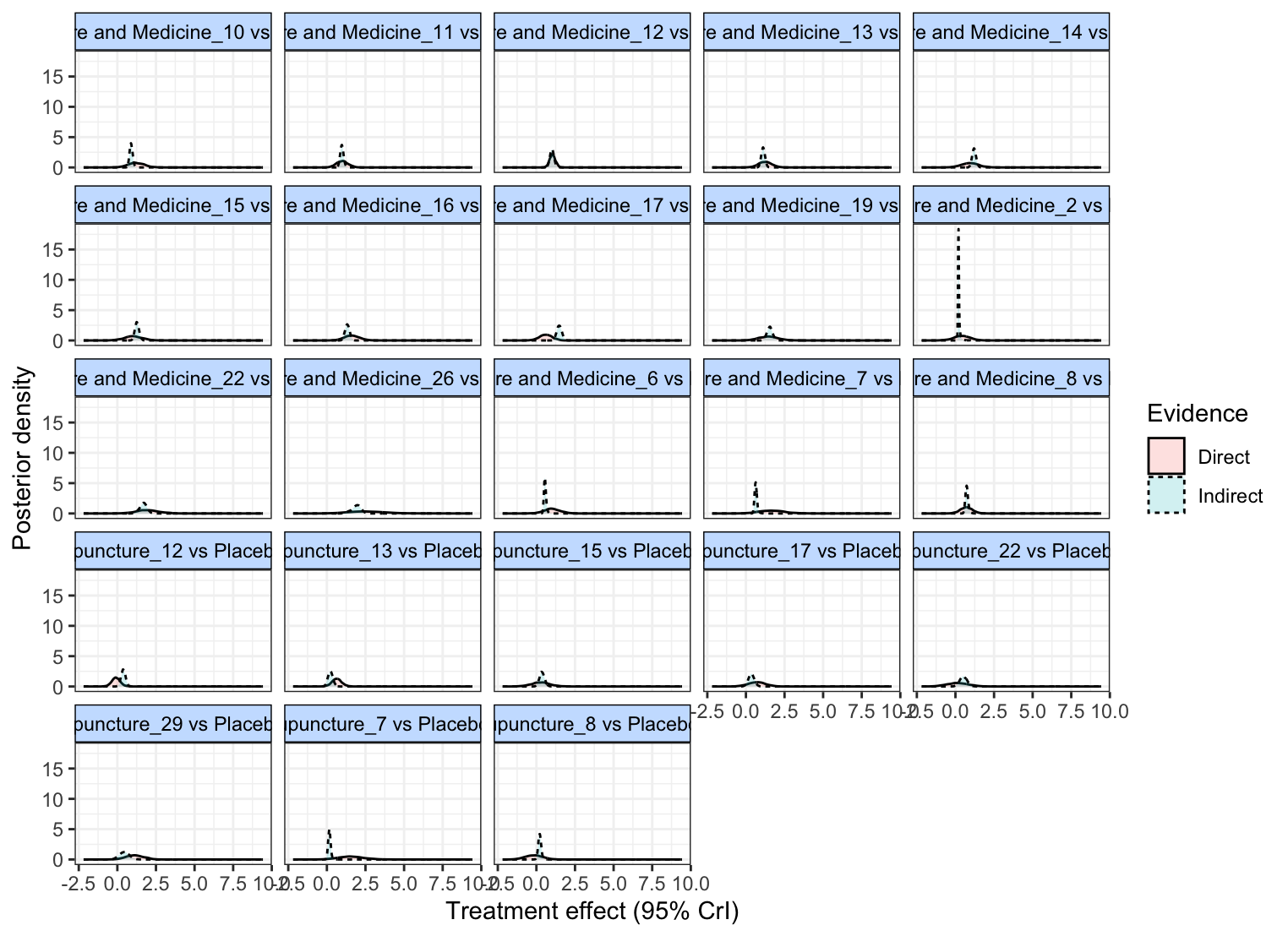
**

**FIGURE 10.3. 3. Node-splitting analysis (density plot) for acupuncture frequency**

**
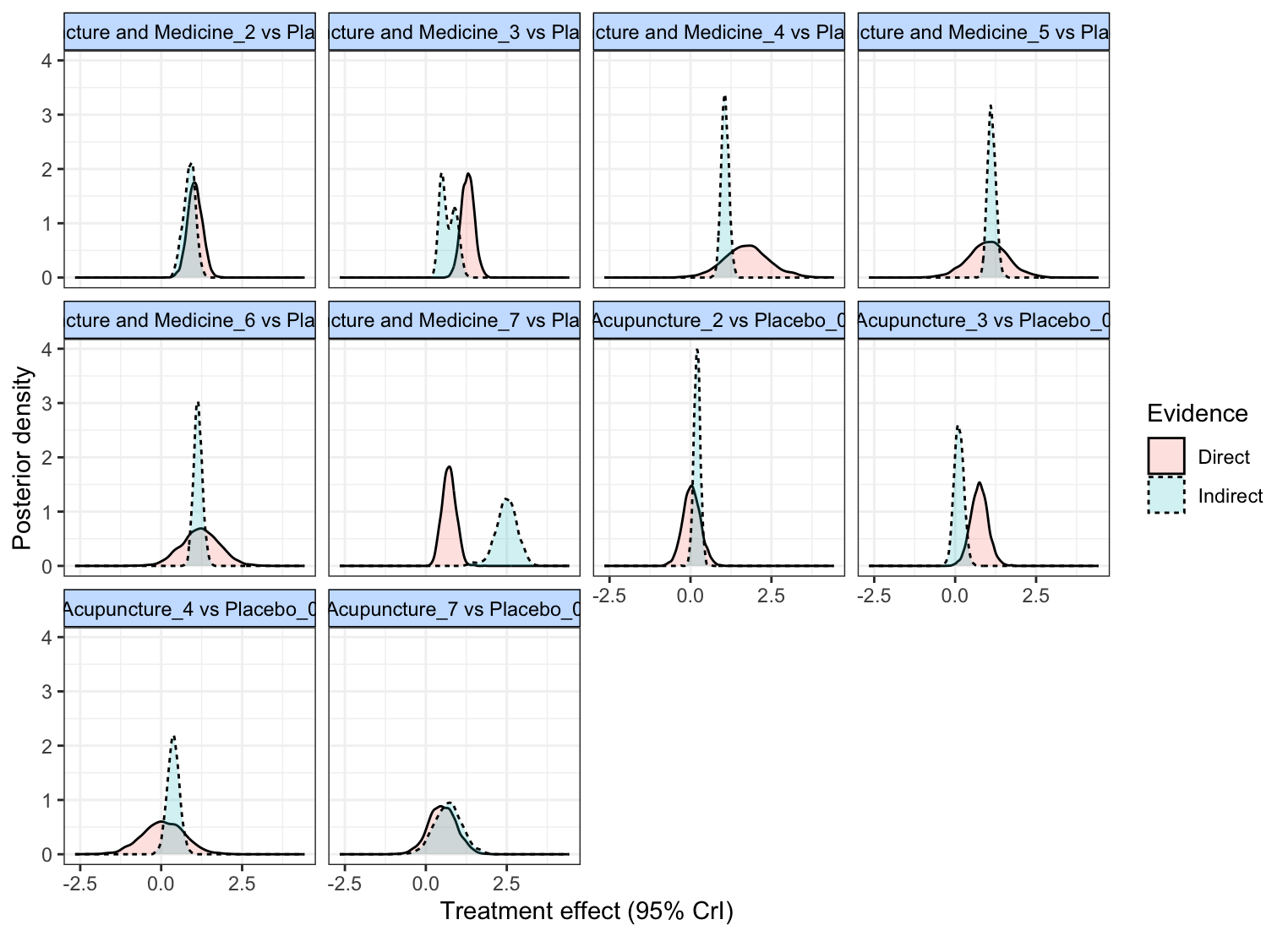
**

**FIGURE 10.3. 4. Node-splitting analysis (density plot) for retaining time**

**
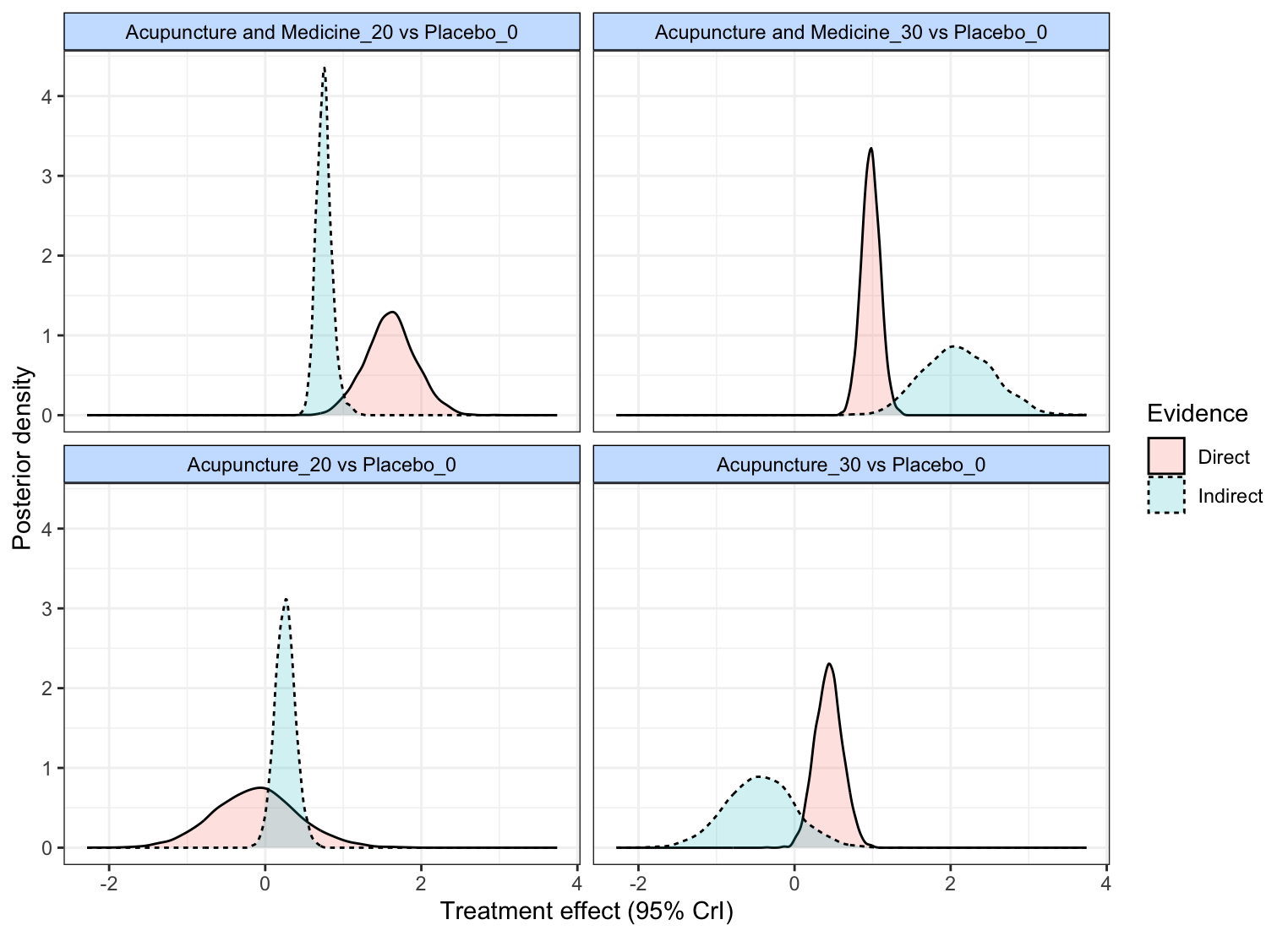
**

- 1. **. Supplemental file 10.4. Models fit comparison**

Different dosage levels of acupuncture and acupuncture combined with herbal medicine were analyzed as independent and unrelated interventions in a partitioned network meta-analysis (split-NMA). This step facilitates identification of the most suitable functional form for the data, which should then be applied in subsequent model-based network meta-analyses (MBNMAs).

**TABLE10.4. 1. Models fit comparison for duration**

| MODEL | DIC | SD | DEVLANCE | RESIDUAL DEVLANCE | PV |
| --- | --- | --- | --- | --- | --- |
| **EMAX**  **(Random Treatment Effect）** | **575.7** | **0.543** | **490.687** | **88.634** | **85.0** |
| EMAX  (Common Treatment Effect） | 635.4 | NA | 585.006 | 182.952 | 50.4 |
| Splines and knots  (Random Treatment Effect) | 577.3 | 0.443 | 491.323 | 89.270 | 86.0 |
| Splines and knots  (Common Treatment Effect) | 598.6 | NA | 544.559 | 142.506 | 54.0 |
| Linear  (Random Treatment Effect) | 581.3 | 0.593 | 492.195 | 90.142 | 89.1 |
| Linear  (Common Treatment Effect) | 666.3 | NA | 621.129 | 219.076 | 45.1 |
| Duser  (Random Treatment Effect) | 580.7 | 0.490 | 491.30.9 | 89.256 | 89.4 |
| Duser  (Common Treatment Effect) | 619.3 | NA | 571.869 | 169.816 | 47.4 |

**TABLE10.4. 2. Models fit comparison for acupuncture frequency**

| MODEL | DIC | SD | DEVLANCE | RESIDUAL DEVLANCE | PV |
| --- | --- | --- | --- | --- | --- |
| EMAX  (Random Treatment Effect） | 580.3 | 0.501 | 490.728 | 88.675 | 89.6 |
| EMAX  (Common Treatment Effect） | 624.9 | NA | 576.851 | 174.798 | 47.7 |
| **Splines and knots**  **(Random Treatment Effect)** | **575.9** | **0.428** | **490.083** | **88.030** | **82.4** |
| Splines and knots  (Common Treatment Effect) | 598.2 | NA | 544.970 | 142.917 | 53.3 |
| Linear  (Random Treatment Effect) | 584.2 | 0.698 | 493.990 | 91.937 | 90.3 |
| Linear  (Common Treatment Effect) | 698.8 | NA | 653.876 | 251.823 | 45.0 |
| Duser  (Random Treatment Effect) | 577.3 | 0.448 | 489.951 | 87.893 | 87.4 |
| Duser  (Common Treatment Effect) | 606.5 | NA | 560.163 | 158.110 | 46.4 |

**TABLE10.4. 3. Models fit comparison for acupoints**

| MODEL | DIC | SD | DEVLANCE | RESIDUAL DEVLANCE | PV |
| --- | --- | --- | --- | --- | --- |
| **EMAX**  **(Random Treatment Effect）** | **568.5** | **0.476** | **489.476** | **87.423** | **79.0** |
| EMAX  (Common Treatment Effect） | 618.1 | NA | 571.109 | 169.056 | 47.0 |
| Splines and knots  (Random Treatment Effect) | 572.9 | 0.482 | 490.519 | 88.466 | 82.4 |
| Splines and knots  (Common Treatment Effect) | 611.7 | NA | 563.017 | 160.964 | 48.7 |
| Linear  (Random Treatment Effect) | 576.5 | 0.535 | 492.112 | 90.059 | 84.4 |
| Linear  (Common Treatment Effect) | 626.4 | NA | 578.211 | 176.168 | 48.2 |
| Duser  (Random Treatment Effect) | 576.3 | 0.472 | 490.620 | 88.566 | 85.7 |
| Duser  (Common Treatment Effect) | 616.8 | NA | 568.608 | 166.554 | 48.2 |

**TABLE10.4. 4. Models fit comparison for retaining time**

| MODEL | DIC | SD | DEVLANCE | RESIDUAL DEVLANCE | PV |
| --- | --- | --- | --- | --- | --- |
| EMAX  (Random Treatment Effect） | 578.1 | 0.502 | 490.607 | 88.553 | 87.5 |
| EMAX  (Common Treatment Effect） | 629.9 | NA | 583.308 | 181.255 | 46.6 |
| Splines and knots  (Random Treatment Effect) | 573.2 | 0.486 | 489.324 | 87.270 | 83.9 |
| Splines and knots  (Common Treatment Effect) | 619.8 | NA | 574.122 | 172.069 | 45.7 |
| Linear  (Random Treatment Effect) | 578.9 | 0.509 | 491.615 | 89.562 | 87.3 |
| Linear  (Common Treatment Effect) | 633.8 | NA | 588.344 | 186.291 | 45.4 |
| **Duser**  **(Random Treatment Effect)** | **566.8** | **0.487** | **488.999** | **86.946** | **77.8** |
| Duser  (Common Treatment Effect) | 620.7 | NA | 574.048 | 171.995 | 46.7 |

1. **Supplemental file 11. GRADE Framework**

**Risk of bias:** The evidence was downgraded by one level if more than 25% of participants originated from studies with a high risk of bias (i.e., one or more bias domains were rated as high risk).

**Inconsistency:** The evidence was downgraded by one level if substantial heterogeneity (I² statistic >50%) was identified and remained unexplained by study design, population differences, or intervention variations.

**Imprecision:** The evidence level was downgraded by one grade if the 95% confidence interval (CI) width for relative risk reduction (RRR) or relative risk increase (RRI) exceeded 25 percentage points.

**Publication bias:** The evidence quality was downgraded by one grade if a high likelihood of publication bias existed. Publication bias was defined as either funnel plot asymmetry indicating potential publication bias or >25% of participants being derived from small-scale studies (sample size <30).

1. **Supplemental file 12. Summary of findings**

**TABLE 12. 1. Summary of findings in acupuncture versus sham acupuncture**

| **Summary of findings:** | | | | | | |
| --- | --- | --- | --- | --- | --- | --- |
| **Acupuncture compared to Sham acupuncture for PCOS** | | | | | | |
| **Patient or population:** PCOS  **Setting:**  **Intervention:** Acupuncture  **Comparison:** Sham acupuncture | | | | | | |
| Outcomes | **Anticipated absolute effects^*^** (95% CI) | | Relative effect (95% CI) | № of participants (studies) | Certainty of the evidence (GRADE) | Comments |
|  | **Risk with Sham acupuncture** | **Risk with Acupuncture** |  |  |  |  |
| Ovulation rates | 484 per 1,000 | **557 per 1,000** (504 to 615) | **RR 1.15** (1.04 to 1.27) | 1325 (4 RCTs) | ⨁⨁⨁⨁ High | NA |
| FSH |  | SMD **0.01 higher** (1.1 lower to 1.11 higher) | - | 600 (3 RCTs) | ⨁⨁⨁◯ Moderate^a^ | NA |
| LH |  | SMD **0.17 higher** (0.4 lower to 0.73 higher) | - | 600 (3 RCTs) | ⨁⨁◯◯ Low^a,b^ | NA |
| ***The risk in the intervention group** (and its 95% confidence interval) is based on the assumed risk in the comparison group and the **relative effect** of the intervention (and its 95% CI). **CI:** confidence interval; **RR:** risk ratio | | | | | | |

**TABLE 12. 2.** **Summary of findings in acupuncture versus medicine**

| **Acupuncture compared to Medicine for PCOS** | | | | | | |
| --- | --- | --- | --- | --- | --- | --- |
| **Patient or population:** PCOS  **Intervention:** Acupuncture  **Comparison:** Medicine | | | | | | |
| Outcomes | **Anticipated absolute effects^*^** (95% CI) | | Relative effect (95% CI) | № of participants (studies) | Certainty of the evidence (GRADE) | Comments |
|  | **Risk with Medicine** | **Risk with Acupuncture** |  |  |  |  |
| Ovulation rates | 624 per 1,000 | **693 per 1,000** (649 to 749) | **RR 1.11** (1.04 to 1.20) | 1422 (8 RCTs) | ⨁⨁⨁⨁ High | NA |
| FSH | - | SMD **0.12 SD higher** (0.1 lower to 0.34 higher) | - | 326 (4 RCTs) | ⨁⨁⨁◯ Moderate^a^ | NA |
| LH | - | SMD **0.38 SD higher** (0.02 lower to 0.79 higher) | - | 539 (6 RCTs) | ⨁⨁◯◯ Low^a,b^ | NA |
| T | - | SMD **0.22 SD higher** (0.21 lower to 0.65 higher) | - | 597 (7 RCTs) | ⨁⨁◯◯ Low^a,b^ | NA |
| ***The risk in the intervention group** (and its 95% confidence interval) is based on the assumed risk in the comparison group and the **relative effect** of the intervention (and its 95% CI).  **CI:** confidence interval; **RR:** risk ratio; **SMD:** standardised mean difference | | | | | | |

**TABLE 12. 3. Summary of findings in acupuncture and Chinese medicine versus Western medicine**

|  | | | | | | |
| --- | --- | --- | --- | --- | --- | --- |
| **Acupuncture and Chinese medicine compared to Western medicine for PCOS** | | | | | | |
| **Patient or population:** PCOS  **Setting:**  **Intervention:** Acupuncture and Chinese medicine  **Comparison:** Western medicine | | | | | | |
| Outcomes | **Anticipated absolute effects^*^** (95% CI) | | Relative effect (95% CI) | № of participants (studies) | Certainty of the evidence (GRADE) | Comments |
|  | **Risk with Western medicine** | **Risk with Acupuncture and Chinese medicine** |  |  |  |  |
| Ovulation rates | 645 per 1,000 | **819 per 1,000** (722 to 922) | **RR 1.27** (1.12 to 1.43) | 998 (8 RCTs) | ⨁⨁⨁◯ Moderate^a^ | NA |
| FSH | - | SMD **0.7 SD lower** (2.61 lower to 1.22 higher) | - | 341 (4 RCTs) | ⨁⨁◯◯ Low^b,c^ | NA |
| LH | - | SMD **1.49 SD higher** (0.73 higher to 2.25 higher) | - | 571 (7 RCTs) | ⨁⨁◯◯ Low^b,d^ | NA |
| LH:FSH | - | SMD **0.91 SD higher** (0.08 higher to 1.74 higher) | - | 220 (3 RCTs) | ⨁⨁◯◯ Low^b,d^ | NA |
| T | - | SMD **0.34 SD higher** (0.53 lower to 1.2 higher) | - | 411 (5 RCTs) | ⨁◯◯◯ Very low^b,d^ | NA |

**TABLE 12. 4. Summary of findings in acupuncture versus blank**

|  | | | | | | |
| --- | --- | --- | --- | --- | --- | --- |
| **Acupuncture compared to Blank for PCOS** | | | | | | |
| **Patient or population:** PCOS  **Intervention:** Acupuncture  **Comparison:** Blank | | | | | | |
| Outcomes | **Anticipated absolute effects^*^** (95% CI) | | Relative effect (95% CI) | № of participants (studies) | Certainty of the evidence (GRADE) | Comments |
|  | **Risk with Blank** | **Risk with Acupuncture** |  |  |  |  |
| Ovulation rates | 646 per 1,000 | **794 per 1,000** (756 to 840) | **RR 1.23** (1.17 to 1.30) | 4577 (27 RCTs) | ⨁⨁⨁◯ Moderate^a^ | NA |
| BMI |  | MD **2.81 higher** (1.65 higher to 3.97 higher) | - | 354 (4 RCTs) | ⨁⨁◯◯ Low^b,c^ | NA |
| FSH | - | SMD **0.09 SD higher** (0.53 lower to 0.72 higher) | - | 1149 (14 RCTs) | ⨁⨁◯◯ Low^b,c^ | NA |
| LH | - | SMD **1.17 SD higher** (0.71 higher to 1.62 higher) | - | 1473 (17 RCTs) | ⨁⨁◯◯ Low^a,b^ | NA |
| LH:FSH | - | SMD **0.91 SD higher** (0.08 higher to 1.74 higher) | - | 594 (6 RCTs) | ⨁⨁⨁◯ Moderate^b^ | NA |
| T | - | SMD **0.71 SD higher** (0.31 higher to 1.1 higher) | - | 1290 (15 RCTs) | ⨁⨁⨁◯ Moderate^b^ | NA |

1. **Supplemental file 13. Network diagram of the network meta-analysis**

**FIGURE13. 1. Network diagram of the network meta-analysis for Ovulation rates**


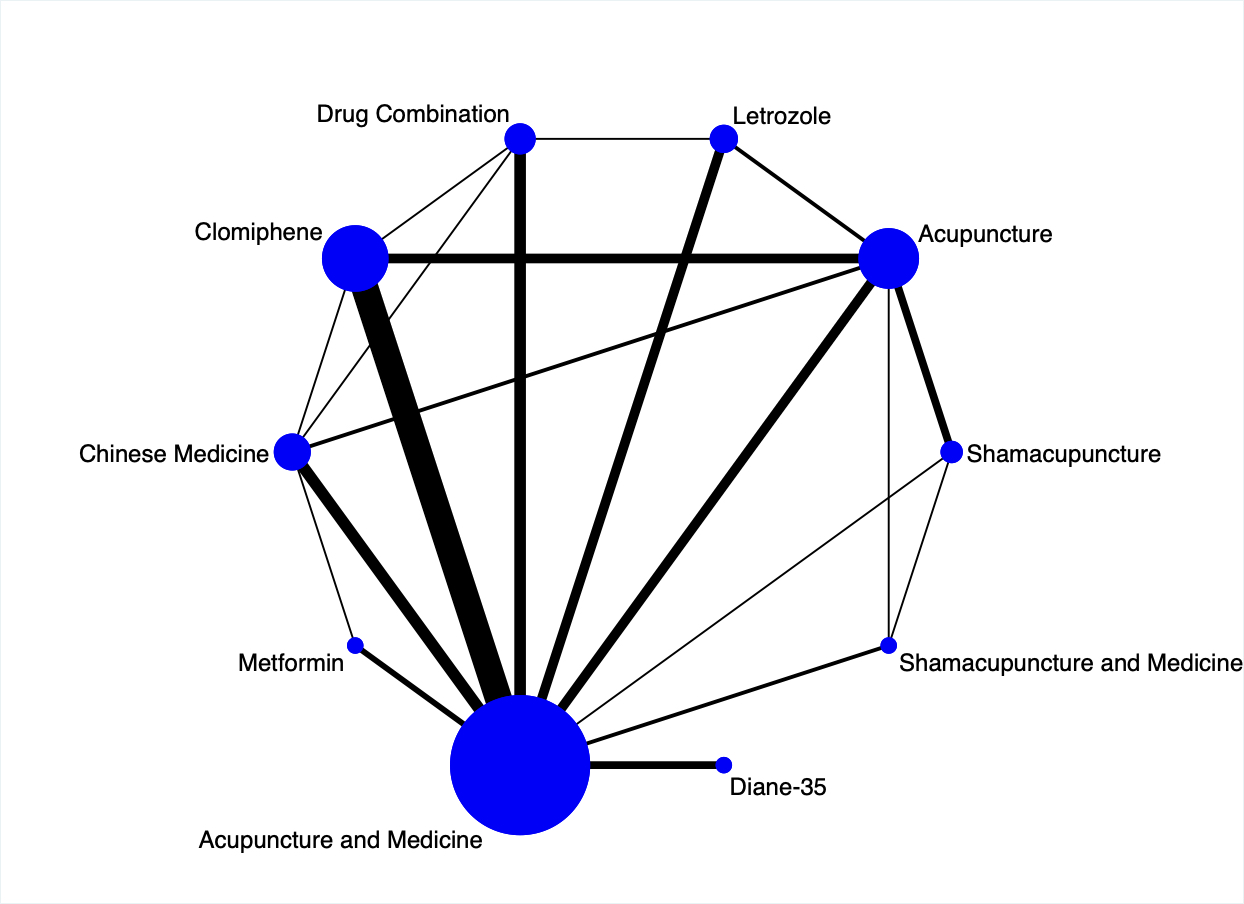


**FIGURE13. 2.Network diagram of the network meta-analysis for BMI**

**
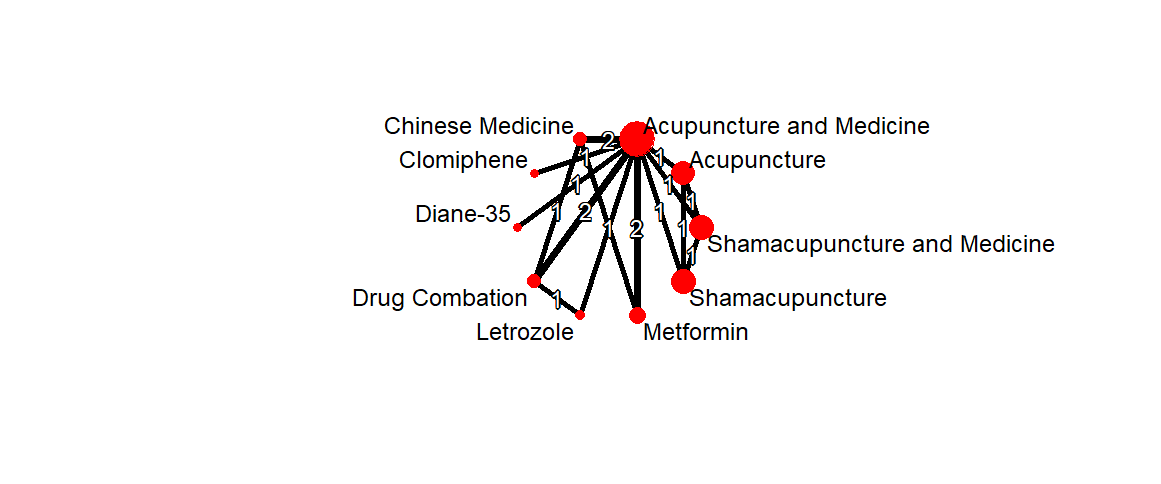
**

**FIGURE13. 3. Network diagram of the network meta-analysis for LH**

**
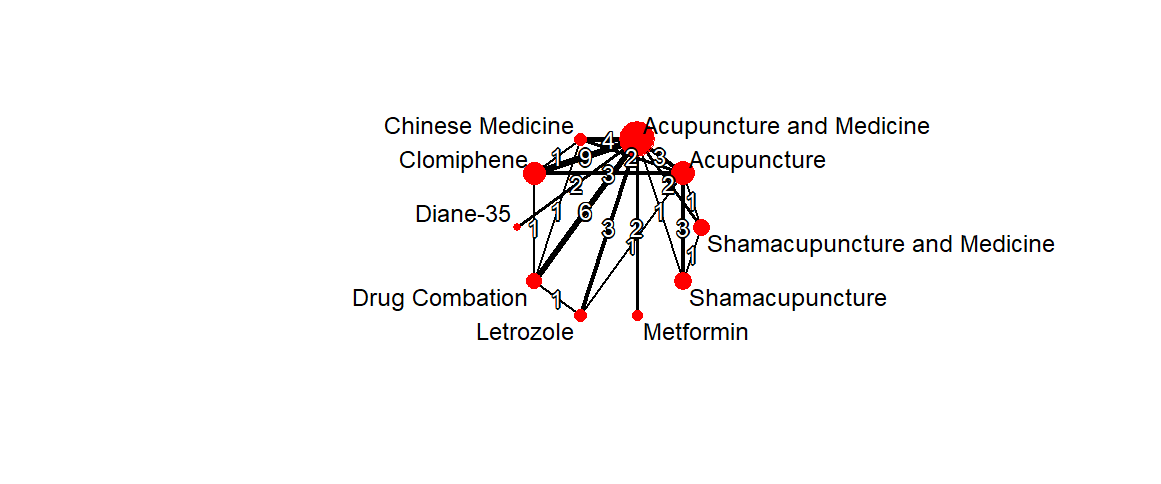
**

**FIGURE13. 4. Network diagram of the network meta-analysis for LH: FSH**

**
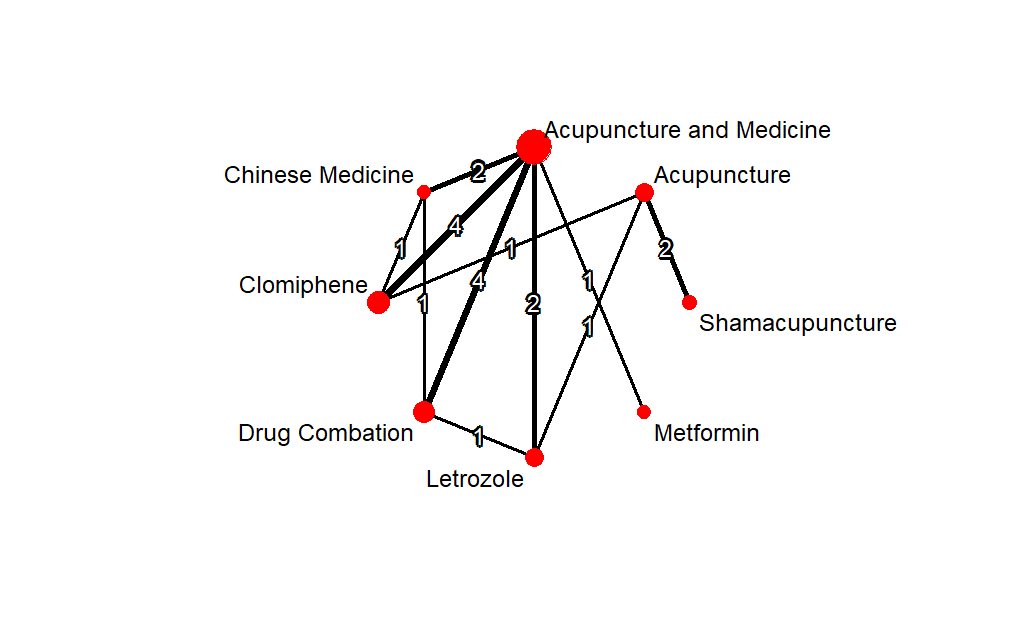
**

**FIGURE13. 5. Network diagram of the network meta-analysis for T**

**
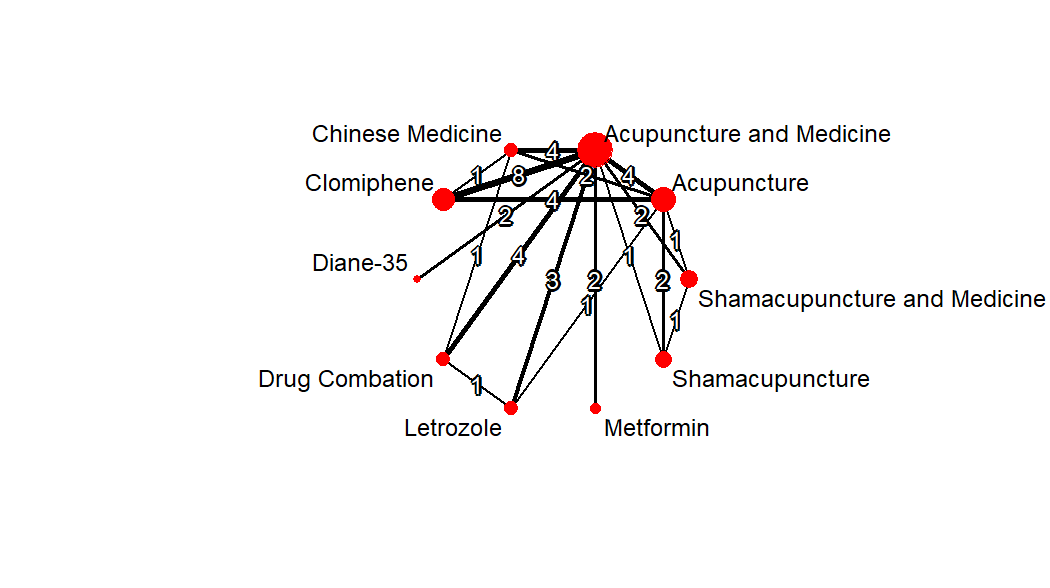
**

1. **Supplemental file 14. Forest plot of** **node splitting analysis**

**FIGURE14. 1.Forest plot of node splitting analysis for ovulation rates**

**FIGURE14. 2. Forest plot of node splitting analysis for FSH**

**
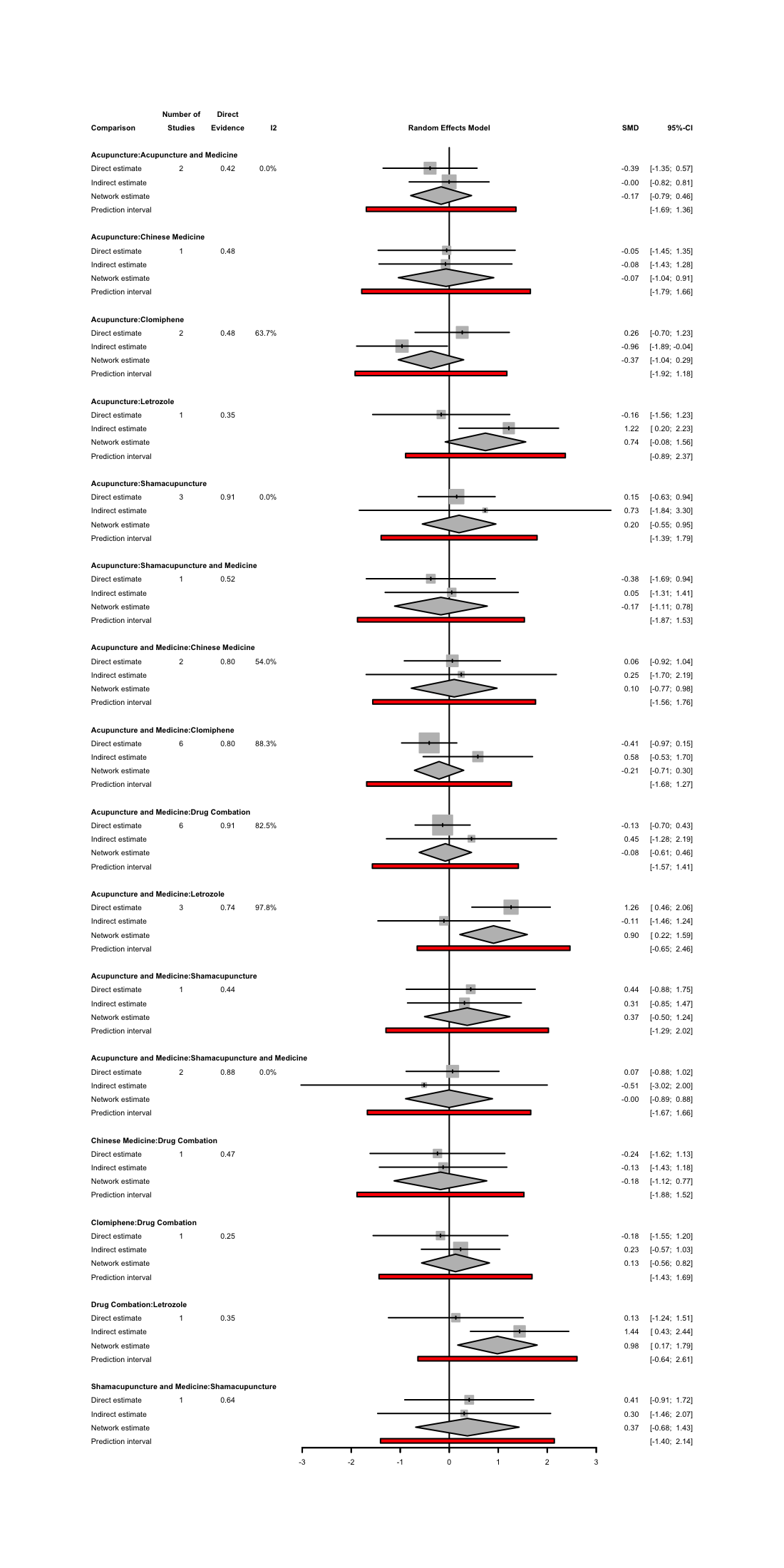
**

**FIGURE14. 3. Forest plot of node splitting analysis for T**

**
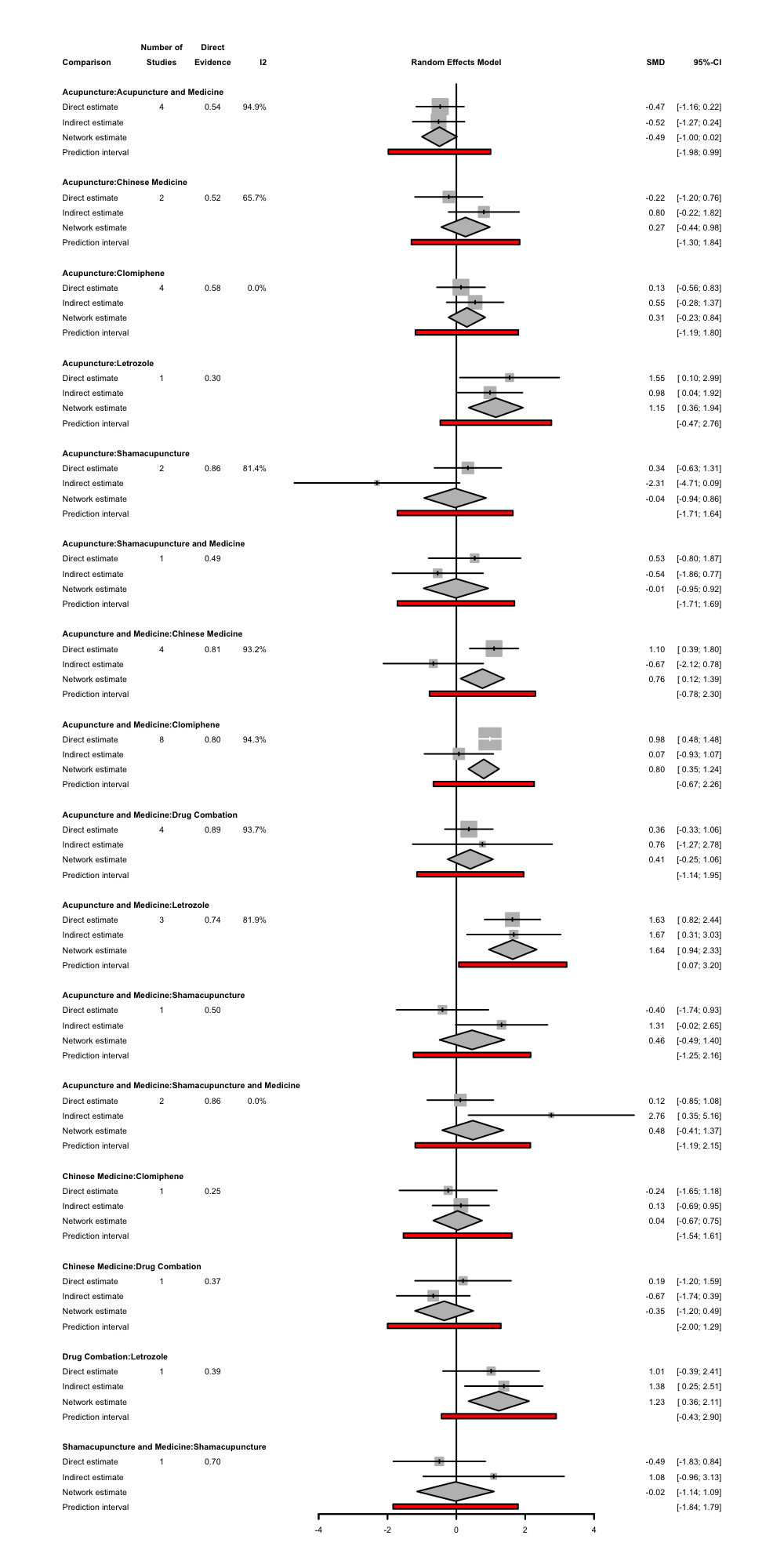
**

**FIGURE14. 4. Forest plot of node splitting analysis for BMI**

**
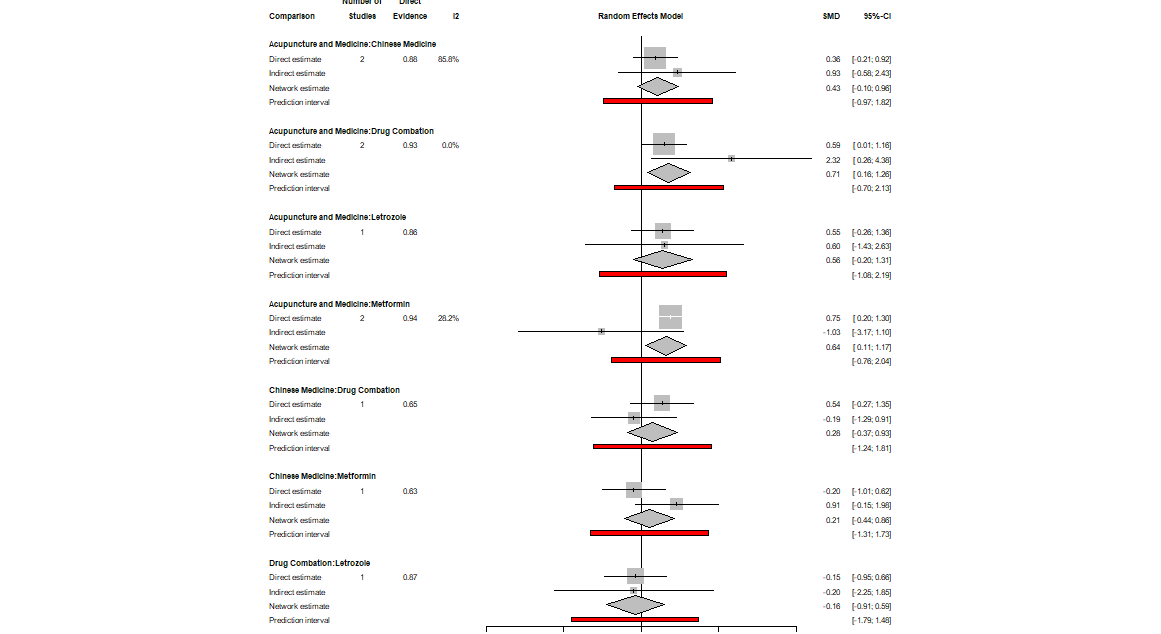
**

**FIGURE14. 5. Forest plot of node splitting analysis for LH**

**
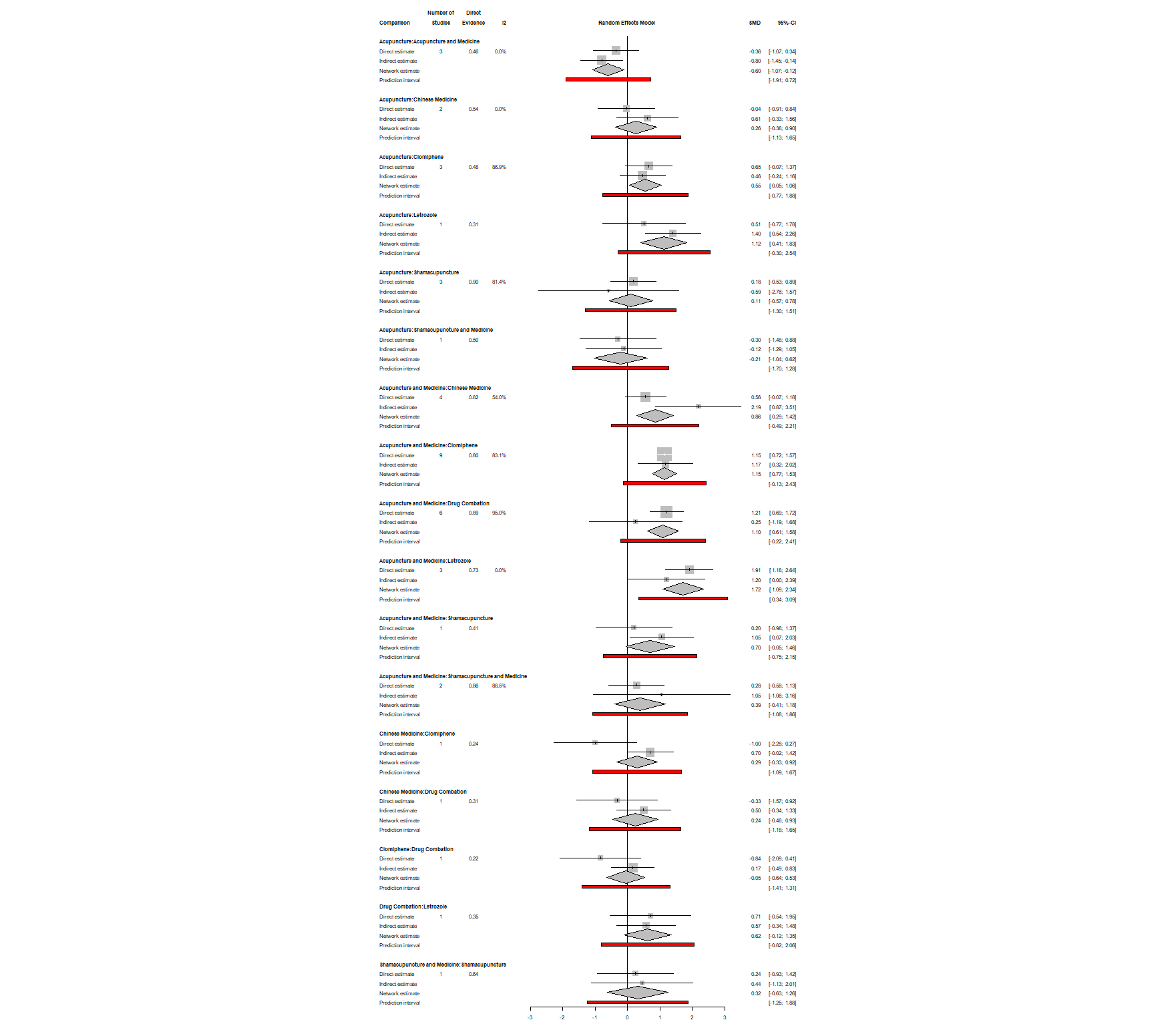
**

**FIGURE14. 6. Forest plot of node splitting analysis for LH: FSH**

**
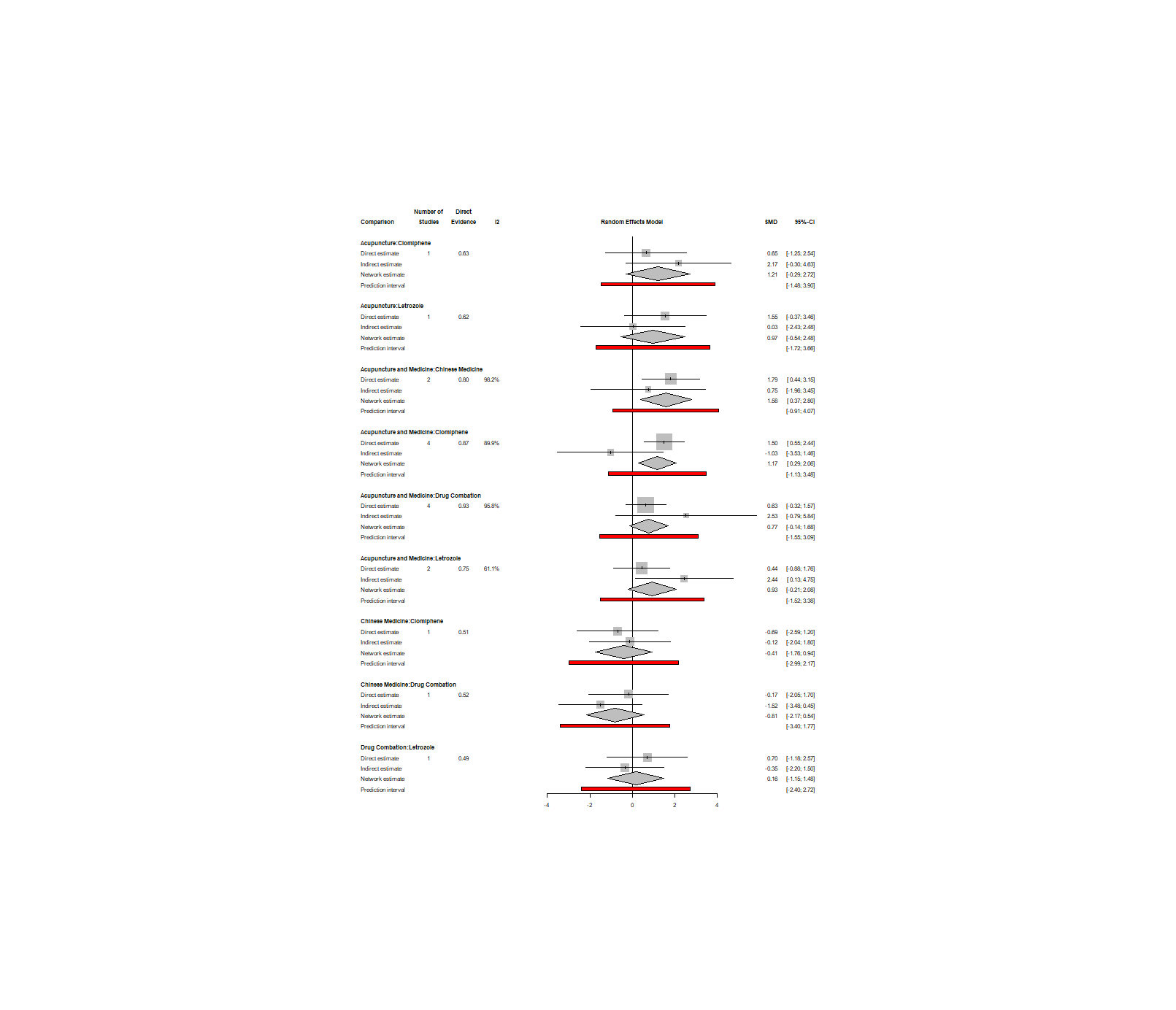
**

1. **Supplemental file 15. Forest plot of network meta-analysis compared with sham acupuncture**

**FIGURE 15. 1.Forest plot of network meta-analysis for ovulation rates**

**
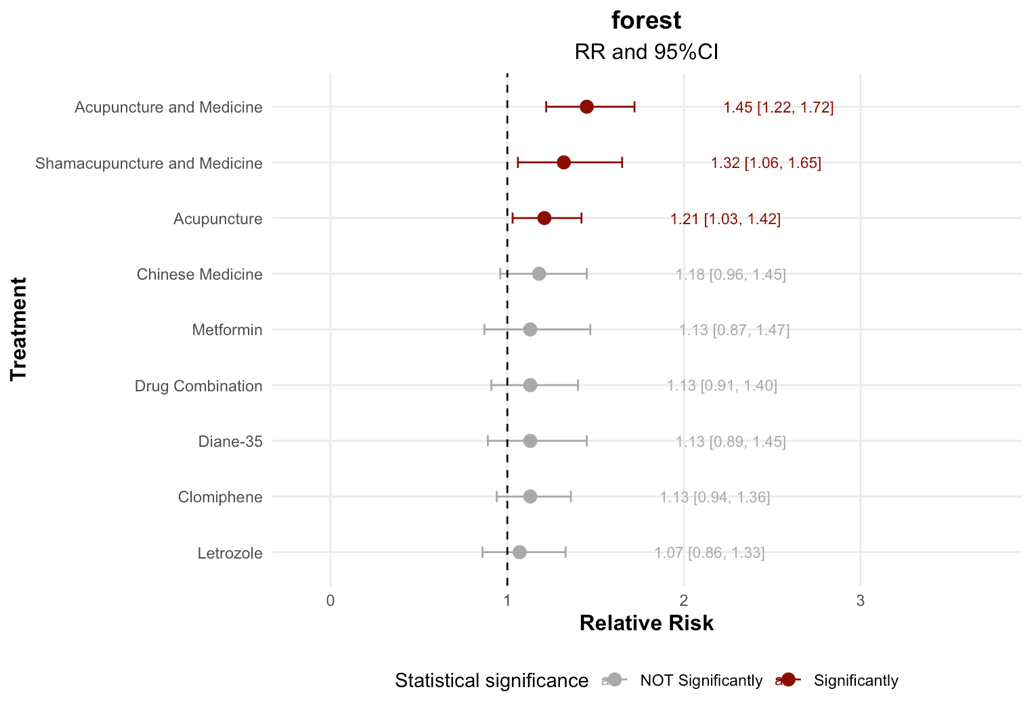
**

**FIGURE 15. 2. Forest plot of network meta-analysis for BMI**

**
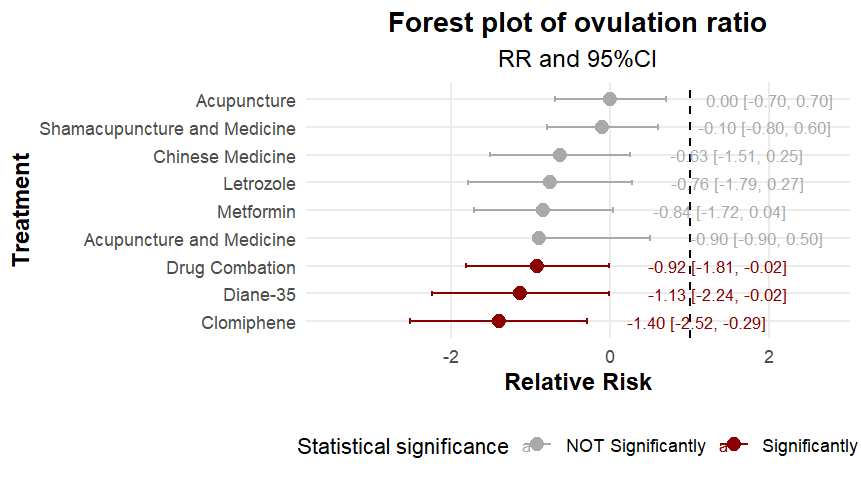
**

**FIGURE 15. 3. Forest plot of network meta-analysis for FSH**

**
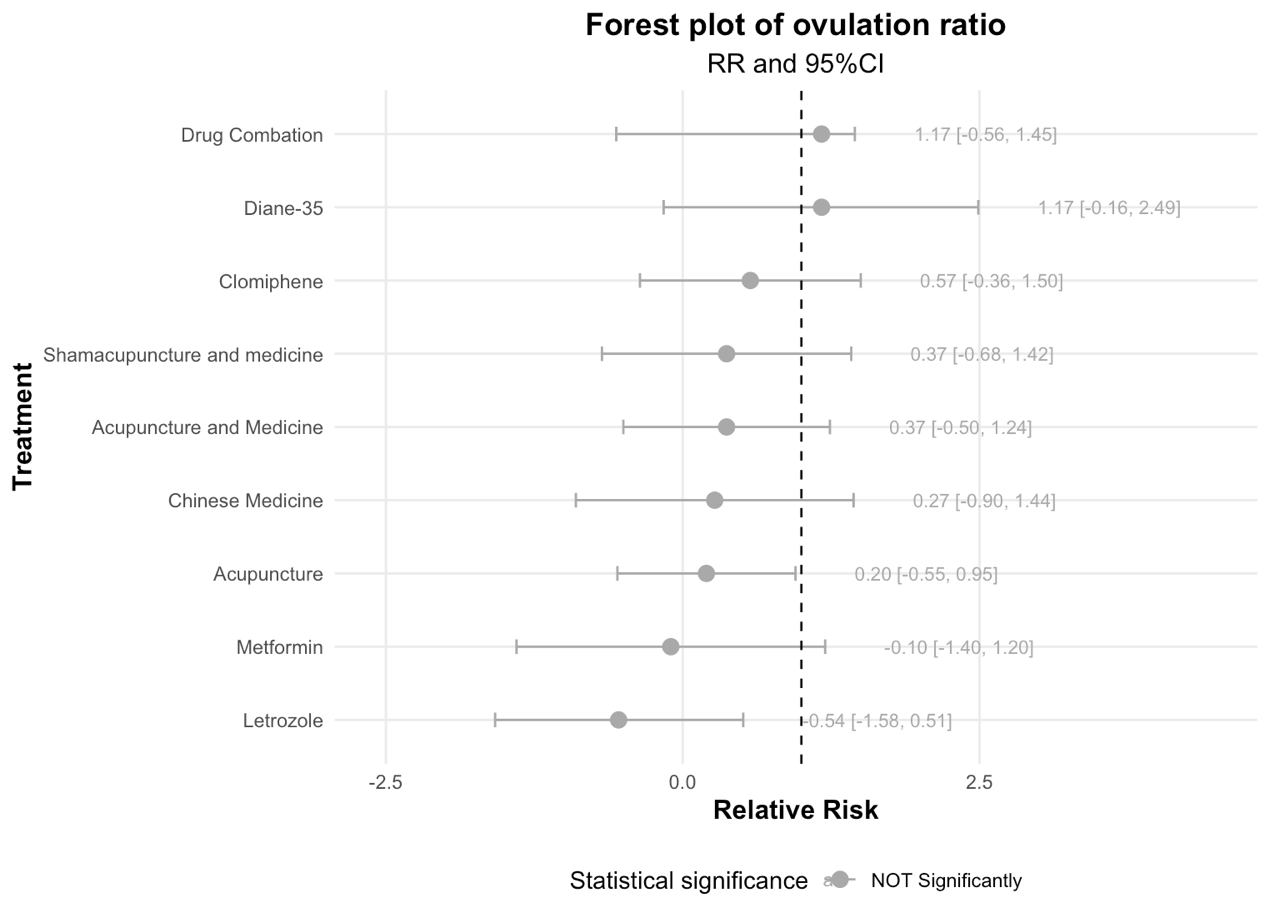
**

**FIGURE 15. 4. Forest plot of network meta-analysis for LH**

**
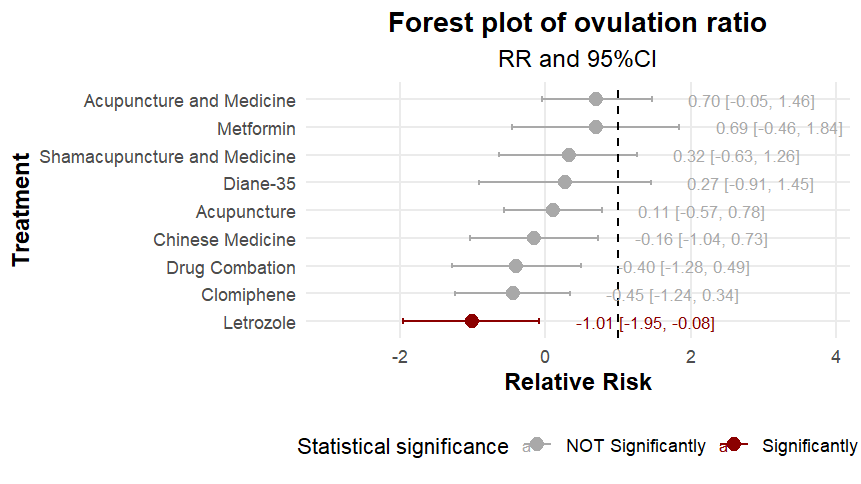
**

**FIGURE 15. 5. Forest plot of network meta-analysis for LH:FSH**

**
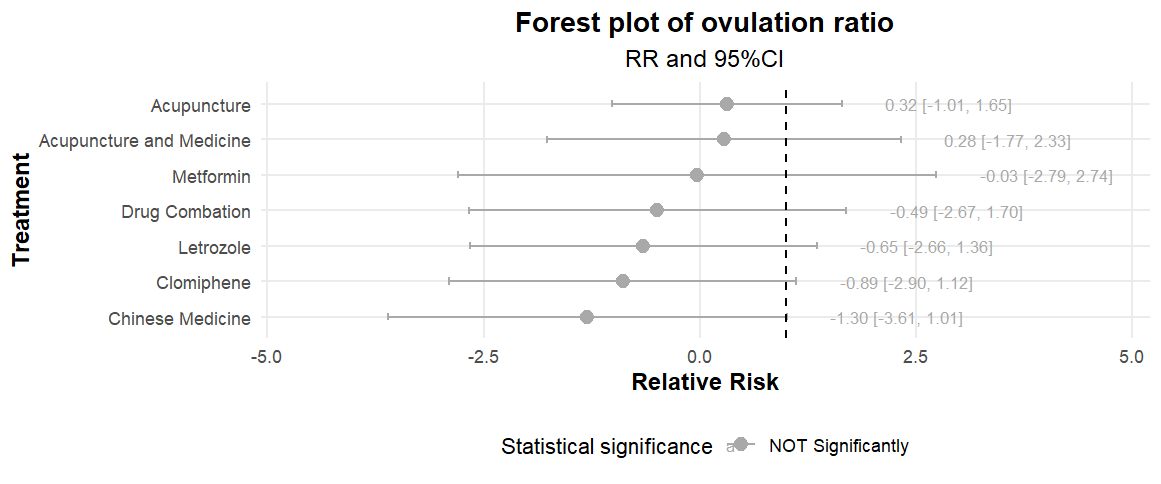
**

**FIGURE 15. 6. Forest plot of network meta-analysis for T**

**
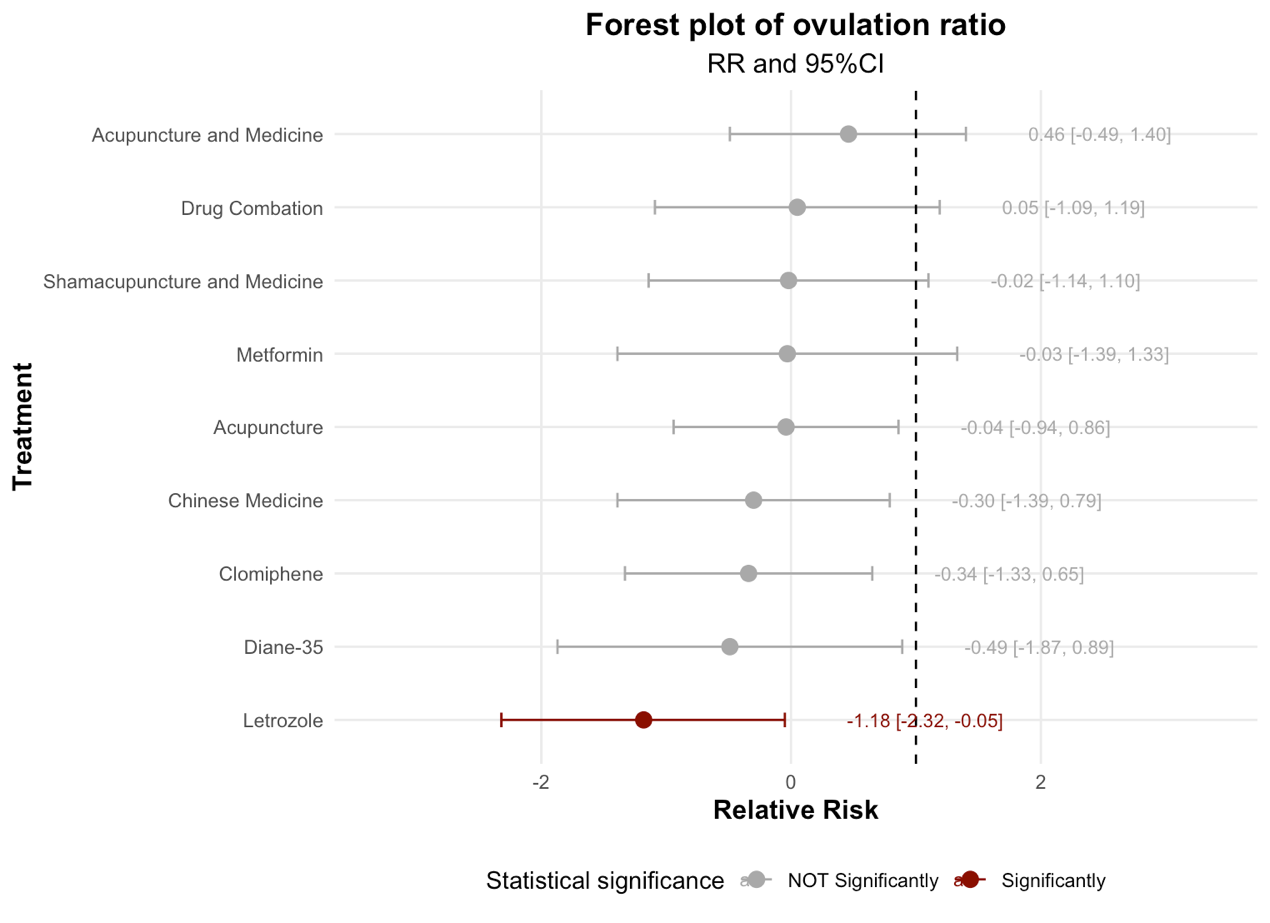
**
